# Supplementary material for: Novel Insights on Extracellular Electron Transfer Networks in the Desulfovibrionaceae Family: Unveiling the Potential Significance of Horizontal Gene Transfer
Source: Microorganisms. 2024 Aug 29;12(9):1796. doi: 10.3390/microorganisms12091796 (PMC11434368; doi:10.3390/microorganisms12091796)
Supplement: Supplementary file 1 [file microorganisms-12-01796-s001.zip › Figures.zip!/Figure_S10_phylogeny_genomic_context_OmpJ.pdf]

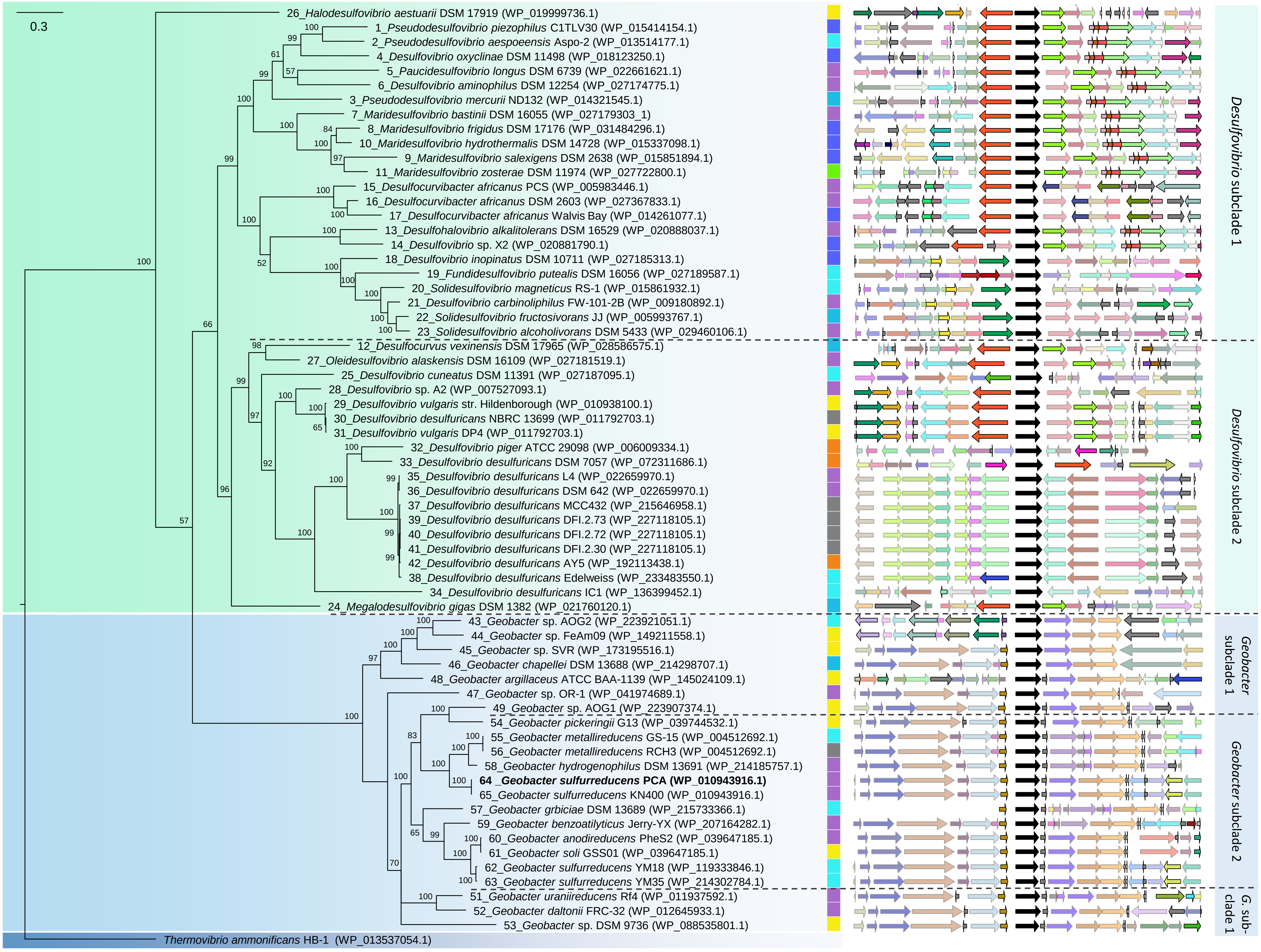

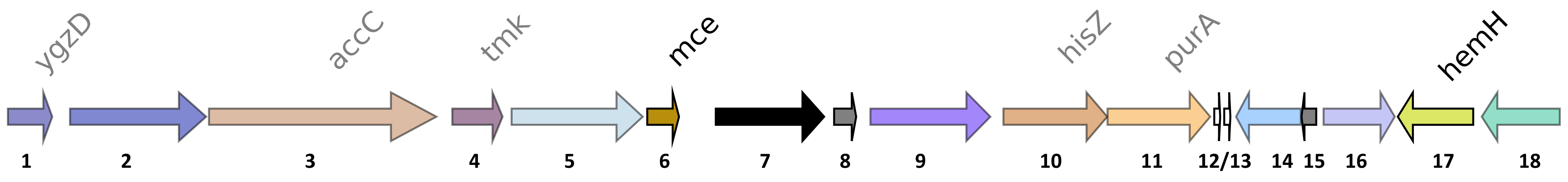

Nº	Accesion number	Product
1	WP_010943910.1	XRE family transcriptional regulator (ygzD)
2	WP_010943911.1	acetyl-CoA carboxylase carboxyltransferase subunit
3	WP_010943912.1	biotin carboxylase (accC)
4	WP_010943913.1	dTMP kinase (tmk)
5	WP_010943914.1	methylmalonyl-CoA mutase family protein
6	WP_010943915.1	methylmalonyl-CoA epimerase (mce)
**7**	**WP_010943916.1**	**histidine kinase (ompJ)**
8	WP_010943917.1	hypothetical protein
9	WP_010943918.1	VCBS repeat-containing protein

Nº	Accesion number	Product
10	WP_010943919.1	ATP phosphoribosyltransferase regulatory subunit (hisZ)
11	WP_010943920.1	adenylosuccinate synthase (purA)
12	NA	tRNA-Lys
13	NA	tRNA-Glu
14	WP_010943921.1	AAA family ATPase
15	WP_010943922.1	hypothetical protein
16	WP_010943923.1	protoglobin domain-containing protein
17	WP_010943924.1	Ferrochelatase (hemH)
18	WP_010943925.1	thiolase family protein
