# Supplementary material for: Novel Insights on Extracellular Electron Transfer Networks in the Desulfovibrionaceae Family: Unveiling the Potential Significance of Horizontal Gene Transfer
Source: Microorganisms. 2024 Aug 29;12(9):1796. doi: 10.3390/microorganisms12091796 (PMC11434368; doi:10.3390/microorganisms12091796)
Supplement: Supplementary file 1 [file microorganisms-12-01796-s001.zip › Figures.zip!/Figure_S11_Phylogeny_genomic_context_cymA.pdf]

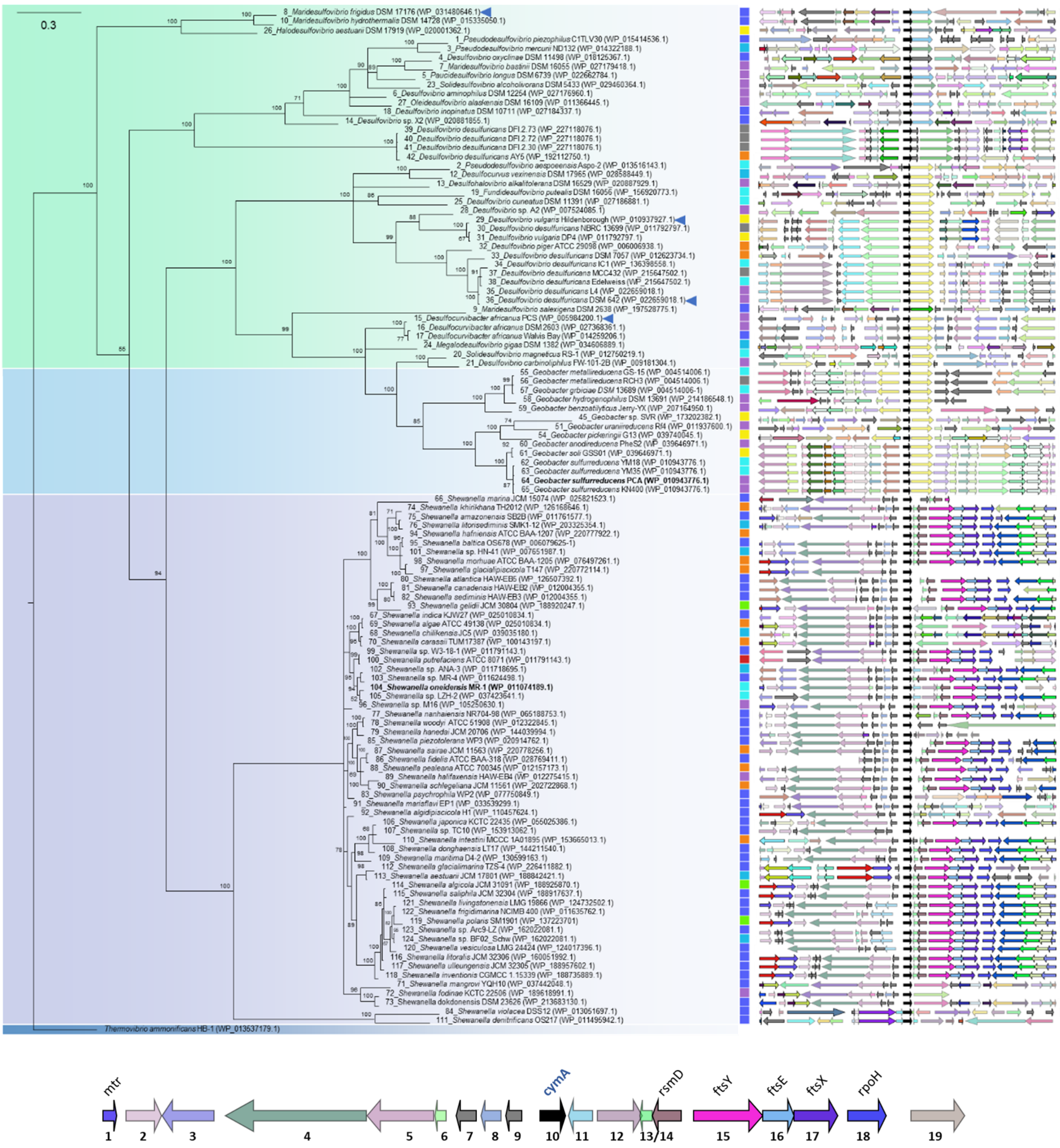

Nº	Accesion number	Product
1	WP_011074198.1	tryptophan permease (mtr)
2	WP_011074197.1	VOC family protein
3	WP_011074196.1	ribonuclease T2
4	WP_011074195.1	CusA/CzcA family heavy metal efflux RND transporter
5	WP_011074194.1	efflux RND transporter periplasmic adaptor subunit
6	WP_011074193.1	copper-exporting ATPase
7	WP_011074192.1	hypothetical protein
8	WP_011074191.1	DUF2057 family protein
9	WP_011074190.1	hypothetical protein
10	WP_011074189.1	NapC/NirT family cytochrome c (CymA)
11	WP_011074188.1	cysteine hydrolase
12	WP_011074187.1	helix-turn-helix domain-containing protein
13	WP_011074186.1	DUF1145 domain-containing protein
14	WP_011074185.1	16S rRNA (guanine(966)-N(2))-methyltransferase (RsmD)
15	WP_011074184.1	signal recognition particle-docking protein (FtsY)
16	WP_011074183.1	cell division ATP-binding protein (FtsE)
17	WP_011074182.1	permease-like cell division protein (FtsX)
18	WP_011074181.1	RNA polymerase sigma factor (RpoH)
19	WP_011070721.1	IS256-like element ISSod4 family transposase
