# Supplementary material for: Novel Insights on Extracellular Electron Transfer Networks in the Desulfovibrionaceae Family: Unveiling the Potential Significance of Horizontal Gene Transfer
Source: Microorganisms. 2024 Aug 29;12(9):1796. doi: 10.3390/microorganisms12091796 (PMC11434368; doi:10.3390/microorganisms12091796)
Supplement: Supplementary file 1 [file microorganisms-12-01796-s001.zip › Figures.zip!/Figure_S12_genomic_context_of_cymA_others_Desulfovibrio.pdf]

A

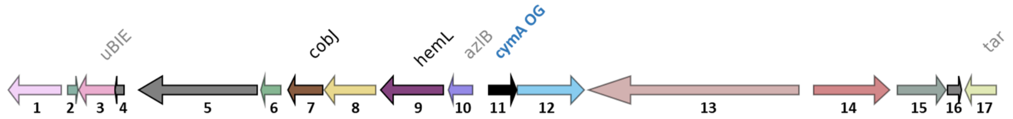

Nº	Accesion number	Product
1	WP_031480626.1	nucleotide sugar dehydrogenase
2	WP_031480628.1	DUF2065 domain-containing protein
3	WP_031480630.1	ubiquinone/menaquinone biosynthesis methyltransferase
4	WP_031480632.1	hypothetical protein
5	WP_031480634.1	hypothetical protein
6	WP_031480636.1	cytochrome c3 family protein
7	WP_051676927.1	precorrin-3B C(17)-methyltransferase (cobJ)
8	WP_034602860.1	cobalt-precorrin 5A hydrolase
9	WP_031480642.1	glutamate-1-semialdehyde 2%2C1-aminomutase (hemL)
10	WP_031480644.1	Lrp/AsnC family transcriptional regulator

Nº	Accesion number	Product
**11**	**WP_031480646.1**	**NapC/NirT family cytochrome c**
12	WP_034602861.1	multiheme c-type cytochrome
13	WP_051676928.1	glycosyl hydrolase family 18 protein
14	WP_031480652.1	sigma-54-dependent Fis family transcriptional regulator
15	WP_031480654.1	NAD(P)-dependent glycerol-3-phosphate dehydrogenase
16	WP_031480656.1	hypothetical protein
17	WP_031480658.1	chemotaxis protein

B

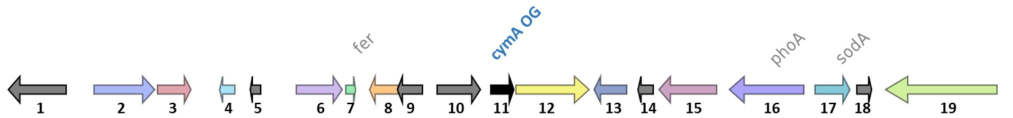

Nº	Accesion number	Product
1	WP_005984210.1	hypothetical protein
2	WP_005984209.1	inorganic phosphate transporter
3	WP_005984208.1	DUF47 family protein
4	WP_005984207.1	TRL-like family protein
5	WP_005984206.1	hypothetical protein
6	WP_005984205.1	4Fe-4S binding protein
7	WP_005984204.1	ferredoxin
8	WP_005984203.1	LysM peptidoglycan-binding domain-containing protein
9	WP_005984202.1	hypothetical protein
10	WP_005984201.1	hypothetical protein

Nº	Accesion number	Product
**11**	**WP_005984200.1**	**cytochrome c nitrite reductase small subunit**
12	WP_005984199.1	ammonia-forming cytochrome c nitrite reductase subunit c552
13	WP_005984198.1	phosphoribosyltransferase
14	WP_005984197.1	hypothetical protein
15	WP_005984196.1	YibE/F family protein
16	WP_005984195.1	alkaline phosphatase
17	WP_005984194.1	superoxide dismutase
18	WP_005984193.1	hypothetical protein
19	WP_005984192.1	PAS domain S-box protein

C

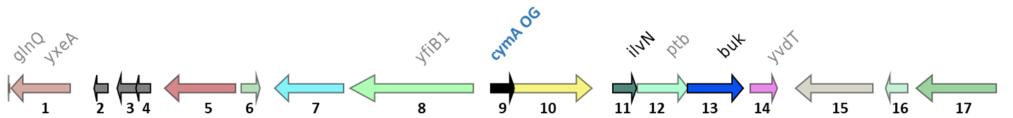

Nº	Accesion number	Product
1	WP_014524256.1	ABC transporter permease
2	WP_010937917.1	hypothetical protein
3	WP_014524257.1	hypothetical protein
4	WP_010937920.1	hypothetical protein
5	WP_010937922.1	sigma-54-dependent Fis family transcriptional regulator
6	WP_010937923.1	Rid family detoxifying hydrolase
7	WP_010937924.1	sigma-54 dependent transcriptional regulator
8	WP_010937925.1	ATP-binding protein
**9**	**WP_010937927.1**	**NapC/NirT family cytochrome c**

Nº	Accesion number	Product
10	WP_010937928.1	ammonia-forming cytochrome c nitrite reductase subunit c552
11	WP_010937929.1	acetolactate synthase small subunit (ilvN)
12	WP_010937930.1	phosphate butyryltransferase
13	WP_010937931.1	butyrate kinase (buk)
14	WP_010937932.1	TetR/AcrR family transcriptional regulator
15	WP_010937934.1	DUF4139 domain-containing protein
16	WP_014524258.1	cupin domain-containing protein
17	WP_010937936.1	PBP1A family penicillin-binding protein

D

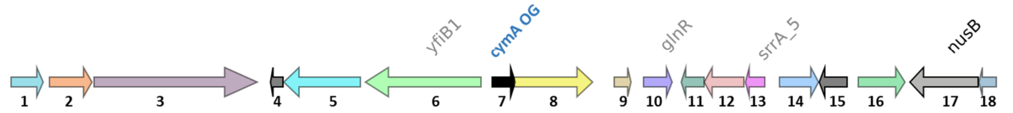

Nº	Accesion number	Product
1	WP_022659013.1	U32 family peptidase
2	WP_022659014.1	methyltransferase domain-containing protein
3	WP_027180863.1	UvrD-helicase domain-containing protein
4	WP_027180864.1	hypothetical protein
5	WP_022659016.1	sigma-54 dependent transcriptional regulator
6	WP_051135420.1	ATP-binding protein
**7**	**WP_022659018.1**	**NapC/NirT family cytochrome c**
8	pseudogene	ammonia-forming cytochrome c nitrite reductase subunit c552
9	WP_245170799.1	C40 family peptidase

Nº	Accesion number	Product
10	WP_022659020.1	MerR family transcriptional regulator
11	WP_022659021.1	GNAT family N-acetyltransferase
12	WP_027180866.1	thioredoxin domain-containing protein
13	WP_022659023.1	response regulator
14	WP_022659024.1	AAA family ATPase
15	WP_245170800.1	hypothetical protein
16	WP_022659027.1	ParB/RepB/Spo0J family partition protein
17	WP_022659028.1	antitermination factor (NusB)
18	WP_245170801.1	DUF116 domain-containing protein
