# Supplementary material for: Novel Insights on Extracellular Electron Transfer Networks in the Desulfovibrionaceae Family: Unveiling the Potential Significance of Horizontal Gene Transfer
Source: Microorganisms. 2024 Aug 29;12(9):1796. doi: 10.3390/microorganisms12091796 (PMC11434368; doi:10.3390/microorganisms12091796)
Supplement: Supplementary file 1 [file microorganisms-12-01796-s001.zip › Figures.zip!/Figure_S13_Phylogeny_genomic_context_cbc.pdf]

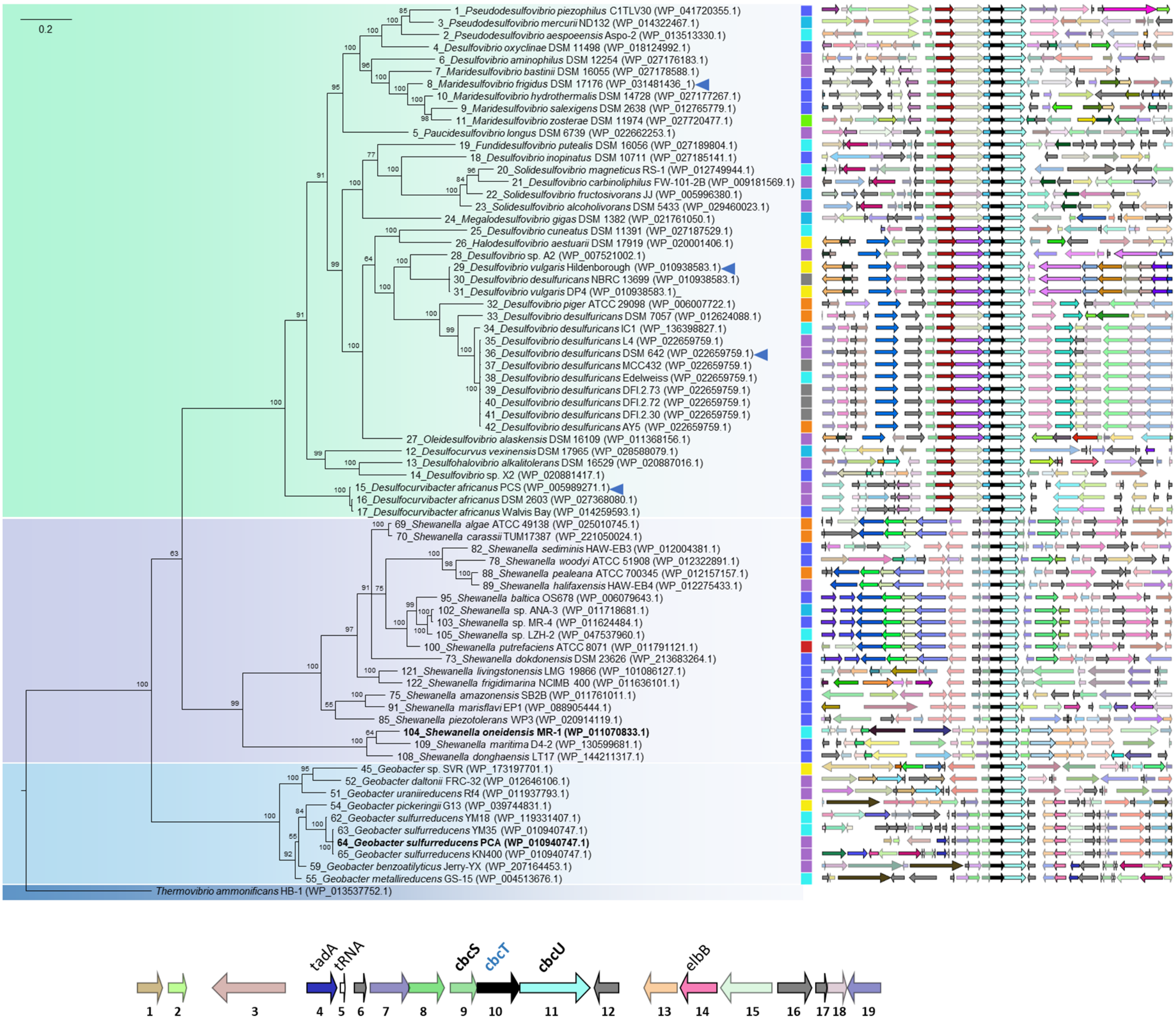

№	Accesion number	Product
1	WP_010940738.1	DUF3232 domain-containing protein
2	WP_010940739.1	type IV secretion system DNA-binding domain-containing protein
3	WP_010940741.1	site-specific integrase
4	WP_010940742.1	tRNA adenosine(34) deaminase (TadA)
5	NA	tRNA-Ser
6	WP_010940743.1	hypothetical protein
7	WP_010940744.1	peroxiredoxin-like family protein
8	WP_010940745.1	carbonic anhydrase
9	WP_010940746.1	cytochrome c (CbcS)
10	WP_010940747.1	4Fe-4S dicluster domain-containing protein (CbcT)

№	Accesion number	Product
11	WP_010940748.1	polysulfide reductase NrfD (CbcU)
12	WP_010940749.1	hypothetical protein
13	WP_010940750.1	porin family protein
14	WP_010940751.1	isoprenoid biosynthesis glyoxalase (ElbB)
15	WP_010940752.1	NAD(P)H-binding protein
16	WP_010940753.1	hypothetical protein
17	WP_010940754.1	hypothetical protein
18	WP_010940755.1	PilZ domain-containing protein
19	WP_010940756.1	XRE family transcriptional regulator
