# Supplementary material for: Novel Insights on Extracellular Electron Transfer Networks in the Desulfovibrionaceae Family: Unveiling the Potential Significance of Horizontal Gene Transfer
Source: Microorganisms. 2024 Aug 29;12(9):1796. doi: 10.3390/microorganisms12091796 (PMC11434368; doi:10.3390/microorganisms12091796)
Supplement: Supplementary file 1 [file microorganisms-12-01796-s001.zip › Figures.zip!/Figure_S14_Phylogeny_genomic_context_cbcC.pdf]

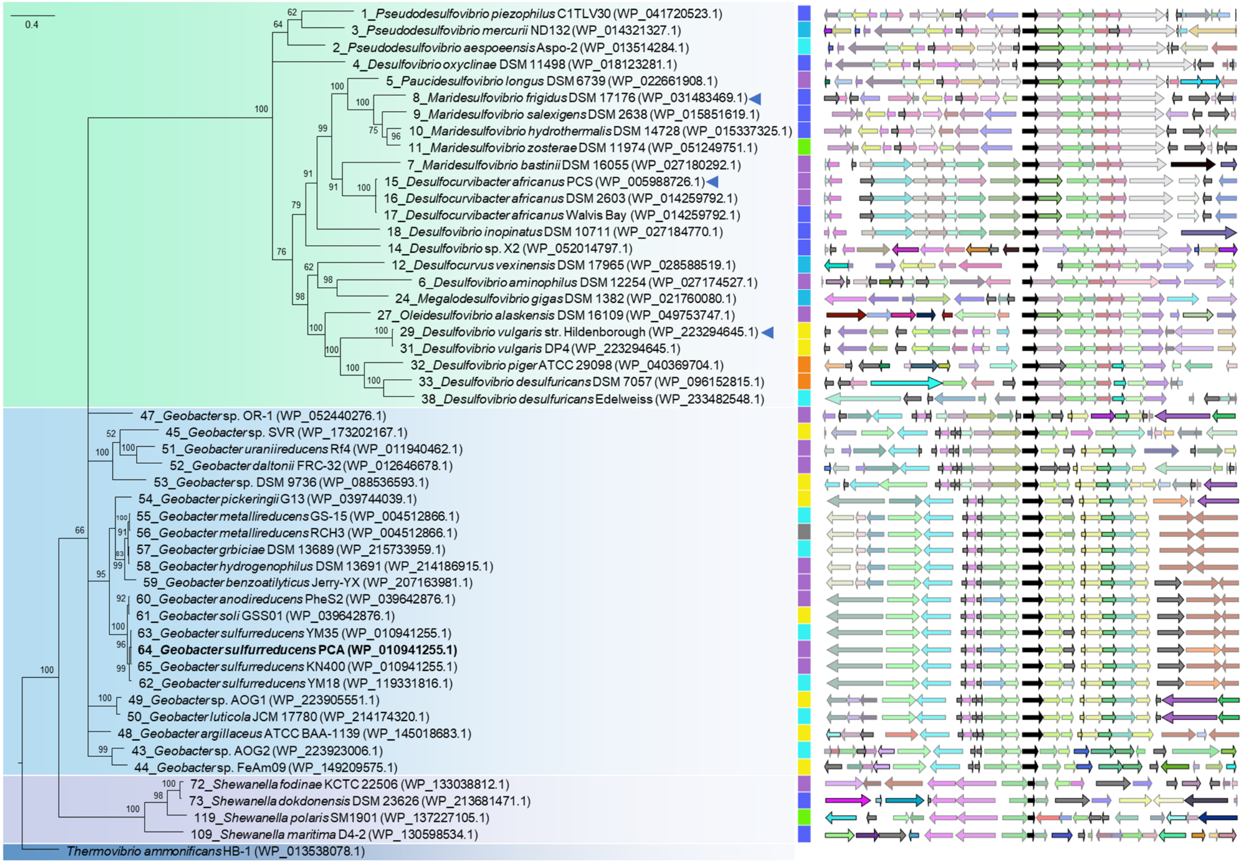

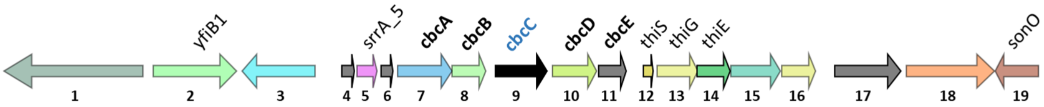

Nº	Accesion number	Product
1	WP_010941263.1	alpha-amylase family glycosyl hydrolase
2	WP_010941262.1	ATP-binding protein
3	WP_010941261.1	sigma-54 dependent transcriptional regulator
4	WP_010941260.1	hypothetical protein
5	WP_010941259.1	response regulator (srrA-5)
6	WP_010941258.1	hypothetical protein
7	WP_010941257.1	multiheme c-type cytochrome (CbcA)
8	WP_010941256.1	cytochrome b/b6 domain-containing protein (CbcB)
9	WP_010941255.1	cytochrome c3 family protein (CbcC)
10	WP_010941254.1	HAMP domain-containing protein (CbcD)

Nº	Accesion number	Product
11	WP_010941253.1	hypothetical protein (CbcE)
12	WP_010941252.1	sulfur carrier protein (ThiS)
13	WP_010941251.1	thiazole synthase (ThiG)
14	WP_010941250.1	thiamine phosphate synthase (ThiE)
15	WP_010941249.1	TIGR01212 family radical SAM protein
16	WP_010941248.1	fumarylacetoacetate hydrolase family protein
17	WP_010941247.1	hypothetical protein
18	WP_010941246.1	HAMP domain-containing methyl-accepting chemotaxis protein
19	WP_010941245.1	methyl-accepting chemotaxis protein (sonO)
