# Supplementary material for: Novel Insights on Extracellular Electron Transfer Networks in the Desulfovibrionaceae Family: Unveiling the Potential Significance of Horizontal Gene Transfer
Source: Microorganisms. 2024 Aug 29;12(9):1796. doi: 10.3390/microorganisms12091796 (PMC11434368; doi:10.3390/microorganisms12091796)
Supplement: Supplementary file 1 [file microorganisms-12-01796-s001.zip › Figures.zip!/Figure_S15_networks_by_origin.pdf]

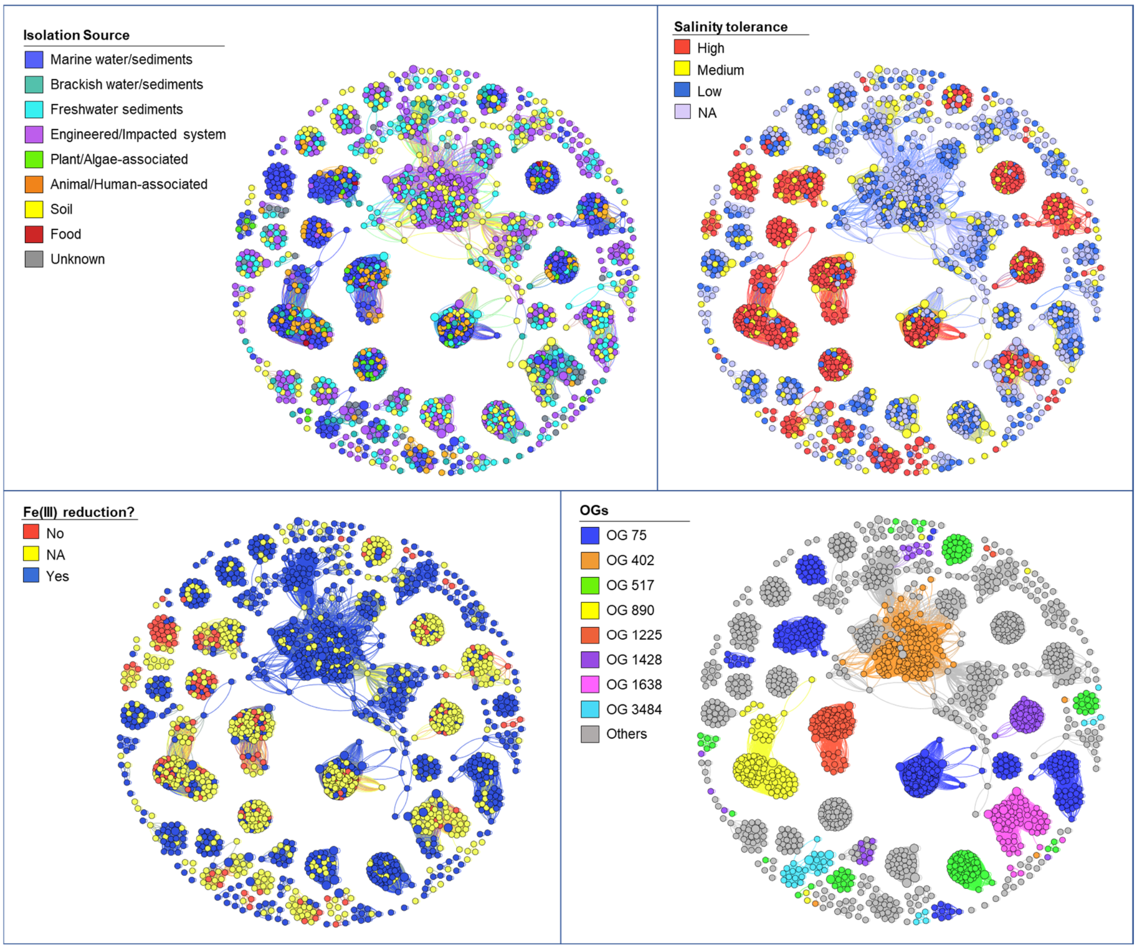
Isolation Source
Marine water/sediments
Brackish water/sediments
Freshwater sediments
Engineered/Impacted system
Plant/Algae-associated
Animal/Human-associated
Soil
Food
Unknown
Salinity tolerance
High
Medium
Low
NA
Fe(III) reduction?
No
NA
Yes
OGs
OG 75
OG 402
OG 517
OG 890
OG 1225
OG 1428
OG 1638
OG 3484
Others
