# Supplementary material for: Novel Insights on Extracellular Electron Transfer Networks in the Desulfovibrionaceae Family: Unveiling the Potential Significance of Horizontal Gene Transfer
Source: Microorganisms. 2024 Aug 29;12(9):1796. doi: 10.3390/microorganisms12091796 (PMC11434368; doi:10.3390/microorganisms12091796)
Supplement: Supplementary file 1 [file microorganisms-12-01796-s001.zip › Figures.zip!/Figure_S3_compuestaGC_genomesize.pdf]

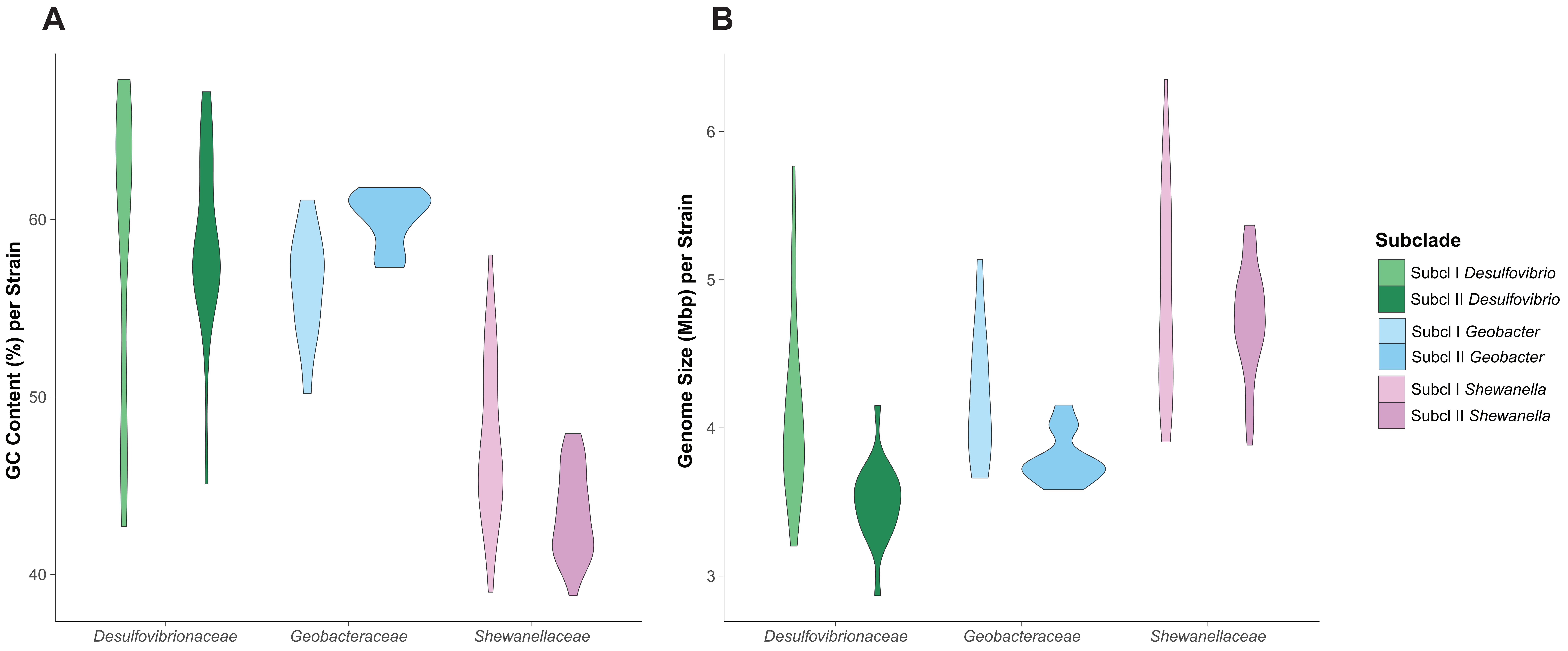

A
GC Content (%) per Strain
60
50
40
Desulfovibrionaceae
Geobacteraceae
Shewanellaceae
B
Genome Size (Mbp) per Strain
6
5
4
3
Desulfovibrionaceae
Geobacteraceae
Shewanellaceae
Subclade
Subcl I Desulfovibrio
Subcl II Desulfovibrio
Subcl I Geobacter
Subcl II Geobacter
Subcl I Shewanella
Subcl II Shewanella
