# Supplementary material for: Novel Insights on Extracellular Electron Transfer Networks in the Desulfovibrionaceae Family: Unveiling the Potential Significance of Horizontal Gene Transfer
Source: Microorganisms. 2024 Aug 29;12(9):1796. doi: 10.3390/microorganisms12091796 (PMC11434368; doi:10.3390/microorganisms12091796)
Supplement: Supplementary file 1 [file microorganisms-12-01796-s001.zip › Figures.zip!/Figure_S5_numeroprofagosVSdensidadprofagos.pdf]

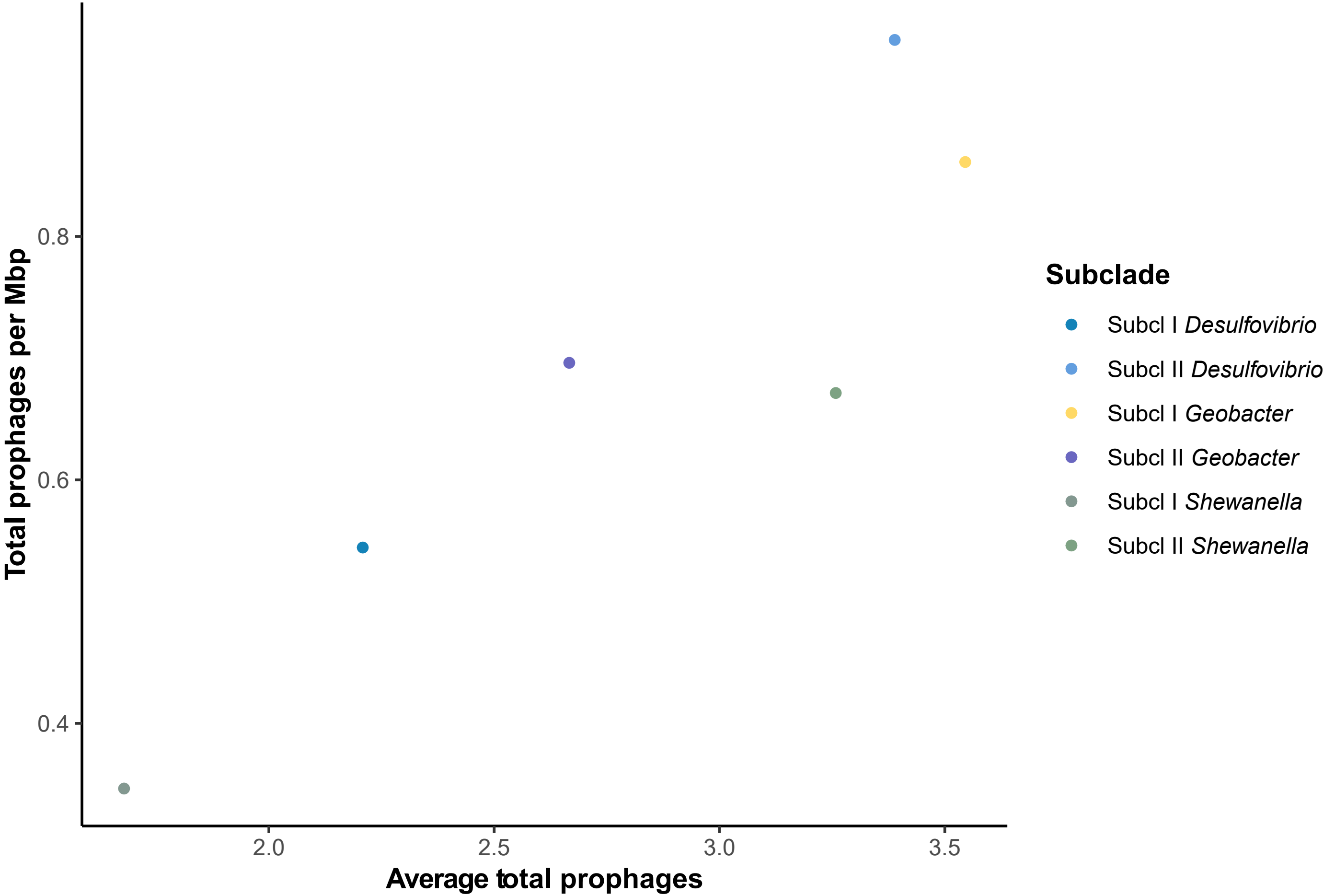

Total prophages per Mbp
0.4
0.6
0.8
2.0
2.5
3.0
3.5
Average total prophages
Subclade
Subcl I *Desulfovibrio*
Subcl II *Desulfovibrio*
Subcl I *Geobacter*
Subcl II *Geobacter*
Subcl I *Shewanella*
Subcl II *Shewanella*
