# Supplementary material for: Novel Insights on Extracellular Electron Transfer Networks in the Desulfovibrionaceae Family: Unveiling the Potential Significance of Horizontal Gene Transfer
Source: Microorganisms. 2024 Aug 29;12(9):1796. doi: 10.3390/microorganisms12091796 (PMC11434368; doi:10.3390/microorganisms12091796)
Supplement: Supplementary file 1 [file microorganisms-12-01796-s001.zip › Figures.zip!/Figure_S6_PCA_habitat.pdf]

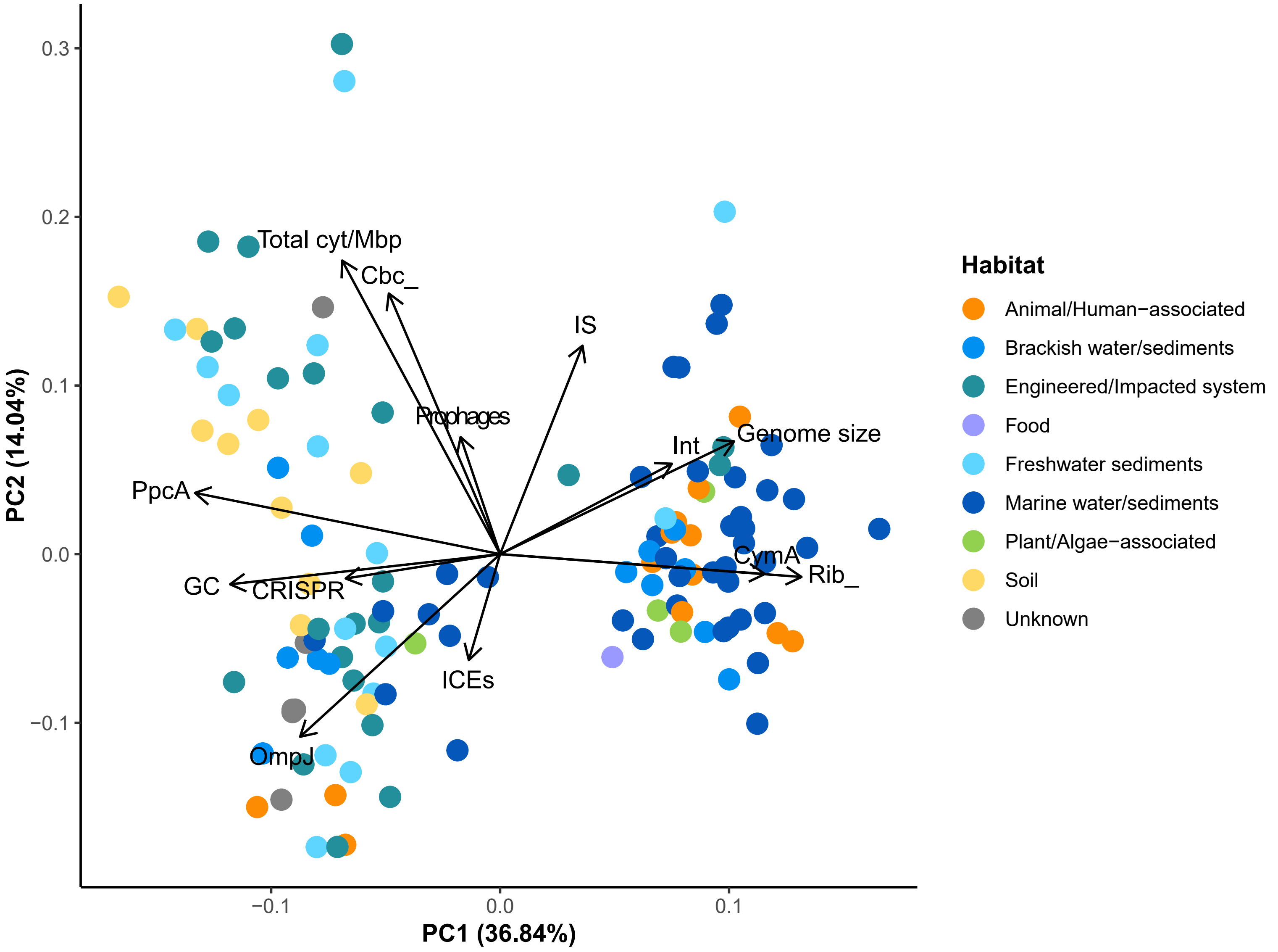

Total cyt/Mbp
Cbc_
IS
Prophages
Int
Genome size
PpcA
CymA
Rib_
GC
CRISPR
ICEs
OmpJ
0.3
0.2
0.1
0.0
−0.1
PC2 (14.04%)
−0.1
0.0
0.1
PC1 (36.84%)
Habitat
Animal/Human−associated
Brackish water/sediments
Engineered/Impacted system
Food
Freshwater sediments
Marine water/sediments
Plant/Algae−associated
Soil
Unknown
