# Supplementary material for: Novel Insights on Extracellular Electron Transfer Networks in the Desulfovibrionaceae Family: Unveiling the Potential Significance of Horizontal Gene Transfer
Source: Microorganisms. 2024 Aug 29;12(9):1796. doi: 10.3390/microorganisms12091796 (PMC11434368; doi:10.3390/microorganisms12091796)
Supplement: Supplementary file 1 [file microorganisms-12-01796-s001.zip › Figures.zip!/Figure_S7_PpcA_tree_genomic_context.pdf]

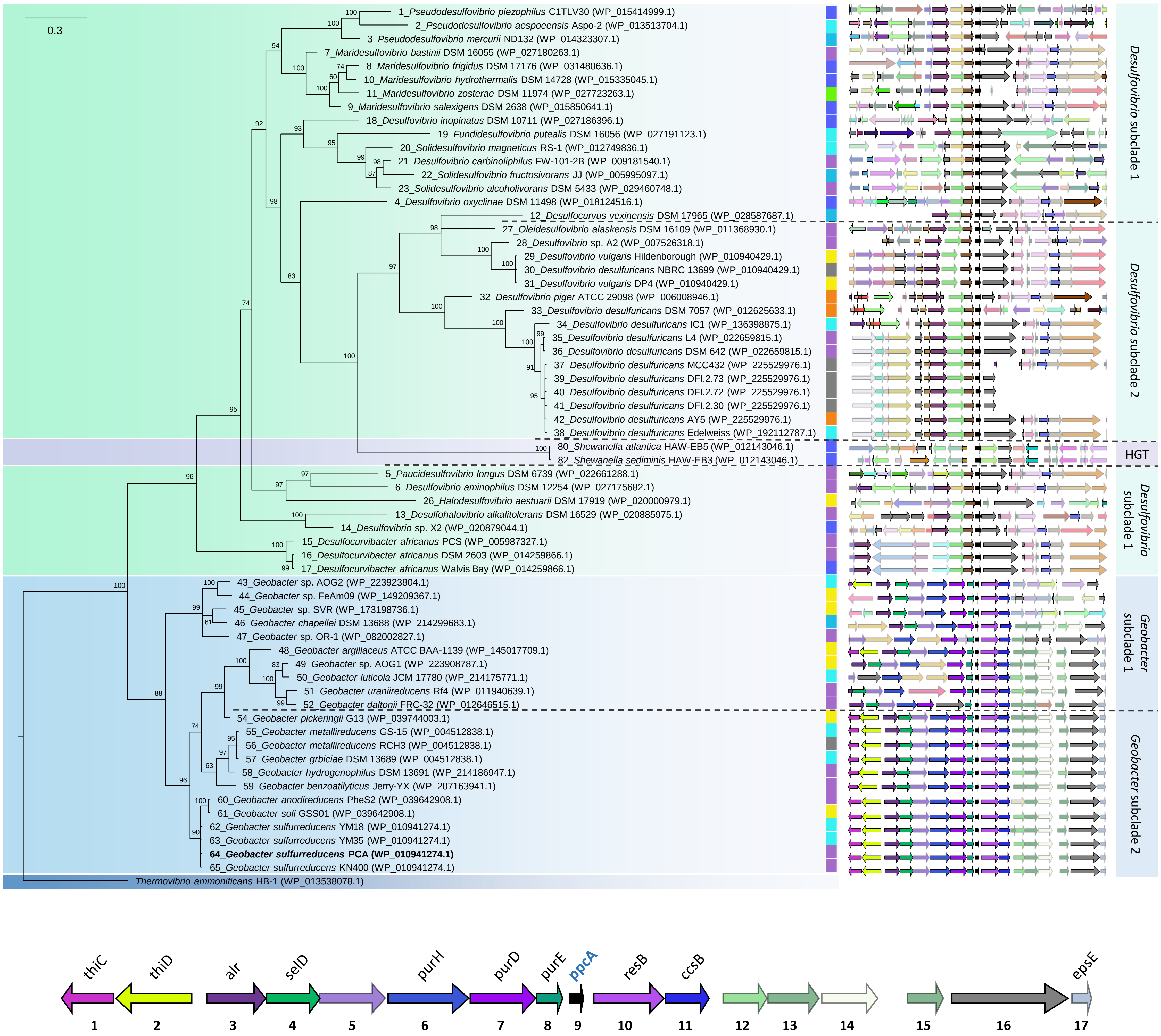

Nº	Accesion number	Product
1	WP_010941266.1	phosphomethylpyrimidine synthase (ThiC)
2	WP_010941267.1	bifunctional hydroxymethylpyrimidine (ThicD) kinase/phosphomethylpyrimidine kinase
3	WP_010941268.1	alanine racemase (alr)
4	WP_010941269.1	selenide%2C water dikinase (SelD)
5	WP_010941270.1	ASKHA domain-containing protein
6	WP_010941271.1	bifunctional phosphoribosylaminoimidazolecarboxamide formyltransferase/IMP cyclohydrolase (purH)
7	WP_010941272.1	phosphoribosylamine--glycine ligase (purD)
8	WP_010941273.1	5-(carboxyamino)imidazole ribonucleotide mutase (purE)

Nº	Accesion number	Product
9	WP_010941274.1	cytochrome c3 family protein (PpcA)
10	WP_010941275.1	cytochrome c biogenesis protein (ResB)
11	WP_010941276.1	c-type cytochrome biogenesis protein (CcsB)
12	WP_235044958.1	cytochrome C
13	WP_010941278.1	cytochrome c3 family protein
14	WP_010941279.1	6-bladed beta-propeller
15	WP_010941282.1	cytochrome c3 family protein
16	WP_161598787.1	hypothetical protein
17	WP_010941284.1	glycosyltransferase
