# Supplementary material for: Novel Insights on Extracellular Electron Transfer Networks in the Desulfovibrionaceae Family: Unveiling the Potential Significance of Horizontal Gene Transfer
Source: Microorganisms. 2024 Aug 29;12(9):1796. doi: 10.3390/microorganisms12091796 (PMC11434368; doi:10.3390/microorganisms12091796)
Supplement: Supplementary file 1 [file microorganisms-12-01796-s001.zip › Figures.zip!/Figure_S8_genomic_context_of_ppcA_other_Desulfovibrio.pdf]

A

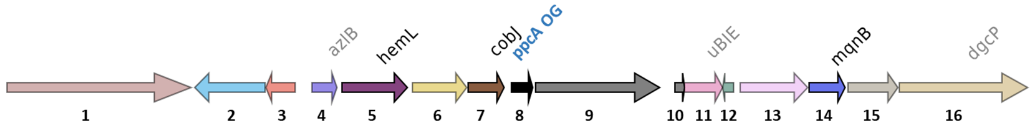

Nº	Accesion number	Product
1	WP_051676928.1	glycosyl hydrolase family 18 protein
2	WP_034602861.1	multiheme c-type cytochrome
3	WP_031480646.1	NapC/NirT family cytochrome c
4	WP_031480644.1	Lrp/AsnC family transcriptional regulator
5	WP_031480642.1	glutamate-1-semialdehyde 2%2C1-aminomutase (hemL)
6	WP_034602860.1	cobalt-precorrin 5A hydrolase
7	WP_051676927.1	precorrin-3B C(17)-methyltransferase (cobJ)
**8**	**WP_031480636.1**	**cytochrome c3 family protein**

Nº	Accesion number	Product
9	WP_031480634.1	hypothetical protein
10	WP_031480632.1	hypothetical protein
11	WP_031480630.1	ubiquinone/menaquinone biosynthesis methyltransferase (uBIE)
12	WP_031480628.1	DUF2065 domain-containing protein
13	WP_031480626.1	nucleotide sugar dehydrogenase
14	WP_031480623.1	futalosine hydrolase (mqnB)
15	WP_031480621.1	polyprenyl synthetase family protein
16	WP_031480619.1	diguanylate cyclase
17	WP_031480617.1	AIR synthase-related protein (dgcP)

B

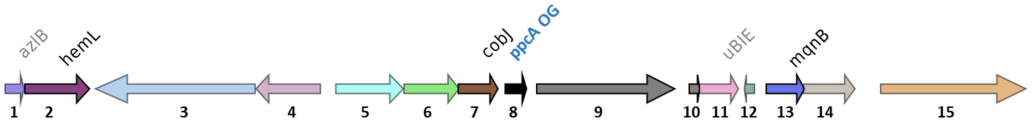

Nº	Accesion number	Product
1	WP_005987314.1	Lrp/AsnC family transcriptional regulator
2	WP_005987316.1	glutamate-1-semialdehyde 2%2C1-aminomutase (hemL)
3	WP_005987318.1	multidrug efflux RND transporter permease subunit
4	WP_005987320.1	efflux RND transporter periplasmic adaptor subunit
5	WP_005987322.1	hemolysin family protein
6	WP_005987324.1	cobalamin biosynthesis protein
7	WP_005987325.1	precorrin-3B C(17)-methyltransferase (cobJ)
**8**	**WP_005987327.1**	**cytochrome c3 family protein**

Nº	Accesion number	Product
9	WP_005987328.1	hypothetical protein
10	WP_005987330.1	hypothetical protein
11	WP_043806131.1	ubiquinone/menaquinone biosynthesis methyltransferase
12	WP_005987335.1	DUF2065 domain-containing protein
13	WP_005987337.1	futalosine hydrolase (mqnB)
14	WP_005987340.1	polyprenyl synthetase family protein
15	WP_005987342.1	AIR synthase-related protein

C

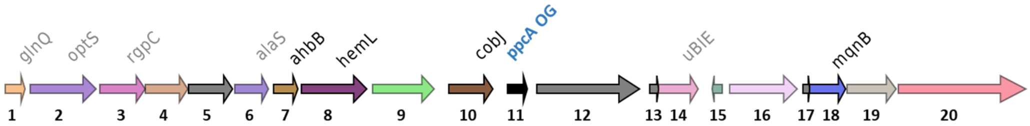

Nº	Accesion number	Product
1	WP_010940419.1	ABC transporter ATP-binding protein
2	WP_010940420.1	ABC transporter substrate-binding protein
3	WP_010940421.1	sugar ABC transporter permease
4	WP_010940422.1	carbohydrate ABC transporter permease
5	WP_010940423.1	hypothetical protein
6	WP_010940424.1	alanyl-tRNA synthetase
7	WP_010940425.1	siroheme decarboxylase subunit beta (ahbB)
8	WP_010940426.1	glutamate-1-semialdehyde 2%2C1-aminomutase (hemL)
9	WP_010940427.1	cobalamin biosynthesis protein
10	WP_010940428.1	precorrin-3B C(17)-methyltransferase (cobJ)

Nº	Accesion number	Product
**11**	**WP_010940429.1**	**cytochrome c3 family protein**
12	WP_014524624.1	hypothetical protein
13	WP_010940431.1	hypothetical protein
14	WP_010940432.1	ubiquinone/menaquinone biosynthesis methyltransferase
15	WP_010940433.1	DUF2065 domain-containing protein
16	WP_010940434.1	nucleotide sugar dehydrogenase
17	WP_014524625.1	hypothetical protein
18	WP_014524626.1	futalosine hydrolase (mqnB)
19	WP_010940437.1	polyprenyl synthetase family protein
20	WP_010940438.1	HDOD domain-containing protein

D

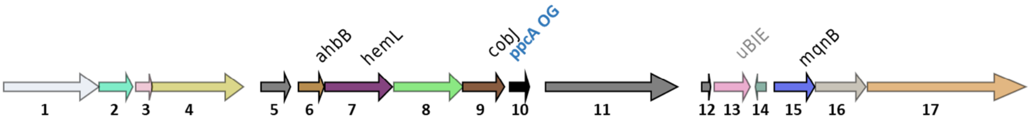

Nº	Accesion number	Product
1	WP_022659824.1	DUF294 nucleotidyltransferase-like domain-containing protein
2	WP_022659823.1	3'-5' exonuclease
3	WP_022659822.1	DUF485 domain-containing protein
4	WP_022659821.1	cation acetate symporter
5	WP_022659820.1	hypothetical protein
6	WP_022659819.1	siroheme decarboxylase subunit beta (ahbB)
7	WP_022659818.1	glutamate-1-semialdehyde 2%2C1-aminomutase (hemL)
8	WP_022659817.1	cobalamin biosynthesis protein

Nº	Accesion number	Product
9	WP_022659816.1	precorrin-3B C(17)-methyltransferase (cobJ)
**10**	**WP_022659815.1**	**cytochrome c3 family protein**
11	WP_022659814.1	hypothetical protein
12	WP_022659812.1	hypothetical protein
13	WP_108702591.1	ubiquinone/menaquinone biosynthesis methyltransferase
14	WP_022659810.1	DUF2065 domain-containing protein
15	WP_022659808.1	futalosine hydrolase (mqnB)
16	WP_022659807.1	polyprenyl synthetase family protein
17	WP_022659806.1	AIR synthase-related protein
