# Supplementary material for: Novel Insights on Extracellular Electron Transfer Networks in the Desulfovibrionaceae Family: Unveiling the Potential Significance of Horizontal Gene Transfer
Source: Microorganisms. 2024 Aug 29;12(9):1796. doi: 10.3390/microorganisms12091796 (PMC11434368; doi:10.3390/microorganisms12091796)
Supplement: Supplementary file 1 [file microorganisms-12-01796-s001.zip › Figures.zip!/Figure_S9_Phylogeny_ompJ_cymA_cbc.pdf]

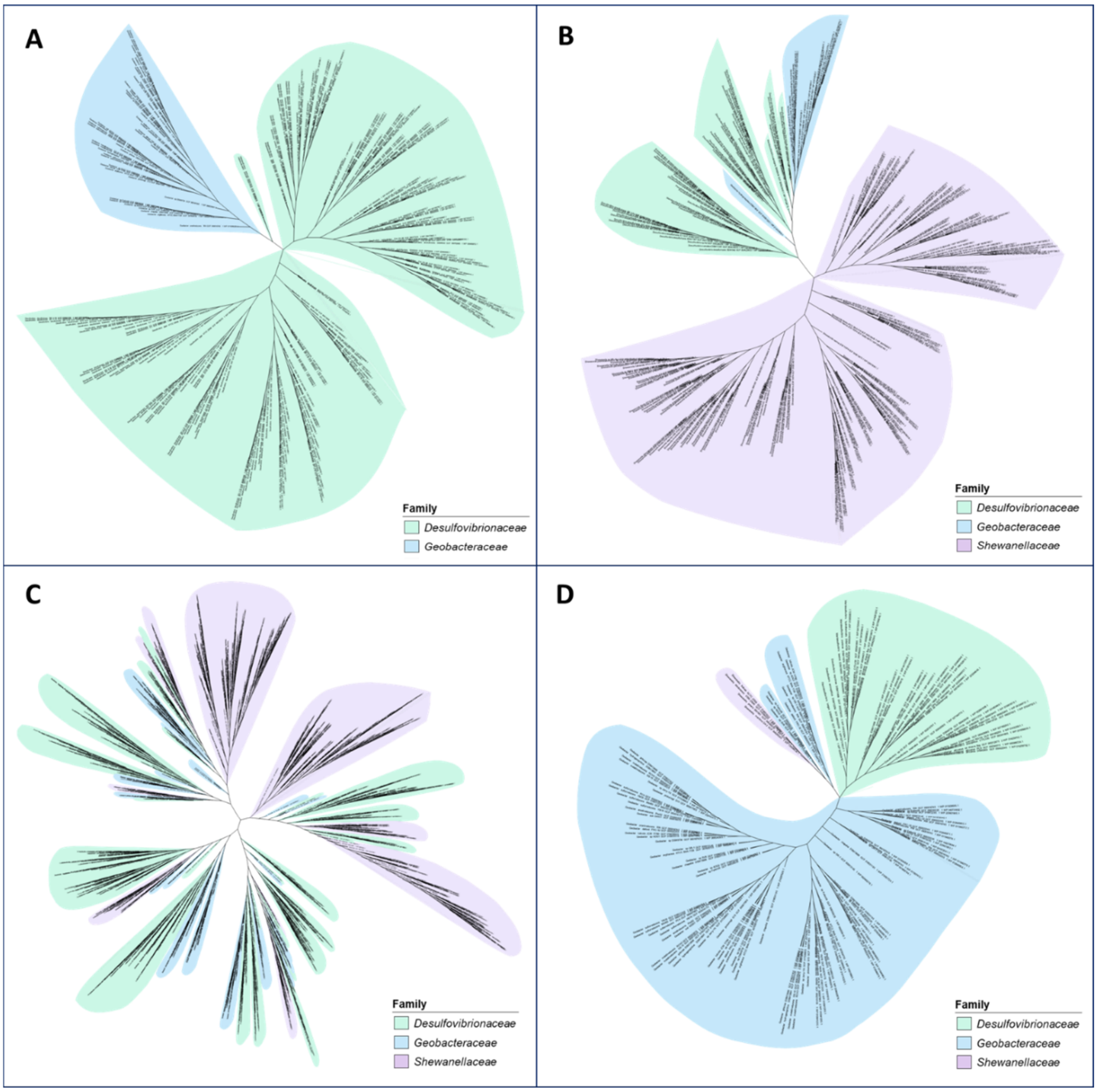
A
Family
Desulfovibrionaceae
Geobacteraceae
B
Family
Desulfovibrionaceae
Geobacteraceae
Shewanellaceae
C
Family
Desulfovibrionaceae
Geobacteraceae
Shewanellaceae
D
Family
Desulfovibrionaceae
Geobacteraceae
Shewanellaceae
