# Supplementary material for: Novel Insights on Extracellular Electron Transfer Networks in the Desulfovibrionaceae Family: Unveiling the Potential Significance of Horizontal Gene Transfer
Source: Microorganisms. 2024 Aug 29;12(9):1796. doi: 10.3390/microorganisms12091796 (PMC11434368; doi:10.3390/microorganisms12091796)
Supplement: Supplementary file 1 [file microorganisms-12-01796-s001.zip › Figures.zip!/Supplementary_Figures_Headings.pdf]

Figure S1. Orthology analysis of the SRB and FeRB genomes. (A) Number of orthogroups (OGs) versus the number of genomes in log10 scale. The first (left) and last (right) bars represent the species-specific OGs (546) and the core OGs with all species present (289), respectively. (D) Venn diagram showing unique and shared ortholog gene clusters in the three families. The number of unassigned genes to OGs is shown in parentheses.

Figure S2: Phylogenomic tree of the analyzed genomes belonging to the *Desulfovibrionaceae*, *Geobacteraceae,* and *Shewanellaceae* families. Phylogeny was inferred using Orthofinder v2.5.4, identifying 109 single-copy orthogroups with all species present. (A) Clades of each family, (B), (C), and (D) show the subclades of strains belonging to *Desulfovibrionaceae*, *Geobacteraceae,* and *Shewanellaceae* families, respectively.

Figure S3: Violin-plots of the distribution of (A) genomic GC content and (B) genome size by subclades of *Desulfovibrionaceae*, *Geobacteraceae,* and *Shewanellaceae* families.

Figure S4. Presence/absence matrix of genes of complementary genes related to EET mechanisms of *S. oneidensis* MR-1 y *G. sulfurreducens* PCA. On the left, the phylogenomic cladogram is presented with the isolation source according to Figure 1.

Figure S5: Correlation between prophage density (total number of prophages per Mbp) and the average total prophages per strain found in each family.

Figure S6: Principal Component Analysis of the strains from the *Desulfovibrionaceae*, *Geobacteraceae* and *Shewanellaceae* families in relation to their mobilome components, types of cytochromes, genome size, and GC content. Colors indicate strains origin/habitat.

Figure S7. Phylogeny and genetic context of PpcA orthologous proteins. The phylogenetic tree was constructed using MrBayes method based on amino acid sequences of 64 proteins, 40 belonging to members of *Desulfovibrionaceae* family, 22 belonging to *Geobacteraceae* family and two belonging to *Shewanellaceae* family. Bayesian posterior probabilities are indicated above their branch. The scale bar shows 0.3 estimated substitutions per site. The tree was rooted to the closest protein of *Thermovibrio ammonificans* HB-1. The number before the bacterial name indicates the position with respect to the phylogenomic tree (obtained with Orthofinder). Colors next to the phylogenetic tree indicate the source of isolation: blue for marine waters/sediments, turquoise for brackish water/sediments, cyan for freshwater sediments, purple for engineered/impacted system, green for plant/algae-associated, orange for animal/ human-associated, yellow for soil, red for food, and gray for unknown source. The blue triangles indicate those bacteria from *Desulfovibrionaceae* family that have reported Fe(III) reduction. On the right, the genetic contexts of the *ppcA* orthologous genes are visualized. The lower zone shows the genetic context of the gene encoding the periplasmic cytochrome ppcA of *Geobacter sulfurreducens* PCA.

Figure S8. Genetic context of *ppcA*-orthologous genes in others representative strains of the *Desulfovibrionaceae* family. Genetic context of *ppcA* gene of **A)** *Maridesulfovibrio frigidus* DSM 17176 (WP_031480636.1), **B)** *Desulfocurvibacter africanus* PCS (WP_005987327.1), **C)** *Desulfovibrio vulgaris* Hildenborough (WP_010940429.1), and **D)** *Desulfovibrio desulfuricans* DSM 642 (WP_022659815.1).

Figure S9. Phylogenetic trees of the orthologous groups associated with OmpJ, CymA, CbcT and CbcC. The phylogenetic trees were inferred using Orthofinder v2.5.4. A) Phylogenetic tree of proteins homologous of the outer membrane protein OmpJ. The tree comprises 225 protein sequences: 42 from *Geobacter* strains and 183 from members of the *Desulfovibrionaceae* family. B) Phylogenetic tree of proteins homologous of the c-type cytochrome CymA. The tree is composed of 232 protein sequences: 17 from *Geobacter* strains, 162 from *Shewanella* and 53 from members of the *Desulfovibrionaceae* family. C) Phylogenetic tree of proteins homologous of the cytochrome bc complex CbcT. The tree is composed of 1018 protein sequences: 161 from *Geobacter* strains, 447 from *Shewanella* and 410 from members of the *Desulfovibrionaceae* family. D) Phylogenetic tree of proteins homologous of the cytochrome bc complex CbcC. The tree is composed of 136 protein sequences: 94 from *Geobacter* strains, 4 from *Shewanella* and 38 from members of the *Desulfovibrionaceae* family. Each clade/branch is colored according to its family of origin.

Figure S10. Phylogeny and genetic context of OmpJ orthologous proteins. The phylogenetic tree was constructed using MrBayes method based on amino acid sequences of 64 proteins, 42 belonging to members of *Desulfovibrionaceae* family and 22 belonging to *Geobacteraceae* family. Bayesian posterior probabilities are indicated above their branch. The scale bar shows 0.3 estimated substitutions per site. The tree was rooted to the closest protein of *Thermovibrio ammonificans* HB-1. The number before the bacterial name indicates the position with respect to the phylogenomic tree (obtained with Orthofinder). Colors next to the phylogenetic tree indicate the source of isolation: blue for marine waters/sediments, turquoise for brackish water/sediments, cyan for freshwater sediments, purple for engineered/impacted system, green for plant/algae-associated, orange for animal/ human-associated, yellow for soil, red for food, and gray for unknown source. The blue triangles indicate those bacteria from *Desulfovibrionaceae* family that have reported Fe(III) reduction. On the right, the genetic contexts of the *ompJ* orthologous genes are visualized. The bottom of the figure shows the genetic context of the gene encoding the outer membrane protein OmpJ of *Geobacter sulfurreducens* PCA.

Figure S11. Phylogeny and genetic context of CymA orthologous proteins. The phylogenetic tree was constructed using MrBayes method based on amino acid sequences of 113 proteins, 40 belonging to members of *Desulfovibrionaceae* family, 14 to *Geobacteraceae* family and 59 belonging to *Shewanellaceae* family. Bayesian posterior probabilities are indicated above their branch. The scale bar shows 0.3 estimated substitutions per site. The tree was rooted to the closest protein of *Thermovibrio ammonificans* HB-1. The number before the bacterial name indicates the position with respect to the phylogenomic tree (obtained with Orthofinder). Colors next to the phylogenetic tree indicate the source of isolation: blue for marine waters/sediments, turquoise for brackish water/sediments, cyan for freshwater sediments, purple for engineered/impacted system, green for

plant/algae-associated, orange for animal/ human-associated, yellow for soil, red for food, and gray for unknown source. The blue triangles indicate those bacteria from *Desulfovibrionaceae* family that have reported Fe(III) reduction. On the right, the genetic contexts of the *cymA* orthologous genes are visualized. The lower zone shows the genetic context of the gene encoding the tetraheme c-type cytochrome CymA of *Shewanella oneidensis MR-1.*

Figure S12. Genetic context of *cymA*-orthologous genes in others representative strains of the *Desulfovibrionaceae* family. Genetic context of *cymA* gene of **A)** *Maridesulfovibrio frigidus* DSM 17176 (WP_031480646.1), **B)** *Desulfocurvibacter africanus* PCS (WP_005984200.1), **C)** *Desulfovibrio vulgaris* Hildenborough (WP_010937927.1), and **D)** *Desulfovibrio desulfuricans* DSM 642 (WP_022659018.1).

Figure S13. Phylogeny and genetic context of CbcT orthologous proteins. The phylogenetic tree was constructed using MrBayes method based on amino acid sequences of 72 proteins, 42 belonging to members of *Desulfovibrionaceae* family, and 30 representative sequences of the *Geobacteraceae* and *Shewanellaceae* families (10 and 20, respectively). Bayesian posterior probabilities are indicated above their branch. The scale bar shows 0.2 estimated substitutions per site. The tree was rooted to the closest protein of *Thermovibrio ammonificans* HB-1. The number before the bacterial name indicates the position with respect to the phylogenomic tree (obtained with Orthofinder). Colors next to the phylogenetic tree indicate the source of isolation: blue for marine waters/sediments, turquoise for brackish water/sediments, cyan for freshwater sediments, purple for engineered/impacted system, green for plant/algae-associated, orange for animal/ human-associated, yellow for soil, red for food, and gray for unknown source. The blue triangles indicate those bacteria from *Desulfovibrionaceae* family that have reported Fe(III) reduction. On the right, the genetic contexts of the *cbcT* orthologous genes are visualized. The lower zone shows the genetic context of the gene encoding the iron-sulfur cluster-binding protein CbcT of *Geobacter sulfurreducens* PCA. CbcT, together with c-type cytochrome CbcS and b-type cytochrome CbcU represent the menaquinol:ferricytochrome c oxidoreductase complex.

Figure S14. Phylogeny and genetic context of CbcC orthologous proteins. The phylogenetic tree was constructed using MrBayes method based on amino acid sequences of 50 proteins, 24 belonging to members of *Desulfovibrionaceae* family, 22 to *Geobacteraceae* family and 4 belonging to *Shewanellaceae* family. Bayesian posterior probabilities are indicated above their branch. The scale bar shows 0.4 estimated substitutions per site. The tree was rooted to the closest protein of *Thermovibrio ammonificans* HB-1. The number before the bacterial name indicates the position with respect to the phylogenomic tree (obtained with Orthofinder). Colors next to the phylogenetic tree indicate the source of isolation: blue for marine waters/sediments, turquoise for brackish water/sediments, cyan for freshwater sediments, purple for engineered/impacted system, green for plant/algae-associated, orange for animal/ human-associated, yellow for soil, red for food, and gray for unknown source. The blue triangles indicate those bacteria from *Desulfovibrionaceae* family that have reported Fe(III) reduction. On the right, the genetic contexts of the *cbcC* orthologous genes are visualized. The lower zone shows the genetic context of the gene encoding the c-type cytochrome

CbcC of *Geobacter sulfurreducens* PCA. CbcC is part of the Cbc5 complex (cbcEDCBA), a menaquinol:ferricytochrome c oxidoreductase complex, expressed during the reduction of Fe(III) oxide minerals.

Figure S15. Multi-heme cytochrome similarity networking. Similarity network colored according to: **A)** Isolation source of the bacteria of origin; **B)** Salt tolerance level of the bacteria; **C)** Experimental evidence of Fe(III) reduction of the bacteria and; **D)** Classification according to OGs, in color are shown some more abundant OGs (8/35).
