# Supplementary material for: Novel Insights on Extracellular Electron Transfer Networks in the Desulfovibrionaceae Family: Unveiling the Potential Significance of Horizontal Gene Transfer
Source: Microorganisms. 2024 Aug 29;12(9):1796. doi: 10.3390/microorganisms12091796 (PMC11434368; doi:10.3390/microorganisms12091796)
Supplement: Supplementary file 1 [file microorganisms-12-01796-s001.zip › Tables.zip!/Supplementary_Tables_Headings.pdf]

Table S1. Genomic features of the SRB and FeRB genomes.

Table S2: Custom database for EET-related genes belonging to the *Desulfovibrionaceae*, *Geobacteraceae,* and *Shewanellaceae* families.

Table S3: Growth conditions of SRB and FeRB genomes.

Table S4. Mobilome-related counts found in the genomes of SRB and FeRB genomes.

Table S5: Prevalence of mobilome elements in the genomes of SRB and FeRB genomes.

Table S6: Counts of *c*-type cytochromes found in the genomes of SRB and FeRB genomes.
