# Supplementary material for: Novel Insights on Extracellular Electron Transfer Networks in the Desulfovibrionaceae Family: Unveiling the Potential Significance of Horizontal Gene Transfer
Source: Microorganisms. 2024 Aug 29;12(9):1796. doi: 10.3390/microorganisms12091796 (PMC11434368; doi:10.3390/microorganisms12091796)
Supplement: Supplementary file 1 [file microorganisms-12-01796-s001.zip › Tables.zip!/Table_S7_References_in_Supplemetary.pdf]

| No | Strain | References |
|---|---|---|
| 1 | *Desulfovibrio vulgaris* str. Hildenborough (DSM 644, ATCC 29579) | [1-3] |
| 2 | *Desulfocurvibacter africanus* subsp. *africanus* DSM 2603 | [1,4] |
| 3 | *Halodesulfovibrio aestuarii* DSM 17919 | [1,5] |
| 4 | *Desulfocurvibacter africanus* subsp. *africanus* str. Walvis Bay | [1,4] |
| 5 | *Pseudodesulfovibrio aespoeensis* Aspo-2 | [1,6] |
| 7 | *Solidesulfovibrio magneticus* RS-1 | [7] |
| 8 | *Megalodesulfovibrio gigas* DSM 1382 | [1,8] |
| 9 | *Desulfovibrio piger* ATCC 29098 | [1,9] |
| 10 | *Desulfovibrio oxyclinae* DSM 11498 | [1,10] |
| 11 | *Desulfovibrio cuneatus* DSM 11391 | [1,11] |
| 12 | *Desulfocurvus vexinensis* DSM 17965 | [12] |
| 13 | *Desulfohalovibrio alkalitolerans* DSM 16529 | [13] |

| | | |
|---|---|---|
| 14 | *Solidesulfovibrio fructosivorans* JJ | [1,14] |
| 15 | *Maridesulfovibrio salexigens* DSM 2638 | [1,5] |
| 16 | *Maridesulfovibrio frigidus* DSM 17176 | [15] |
| 17 | *Maridesulfovibrio hydrothermalis* DSM 14728 | [16] |
| 18 | *Desulfovibrio* sp. X2 | [17] |
| 19 | *Desulfovibrio* sp. A2 | [18,19] |
| 20 | *Desulfovibrio aminophilus* DSM 12254 | [1,20] |
| 21 | *Pseudodesulfovibrio piezophilus* C1TLV30 | [21] |
| 22 | *Oleidesulfovibrio alaskensis* DSM 16109 | [22] |
| 23 | *Desulfovibrio carbinoliphilus* subsp. *oakridgensis* FW-101-2B | [1,23,24] |
| 24 | *Maridesulfovibrio bastinii DSM* 16055 | [25] |
| 25 | *Solidesulfovibrio alcoholivorans* DSM 5433 | [1,26] |
| 26 | *Desulfovibrio desulfuricans* DSM 642 | [1,27] |
| 27 | *Maridesulfovibrio zosterae* DSM 11974 | [1,28] |
| 28 | *Fundidesulfovibrio putealis* DSM 16056 | [29] |
| 29 | *Desulfovibrio inopinatus* DSM 10711 | [1,30] |

| | | |
|---|---|---|
| 30 | *Paucidesulfovibrio longus* DSM 6739 | [1,31] |
| 31 | *Pseudodesulfovibrio mercurii* ND132 | [32,33] |
| 32 | *Desulfocurvibacter africanus* PCS | [1,34] |
| 33 | *Desulfovibrio vulgaris* DP4 | [35] |
| 34 | *Desulfovibrio desulfuricans* MCC432 | [1] |
| 35 | *Desulfovibrio desulfuricans* L4 | [1,36] |
| 36 | *Desulfovibrio desulfuricans* Edelweiss | [1,37] |
| 37 | *Desulfovibrio desulfuricans* DFI.2.73 | [1] |
| 38 | *Desulfovibrio desulfuricans* DFI.2.72 | [1] |
| 39 | *Desulfovibrio desulfuricans* DFI.2.30 | [1] |
| 40 | *Desulfovibrio desulfuricans* NBRC 13699 (ATCC 7757=NCIB 8372) | [1,38] |
| 41 | *Desulfovibrio desulfuricans* IC1 | [1,39,40] |
| 42 | *Desulfovibrio desulfuricans* DSM 7057 (strain G11) | [1,41,42] |
| 43 | *Desulfovibrio desulfuricans* AY5 | [1,43] |

| | | |
|---|---|---|
| 44 | *Geobacter anodireducens* PheS2 | [44] |
| 45 | *Geobacter argillaceus* ATCC BAA-1139 | [45] |
| 46 | *Geobacter chapellei* DSM 13688 | [46] |
| 47 | *Geobacter daltonii* FRC-32 | [47] |
| 48 | *Geobacter grbiciae* DSM 13689 | [46] |
| 49 | *Geobacter hydrogenophilus* DSM 13691 | [46] |
| 50 | *Geobacter luticola* JCM 17780 | [48] |
| 51 | *Geobacter metallireducens* GS-15 | [49-51] |
| 52 | *Geobacter metallireducens* RCH3 | |
| 53 | *Geobacter pickeringii* G13 | [45,52] |
| 54 | *Geobacter soli* GSS01 | [53] |
| 55 | *Geobacter sp.* AOG1 | [54,55] |
| 56 | *Geobacter sp.* AOG2 | [54] |
| 57 | *Geobacter sp*. DSM 9736 | |
| 58 | *Geobacter sp.* FeAm09 | [56] |
| 59 | *Geobacter benzoatilyticus* Jerry-YX | [57] |
| 60 | *Geobacter sp.* OR-1 | [58,59] |
| 61 | *Geobacter sp*. SVR | [60-62] |

| | | |
|---|---|---|
| 62 | *Geobacter sulfurreducens* KN400 | [63,64] |
| 63 | *Geobacter sulfurreducens* PCA | [65,66] |
| 64 | *Geobacter sulfurreducens* YM18 | [67,68] |
| 65 | *Geobacter sulfurreducens* YM35 | [69] |
| 66 | *Geobacter uraniireducens* Rf4 | [70] |
| 67 | *Shewanella putrefaciens* ATCC 8071 | [71] |
| 68 | *Shewanella algae* ATCC 49138 | |
| 69 | *Shewanella algicola* JCM 31091 | [72] |
| 70 | *Shewanella carassii* TUM17387 | [73] |
| 71 | *Shewanella gelidii* JCM 30804 | [74] |
| 72 | *Shewanella glacialipiscicola* T147 | [75] |
| 73 | *Shewanella hafniensis* ATCC BAA-1207 | [76] |
| 74 | *Shewanella khirikhana* TH2012 | [77,78] |
| 75 | *Shewanella morhuae* ATCC BAA-1205 | [76] |
| 76 | *Shewanella pealeana* ATCC 700345 | [79] |
| 77 | *Shewanella polaris* SM1901 | [80] |
| 78 | *Shewanella intestini* MCCC 1A01895 | [81] |

| | | |
|---|---|---|
| 80 | *Shewanella sairae* JCM 11563 | [82] |
| 81 | *Shewanella schlegeliana* JCM 11561 | [82] |
| 82 | *Shewanella* sp. MR-4 | [83] |
| 83 | *Shewanella* sp. TC10 | [84,85] |
| 84 | *Shewanella baltica* OS678 | [86,87] |
| 85 | *Shewanella denitrificans* OS217 | [88] |
| 86 | *Shewanella dokdonensis* DSM 23626 | [89] |
| 87 | *Shewanella frigidimarina* NCIMB 400 | [90,91] |
| 88 | *Shewanella japonica* KCTC 22435 (=ATCC: BAA-316) | [92-94] |
| 89 | *Shewanella litoralis* JCM 32306 | [95] |
| 90 | *Shewanella livingstonensis* LMG 19866 | [96] |
| 91 | *Shewanella marina* JCM 15074 | [97] |
| 92 | *Shewanella maritima* D4-2 | [98] |
| 93 | *Shewanella saliphila* JCM 32304 | [95] |
| 94 | *Shewanella ulleungensis* JCM 32305 | [95] |
| 95 | *Shewanella woodyi* ATCC 51908 | [99] |
| 96 | *Shewanella aestuarii* JCM 17801 | [100] |
| 97 | *Shewanella algidipiscicola* H1 | [101] |
| 98 | *Shewanella amazonensis* SB2B | [102] |

| | | |
|---|---|---|
| 99 | *Shewanella atlantica* HAW-EB5 | [103] |
| 100 | *Shewanella canadensis* HAW-EB2 | [103] |
| 101 | *Shewanella chilikensis* JC5 | [104] |
| 102 | *Shewanella donghaensis* LT17 | [105] |
| 103 | *Shewanella fidelis* ATCC BAA-318 | [106] |
| 104 | *Shewanella indica* KJW27 | [107] |
| 105 | *Shewanella inventionis* CGMCC 1.15339 | [108] |
| 106 | *Shewanella litorisediminis* SMK1-12 | [109] |
| 107 | *Shewanella mangrovi* YQH10 | [110] |
| 108 | *Shewanella marisflavi* EP1 | [111,112] |
| 109 | *Shewanella oneidensis* MR-1 | [113,114] |
| 110 | *Shewanella piezotolerans* WP3 | [115,116] |
| 111 | *Shewanella psychrophila* WP2 | [115,117] |
| 112 | *Shewanella sediminis* HAW-EB3 | [118] |
| 113 | *Shewanella* sp. Arc9-LZ | [119] |
| 114 | *Shewanella* sp. M16 | [120] |
| 115 | *Shewanella* sp. W3-18-1 | [121,122] |
| 116 | *Shewanella vesiculosa* LMG 24424 | [123] |

| | | |
|---|---|---|
| 117 | *Shewanella violacea* DSS12 | [124-126] |
| 118 | *Shewanella hanedai* JCM 20706 | [127] |
| 119 | *Shewanella* sp. HN-41 | [128,129] |
| 120 | *Shewanella halifaxensis* HAW-EB4 | [130] |
| 121 | *Shewanella* sp. LZH-2 | [131,132] |
| 122 | *Shewanella* sp. BF02_Schw | [133] |
| 123 | *Shewanella fodinae* KCTC 22506 | [134] |
| 124 | *Shewanella* sp. ANA-3 | [135-137] |
| 125 | *Shewanella glacialimarina* TZS-4 | [138] |
| 126 | *Shewanella nanhaiensis* NR704-98 | [139] |


1. Kuever, J.; Rainey, F.A.; Widdel, F. *Desulfovibrio*. In *Bergey's Manual of Systematics of Archaea and Bacteria*; 2015; pp. 1-17.
2. Heidelberg, J.F.; Seshadri, R.; Haveman, S.A.; Hemme, C.L.; Paulsen, I.T.; Kolonay, J.F.; Eisen, J.A.; Ward, N.; Methe, B.; Brinkac, L.M.; et al. The genome sequence of the anaerobic, sulfate-reducing bacterium *Desulfovibrio vulgaris* Hildenborough. *Nat Biotechnol* **2004**, *22*, 554-559, doi:10.1038/nbt959.
3. Liu, D.; Dong, H.; Bishop, M.E.; Zhang, J.; Wang, H.; Xie, S.; Wang, S.; Huang, L.; Eberl, D.D. Microbial reduction of structural iron in interstratified illite-smectite minerals by a sulfate-reducing bacterium. *Geobiology* **2012**, *10*, 150-162, doi:https://doi.org/10.1111/j.1472-4669.2011.00307.x.
4. Campbell, L.L.; Kasprzycki, M.A.; Postgate, J.R. *Desulfovibrio africanus* sp. n., a New Dissimilatory Sulfate-reducing Bacterium. *Journal of Bacteriology* **1966**, *92*, 1122-1127, doi:doi:10.1128/jb.92.4.1122-1127.1966.
5. Postgate, J.R.; Campbell, L.L. Classification of *Desulfovibrio* species, the nonsporulating sulfate-reducing bacteria. *Bacteriol Rev* **1966**, *30*, 732-738, doi:10.1128/br.30.4.732-738.1966.

6. Motamedi, M.; Pedersen, K. *Desulfovibrio aespoeensis* sp. nov., a mesophilic sulfate-reducing bacterium from deep groundwater at Äspö hard rock laboratory, Sweden *International Journal of Systematic and Evolutionary Microbiology* **1998**, *48*, 311-315, doi:https://doi.org/10.1099/00207713-48-1-311.
7. Sakaguchi, T.; Arakaki, A.; Matsunaga, T. *Desulfovibrio magneticus* sp. nov., a novel sulfate-reducing bacterium that produces intracellular single-domain-sized magnetite particles. *International Journal of Systematic and Evolutionary Microbiology* **2002**, *52*, 215-221, doi:https://doi.org/10.1099/00207713-52-1-215.
8. Legall, J. A New species of *Desulfovibrio*. *J Bacteriol* **1963**, *86*, 1120, doi:10.1128/jb.86.5.1120-1120.1963.
9. Loubinoux, J.; Valente, F.M.A.; Pereira, I.A.C.; Costa, A.; Grimont, P.A.D.; Le Faou, A.E. Reclassification of the only species of the genus *Desulfomonas*, *Desulfomonas pigra,* as *Desulfovibrio piger* comb. nov. *International Journal of Systematic and Evolutionary Microbiology* **2002**, *52*, 1305-1308, doi:https://doi.org/10.1099/00207713-52-4-1305.
10. Krekeler, D.; Sigalevich, P.; Teske, A.; Cypionka, H.; Cohen, Y. A sulfate-reducing bacterium from the oxic layer of a microbial mat from Solar Lake (Sinai), *Desulfovibrio oxyclinae* sp. nov. *Archives of Microbiology* **1997**, *167*, 369-375, doi:10.1007/s002030050457.
11. Sass, H.; Berchtold, M.; Branke, J.; König, H.; Cypionka, H.; Babenzien, H.-D. Psychrotolerant Sulfate-reducing Bacteria from an Oxic Freshwater Sediment Description of *Desulfovibrio cuneatus* sp. nov. and *Desulfovibrio litoralis* sp. nov. *Systematic and Applied Microbiology* **1998**, *21*, 212-219, doi:https://doi.org/10.1016/S0723-2020(98)80025-8.
12. Klouche, N.; Basso, O.; Lascourrèges, J.-F.; Cayol, J.-L.; Thomas, P.; Fauque, G.; Fardeau, M.-L.; Magot, M. *Desulfocurvus vexinensis* gen. nov., sp. nov., a sulfate-reducing bacterium isolated from a deep subsurface aquifer. *International Journal of Systematic and Evolutionary Microbiology* **2009**, *59*, 3100-3104, doi:https://doi.org/10.1099/ijs.0.010363-0.
13. Abildgaard, L.; Nielsen, M.B.; Kjeldsen, K.U.; Ingvorsen, K. *Desulfovibrio alkalitolerans* sp. nov., a novel alkalitolerant, sulphate-reducing bacterium isolated from district heating water. *International Journal of Systematic and Evolutionary Microbiology* **2006**, *56*, 1019-1024, doi:https://doi.org/10.1099/ijs.0.63909-0.
14. Ollivier, B.; Cord-Ruwisch, R.; Hatchikian, E.C.; Garcia, J.L. Characterization of *Desulfovibrio fructosovorans* sp. nov. *Archives of Microbiology* **1988**, *149*, 447-450, doi:10.1007/BF00425586.
15. Vandieken, V.; Knoblauch, C.; Jørgensen, B.B. *Desulfovibrio frigidus* sp. nov. and *Desulfovibrio ferrireducens* sp. nov., psychrotolerant bacteria isolated from Arctic fjord sediments (Svalbard) with the ability to reduce Fe(III). *International Journal of Systematic and Evolutionary Microbiology* **2006**, *56*, 681-685, doi:https://doi.org/10.1099/ijs.0.64057-0.
16. Alazard, D.; Dukan, S.; Urios, A.; Verhé, F.; Bouabida, N.; Morel, F.; Thomas, P.; Garcia, J.-L.; Ollivier, B. *Desulfovibrio hydrothermalis* sp. nov., a novel sulfate-reducing bacterium isolated from hydrothermal vents. *International Journal of Systematic and Evolutionary Microbiology* **2003**, *53*, 173-178, doi:https://doi.org/10.1099/ijs.0.02323-0.
17. Brown, S.D.; Hurt, R.A., Jr.; Gilmour, C.C.; Elias, D.A. Draft genome sequences for three mercury-methylating, sulfate-reducing bacteria. *Genome Announc* **2013**, *1*, doi:10.1128/genomeA.00618-13.

18. Karnachuk, O.V.; Sasaki, K.; Gerasimchuk, A.L.; Sukhanova, O.; Ivasenko, D.A.; Kaksonen, A.H.; Puhakka, J.A.; Tuovinen, O.H. Precipitation of Cu-Sulfides by Copper-Tolerant *Desulfovibrio* Isolates. *Geomicrobiology Journal* **2008**, *25*, 219-227, doi:10.1080/01490450802153124.
19. Mancini, S.; Abicht, H.K.; Karnachuk, O.V.; Solioz, M. Genome sequence of *Desulfovibrio* sp. A2, a highly copper resistant, sulfate-reducing bacterium isolated from effluents of a zinc smelter at the Urals. *J Bacteriol* **2011**, *193*, 6793-6794, doi:10.1128/jb.06019-11.
20. Baena, S.; Fardeau, M.L.; Labat, M.; Ollivier, B.; Garcia, J.L.; Patel, B.K. *Desulfovibrio aminophilus* sp. nov., a novel amino acid degrading and sulfate reducing bacterium from an anaerobic dairy wastewater lagoon. *Syst Appl Microbiol* **1998**, *21*, 498-504, doi:10.1016/s0723-2020(98)80061-1.
21. Khelaifia, S.; Fardeau, M.-L.; Pradel, N.; Aussignargues, C.; Garel, M.; Tamburini, C.; Cayol, J.-L.; Gaudron, S.; Gaill, F.; Ollivier, B. *Desulfovibrio piezophilus* sp. nov., a piezophilic, sulfate-reducing bacterium isolated from wood falls in the Mediterranean Sea. *International Journal of Systematic and Evolutionary Microbiology* **2011**, *61*, 2706-2711, doi:https://doi.org/10.1099/ijs.0.028670-0.
22. Feio, M.J.; Zinkevich, V.; Beech, I.B.; Llobet-Brossa, E.; Eaton, P.; Schmitt, J.; Guezennec, J. *Desulfovibrio alaskensis* sp. nov., a sulphate-reducing bacterium from a soured oil reservoir. *International Journal of Systematic and Evolutionary Microbiology* **2004**, *54*, 1747-1752, doi:https://doi.org/10.1099/ijs.0.63118-0.
23. Ramsay, B.D.; Hwang, C.; Woo, H.L.; Carroll, S.L.; Lucas, S.; Han, J.; Lapidus, A.L.; Cheng, J.F.; Goodwin, L.A.; Pitluck, S.; et al. High-Quality Draft Genome Sequence of *Desulfovibrio carbinoliphilus* FW-101-2B, an Organic Acid-Oxidizing Sulfate-Reducing Bacterium Isolated from Uranium(VI)-Contaminated Groundwater. *Genome Announc* **2015**, *3*, doi:10.1128/genomeA.00092-15.
24. Zhou, J.; He, Q.; Hemme, C.L.; Mukhopadhyay, A.; Hillesland, K.; Zhou, A.; He, Z.; Van Nostrand, J.D.; Hazen, T.C.; Stahl, D.A.; et al. How sulphate-reducing microorganisms cope with stress: lessons from systems biology. *Nature Reviews Microbiology* **2011**, *9*, 452-466, doi:10.1038/nrmicro2575.
25. Magot, M.; Basso, O.; Tardy-Jacquenod, C.; Caumette, P. *Desulfovibrio bastinii* sp. nov. and *Desulfovibrio gracilis* sp. nov., moderately halophilic, sulfate-reducing bacteria isolated from deep subsurface oilfield water. *International Journal of Systematic and Evolutionary Microbiology* **2004**, *54*, 1693-1697, doi:https://doi.org/10.1099/ijs.0.02977-0.
26. Qatibi, A.-I.; Nivière, V.; Garcia, J. Desulfovibrio alcoholovorans sp. nov., a sulfate-reducing bacterium able to grow on glycerol, 1,2- and 1,3-propanediol. *Archives of Microbiology* **1991**, *155*, 143-148, doi:10.1007/BF00248608.
27. Lovley, D.R.; Roden, E.E.; Phillips, E.J.P.; Woodward, J.C. Enzymatic iron and uranium reduction by sulfate-reducing bacteria. *Marine Geology* **1993**, *113*, 41-53, doi:https://doi.org/10.1016/0025-3227(93)90148-O.
28. Nielsen, J.T.; Liesack, W.; Finster, K. *Desulfovibrio zosterae* sp. nov., a new sulfate reducer isolated from surface-sterilized roots of the seagrass *Zostera marina*. *International Journal of Systematic and Evolutionary Microbiology* **1999**, *49*, 859-865, doi:https://doi.org/10.1099/00207713-49-2-859.
29. Basso, O.; Caumette, P.; Magot, M. *Desulfovibrio putealis* sp. nov., a novel sulfate-reducing bacterium isolated from a deep subsurface aquifer. *International Journal of Systematic and Evolutionary Microbiology* **2005**, *55*, 101-104, doi:https://doi.org/10.1099/ijs.0.63303-0.

30. Reichenbecher, W.; Schink, B. *Desulfovibrio inopinatus*, sp. nov., a new sulfate-reducing bacterium that degrades hydroxyhydroquinone (1,2,4-trihydroxybenzene). *Archives of Microbiology* **1997**, *168*, 338-344, doi:10.1007/s002030050507.
31. Magot, M.; Caumette, P.; Desperrier, J.M.; Matheron, R.; Dauga, C.; Grimont, F.; Carreau, L. *Desulfovibrio longus* sp. nov., a Sulfate-Reducing Bacterium Isolated from an Oil-Producing Well. *International Journal of Systematic and Evolutionary Microbiology* **1992**, *42*, 398-402, doi:https://doi.org/10.1099/00207713-42-3-398.
32. Gilmour, C.C.; Elias, D.A.; Kucken, A.M.; Brown, S.D.; Palumbo, A.V.; Schadt, C.W.; Wall, J.D. Sulfate-reducing bacterium *Desulfovibrio desulfuricans* ND132 as a model for understanding bacterial mercury methylation. *Appl Environ Microbiol* **2011**, *77*, 3938-3951, doi:10.1128/aem.02993-10.
33. Gilmour, C.C.; Soren, A.B.; Gionfriddo, C.M.; Podar, M.; Wall, J.D.; Brown, S.D.; Michener, J.K.; Urriza, M.S.G.; Elias, D.A. *Pseudodesulfovibrio mercurii* sp. nov., a mercury-methylating bacterium isolated from sediment. *International Journal of Systematic and Evolutionary Microbiology* **2021**, *71*, doi:https://doi.org/10.1099/ijsem.0.004697.
34. Brown, S.D.; Utturkar, S.M.; Arkin, A.P.; Deutschbauer, A.M.; Elias, D.A.; Hazen, T.C.; Chakraborty, R. Draft Genome Sequence for *Desulfovibrio africanus* Strain PCS. *Genome Announcements* **2013**, *1*, 10.1128/genomea.00144-00113, doi:doi:10.1128/genomea.00144-13.
35. Wolicka, D.; Jarzynowska, L. Microbiological Reduction of Sulphates in Salty Environments and Mineralogical Characterization of the Transformation Products. *Geomicrobiology Journal* **2012**, *29*, 528-536, doi:10.1080/01490451.2011.590873.
36. Karnachuk, O.V.; Rusanov, I.I.; Panova, I.A.; Grigoriev, M.A.; Zyusman, V.S.; Latygolets, E.A.; Kadyrbaev, M.K.; Gruzdev, E.V.; Beletsky, A.V.; Mardanov, A.V.; et al. Microbial sulfate reduction by Desulfovibrio is an important source of hydrogen sulfide from a large swine finishing facility. *Scientific Reports* **2021**, *11*, 10720, doi:10.1038/s41598-021-90256-w.
37. Boeckman, J.; Korn, A.; Yao, G.; Ravindran, A.; Gonzalez, C.; Gill, J. Sheep in wolves' clothing: Temperate T7-like bacteriophages and the origins of the Autographiviridae. *Virology* **2022**, *568*, 86-100, doi:https://doi.org/10.1016/j.virol.2022.01.013.
38. HATCHIKIAN, E.C.; FORGET, N.; FERNANDEZ, V.M.; WILLIAMS, R.; CAMMACK, R. Further characterization of the [Fe]-hydrogenase from Desulfovibrio desulfuricans ATCC 7757. *European Journal of Biochemistry* **1992**, *209*, 357-365, doi:https://doi.org/10.1111/j.1432-1033.1992.tb17297.x.
39. Day, L.A.; León, K.B.D.; Kempher, M.L.; Zhou, J.; Wall, J.D. Complete Genome Sequence of Desulfovibrio desulfuricans IC1, a Sulfonate-Respiring Anaerobe. *Microbiology Resource Announcements* **2019**, *8*, 10.1128/mra.00456-00419, doi:doi:10.1128/mra.00456-19.
40. Lie, T.J.; Pitta, T.; Leadbetter, E.R.; Godchaux Iii, W.; Leadbetter, J.R. Sulfonates: novel electron acceptors in anaerobic respiration. *Archives of Microbiology* **1996**, *166*, 204-210, doi:10.1007/s002030050376.
41. Sheik, C.S.; Sieber, J.R.; Badalamenti, J.P.; Carden, K.; Olson, A. Complete Genome Sequence of Desulfovibrio desulfuricans Strain G11, a Model Sulfate-Reducing, Hydrogenotrophic, and Syntrophic Partner Organism. *Genome Announcements* **2017**, *5*, 10.1128/genomea.01207-01217, doi:doi:10.1128/genomea.01207-17.

42. McInerney, M.J.; Bryant, M.P.; Pfennig, N. Anaerobic bacterium that degrades fatty acids in syntrophic association with methanogens. *Archives of Microbiology* **1979**, *122*, 129-135, doi:10.1007/BF00411351.
43. Karnachuk, O.V.; Ikkert, O.P.; Avakyan, M.R.; Knyazev, Y.V.; M, N.V.; Zyusman, V.S.; Panov, V.L.; Kadnikov, V.V.; Mardanov, A.V.; Ravin, N.V. Desulfovibrio desulfuricans AY5 Isolated from a Patient with Autism Spectrum Disorder Binds Iron in Low-Soluble Greigite and Pyrite. *Microorganisms* **2021**, *9*, doi:10.3390/microorganisms9122558.
44. Sun, D.; Wang, A.; Cheng, S.; Yates, M.; Logan, B.E. Geobacter anodireducens sp. nov., an exoelectrogenic microbe in bioelectrochemical systems. *Int J Syst Evol Microbiol* **2014**, *64*, 3485-3491, doi:10.1099/ijs.0.061598-0.
45. Shelobolina, E.S.; Nevin, K.P.; Blakeney-Hayward, J.D.; Johnsen, C.V.; Plaia, T.W.; Krader, P.; Woodard, T.; Holmes, D.E.; VanPraagh, C.G.; Lovley, D.R. *Geobacter pickeringii* sp. nov., *Geobacter argillaceus* sp. nov. and *Pelosinus fermentans* gen. nov., sp. nov., isolated from subsurface kaolin lenses. *International Journal of Systematic and Evolutionary Microbiology* **2007**, *57*, 126-135, doi:https://doi.org/10.1099/ijs.0.64221-0.
46. Coates, J.D.; Bhupathiraju, V.K.; Achenbach, L.A.; Mclnerney, M.J.; Lovley, D.R. Geobacter hydrogenophilus, Geobacter chapellei and Geobacter grbiciae, three new, strictly anaerobic, dissimilatory Fe(III)-reducers. *International Journal of Systematic and Evolutionary Microbiology* **2001**, *51*, 581-588, doi:https://doi.org/10.1099/00207713-51-2-581.
47. Prakash, O.; Gihring, T.M.; Dalton, D.D.; Chin, K.-J.; Green, S.J.; Akob, D.M.; Wanger, G.; Kostka, J.E. Geobacter daltonii sp. nov., an Fe(III)- and uranium(VI)-reducing bacterium isolated from a shallow subsurface exposed to mixed heavy metal and hydrocarbon contamination. *International Journal of Systematic and Evolutionary Microbiology* **2010**, *60*, 546-553, doi:https://doi.org/10.1099/ijs.0.010843-0.
48. Viulu, S.; Nakamura, K.; Okada, Y.; Saitou, S.; Takamizawa, K. Geobacterluticola sp. nov., an Fe(III)-reducing bacterium isolated from lotus field mud. *International Journal of Systematic and Evolutionary Microbiology* **2013**, *63*, 442-448, doi:https://doi.org/10.1099/ijs.0.039321-0.
49. Childers, S.E.; Ciufo, S.; Lovley, D.R. Geobacter metallireducens accesses insoluble Fe(III) oxide by chemotaxis. *Nature* **2002**, *416*, 767-769, doi:10.1038/416767a.
50. Aklujkar, M.; Krushkal, J.; DiBartolo, G.; Lapidus, A.; Land, M.L.; Lovley, D.R. The genome sequence of Geobacter metallireducens: features of metabolism, physiology and regulation common and dissimilar to Geobacter sulfurreducens. *BMC Microbiology* **2009**, *9*, 109, doi:10.1186/1471-2180-9-109.
51. Lovley, D.R.; Phillips, E.J. Novel mode of microbial energy metabolism: organic carbon oxidation coupled to dissimilatory reduction of iron or manganese. *Appl Environ Microbiol* **1988**, *54*, 1472-1480, doi:10.1128/aem.54.6.1472-1480.1988.
52. Badalamenti, J.P.; Bond, D.R. Complete Genome of Geobacter pickeringii G13<sup>T</sup>, a Metal-Reducing Isolate from Sedimentary Kaolin Deposits. *Genome Announcements* **2015**, *3*, 10.1128/genomea.00038-00015, doi:doi:10.1128/genomea.00038-15.
53. Yang, G.; Chen, S.; Zhou, S.; Liu, Y. Genome sequence of a dissimilatory Fe(III)-reducing bacterium Geobacter soli type strain GSS01(T). *Stand Genomic Sci* **2015**, *10*, 118, doi:10.1186/s40793-015-0117-7.

54. Guo, Y.; Aoyagi, T.; Hori, T. Draft Genome Sequences of the Ferric Iron-Reducing Geobacter sp. Strains AOG1 and AOG2, Isolated from Enrichment Cultures on Crystalline Iron(III) Oxides. *Microbiol Resour Announc* **2021**, *10*, e0091321, doi:10.1128/mra.00913-21.
55. Hori, T.; Aoyagi, T.; Itoh, H.; Narihiro, T.; Oikawa, A.; Suzuki, K.; Ogata, A.; Friedrich, M.W.; Conrad, R.; Kamagata, Y. Isolation of microorganisms involved in reduction of crystalline iron(III) oxides in natural environments. *Frontiers in Microbiology* **2015**, *6*, doi:10.3389/fmicb.2015.00386.
56. Yadav, P.; Antony-Babu, S.; Hayes, E.; Healy, O.M.; Pan, D.; Yang, W.H.; Silver, W.L.; Anderson, C.L.; Voshall, A.; Fernando, S.C.; et al. Complete Genome Sequence of <i>Geobacter</i> sp. Strain FeAm09, a Moderately Acidophilic Soil Bacterium. *Microbiology Resource Announcements* **2021**, *10*, 10.1128/mra.00979-00920, doi:doi:10.1128/mra.00979-20.
57. Yang, G.; Li, Y.; Lin, A.; Zhuang, L. Geobacter benzoatilyticus sp. nov., a novel benzoate-oxidizing, iron-reducing bacterium isolated from petroleum contaminated soil. *International Journal of Systematic and Evolutionary Microbiology* **2022**, *72*, doi:https://doi.org/10.1099/ijsem.0.005281.
58. Ehara, A.; Suzuki, H.; Amachi, S. Draft Genome Sequence of Geobacter sp. Strain OR-1, an Arsenate-Respiring Bacterium Isolated from Japanese Paddy Soil. *Genome Announc* **2015**, *3*, doi:10.1128/genomeA.01478-14.
59. Ohtsuka, T.; Yamaguchi, N.; Makino, T.; Sakurai, K.; Kimura, K.; Kudo, K.; Homma, E.; Dong, D.T.; Amachi, S. Arsenic Dissolution from Japanese Paddy Soil by a Dissimilatory Arsenate-Reducing Bacterium Geobacter sp. OR-1. *Environmental Science & Technology* **2013**, *47*, 6263-6271, doi:10.1021/es400231x.
60. Warashina, T.; Yamamura, S.; Suzuki, H.; Amachi, S.; Arakawa, K. Complete Genome Sequence of Geobacter sp. Strain SVR, an Antimonate-Reducing Bacterium Isolated from Antimony-Rich Mine Soil. *Microbiology Resource Announcements* **2021**, *10*, 10.1128/mra.00142-00121, doi:10.1128/mra.00142-21.
61. Warashina, T.; Harada, M.; Nakajima, N.; Yamamura, S.; Tomita, M.; Suzuki, H.; Amachi, S. Draft Genome Sequence of <i>Geobacter</i> sp. Strain SVR, Isolated from Antimony Mine Soil. *Microbiology Resource Announcements* **2020**, *9*, 10.1128/mra.00461-00420, doi:doi:10.1128/mra.00461-20.
62. Yamamura, S.; Iida, C.; Kobayashi, Y.; Watanabe, M.; Amachi, S. Production of two morphologically different antimony trioxides by a novel antimonate-reducing bacterium, Geobacter sp. SVR. *J Hazard Mater* **2021**, *411*, 125100, doi:10.1016/j.jhazmat.2021.125100.
63. Nagarajan, H.; Butler, J.E.; Klimes, A.; Qiu, Y.; Zengler, K.; Ward, J.; Young, N.D.; Methé, B.A.; Palsson, B.; Lovley, D.R.; et al. De Novo assembly of the complete genome of an enhanced electricity-producing variant of Geobacter sulfurreducens using only short reads. *PLoS One* **2010**, *5*, e10922, doi:10.1371/journal.pone.0010922.
64. Yi, H.; Nevin, K.P.; Kim, B.C.; Franks, A.E.; Klimes, A.; Tender, L.M.; Lovley, D.R. Selection of a variant of Geobacter sulfurreducens with enhanced capacity for current production in microbial fuel cells. *Biosens Bioelectron* **2009**, *24*, 3498-3503, doi:10.1016/j.bios.2009.05.004.

65. Caccavo, F., Jr.; Lonergan, D.J.; Lovley, D.R.; Davis, M.; Stolz, J.F.; McInerney, M.J. Geobacter sulfurreducens sp. nov., a hydrogen- and acetate-oxidizing dissimilatory metal-reducing microorganism. *Appl Environ Microbiol* **1994**, *60*, 3752-3759, doi:10.1128/aem.60.10.3752-3759.1994.
66. Methé, B.A.; Nelson, K.E.; Eisen, J.A.; Paulsen, I.T.; Nelson, W.; Heidelberg, J.F.; Wu, D.; Wu, M.; Ward, N.; Beanan, M.J.; et al. Genome of Geobacter sulfurreducens: metal reduction in subsurface environments. *Science* **2003**, *302*, 1967-1969, doi:10.1126/science.1088727.
67. Inoue, K.; Ogura, Y.; Kawano, Y.; Hayashi, T. Complete Genome Sequence of Geobacter sulfurreducens Strain YM18, Isolated from River Sediment in Japan. *Genome Announc* **2018**, *6*, doi:10.1128/genomeA.00352-18.
68. Fujikawa, T.; Ogura, Y.; Ishigami, K.; Kawano, Y.; Nagamine, M.; Hayashi, T.; Inoue, K. Unexpected genomic features of high current density-producing Geobacter sulfurreducens strain YM18. *FEMS Microbiology Letters* **2021**, *368*, doi:10.1093/femsle/fnab119.
69. Fujikawa, T.; Ogura, Y.; Hayashi, T.; Inoue, K. Complete Genome Sequence of High Current-Producing Geobacter sulfurreducens Strain YM35, Isolated from River Sediment in Japan. *Microbiol Resour Announc* **2021**, *10*, e0053921, doi:10.1128/mra.00539-21.
70. Shelobolina, E.S.; Vrionis, H.A.; Findlay, R.H.; Lovley, D.R. Geobacter uraniireducens sp. nov., isolated from subsurface sediment undergoing uranium bioremediation. *International Journal of Systematic and Evolutionary Microbiology* **2008**, *58*, 1075-1078, doi:https://doi.org/10.1099/ijs.0.65377-0.
71. Semple, K.M.; Westlake, D.W.S. Characterization of iron-reducing Alteromonas putrefaciens strains from oil field fluids. *Canadian Journal of Microbiology* **1987**, *33*, 366-371, doi:10.1139/m87-064.
72. Kim, J.-Y.; Yoo, H.-S.; Lee, D.-H.; Park, S.-H.; Kim, Y.-J.; Oh, D.-C. Shewanella algicola sp. nov., a marine bacterium isolated from brown algae. *International Journal of Systematic and Evolutionary Microbiology* **2016**, *66*, 2218-2224, doi:https://doi.org/10.1099/ijsem.0.001014.
73. Ohama, Y.; Aoki, K.; Harada, S.; Nagasawa, T.; Sawabe, T.; Nonaka, L.; Moriya, K.; Ishii, Y.; Tateda, K. Genetic Environment Surrounding <i>bla</i><sub>OXA-55-like</sub> in Clinical Isolates of Shewanella algae Clade and Enhanced Expression of <i>bla</i><sub>OXA-55-like</sub> in a Carbapenem-Resistant Isolate. *mSphere* **2021**, *6*, 10.1128/msphere.00593-00521, doi:doi:10.1128/msphere.00593-21.
74. Wang, Y.; Chen, H.; Liu, Z.; Ming, H.; Zhou, C.; Zhu, X.; Zhang, P.; Jing, C.; Feng, H. Shewanella gelidii sp. nov., isolated from the red algae Gelidium amansii, and emended description of Shewanella waksmanii. *International Journal of Systematic and Evolutionary Microbiology* **2016**, *66*, 2899-2905, doi:https://doi.org/10.1099/ijsem.0.001075.
75. Satomi, M.; Vogel, B.F.; Venkateswaran, K.; Gram, L. Description of Shewanella glacialipiscicola sp. nov. and Shewanella algidipiscicola sp. nov., isolated from marine fish of the Danish Baltic Sea, and proposal that Shewanella affinis is a later heterotypic synonym of Shewanella colwelliana. *International Journal of Systematic and Evolutionary Microbiology* **2007**, *57*, 347-352, doi:https://doi.org/10.1099/ijs.0.64708-0.
76. Satomi, M.; Vogel, B.F.; Gram, L.; Venkateswaran, K. Shewanella hafniensis sp. nov. and Shewanella morhuae sp. nov., isolated from marine fish of the Baltic Sea. *International Journal of Systematic and Evolutionary Microbiology* **2006**, *56*, 243-249, doi:https://doi.org/10.1099/ijs.0.63931-0.

77. Prachumwat, A.; Wechprasit, P.; Srisala, J.; Kriangsaksri, R.; Flegel, T.W.; Thitamadee, S.; Sritunyalucksana, K. Shewanella khirikhana sp. nov. - a shrimp pathogen isolated from a cultivation pond exhibiting early mortality syndrome. *Microb Biotechnol* **2020**, *13*, 781-795, doi:10.1111/1751-7915.13538.
78. Wechprasit, P.; Panphloi, M.; Thitamadee, S.; Sritunyalucksana, K.; Prachumwat, A. Complete Genome Sequence of Shewanella sp. Strain TH2012, Isolated from Shrimp in a Cultivation Pond Exhibiting Early Mortality Syndrome. *Microbiol Resour Announc* **2019**, *8*, doi:10.1128/mra.01703-18.
79. Leonardo, M.R.; Moser, D.P.; Barbieri, E.; Brantner, C.A.; MacGregor, B.J.; Paster, B.J.; Stackebrandt, E.; Nealson, K.H. Shewanella pealeana sp. nov., a member of the microbial community associated with the accessory nidamental gland of the squid Loligo pealei. *Int J Syst Bacteriol* **1999**, *49 Pt 4*, 1341-1351, doi:10.1099/00207713-49-4-1341.
80. Cha, Q.-Q.; Ren, X.-B.; Sun, Y.-Y.; He, X.-Y.; Su, H.-N.; Chen, X.-L.; Zhang, Y.-Z.; Xie, B.-B.; Zhao, L.-S.; Song, X.-Y.; et al. Shewanella polaris sp. nov., a psychrotolerant bacterium isolated from Arctic brown algae. *International Journal of Systematic and Evolutionary Microbiology* **2020**, *70*, 2096-2102, doi:https://doi.org/10.1099/ijsem.0.004022.
81. Gai, Y.; Huang, Z.; Lai, Q.; Shao, Z. Shewanella intestini sp. nov., isolated from the intestine of abalone, Haliotis diversicolor. *International Journal of Systematic and Evolutionary Microbiology* **2017**, *67*, 1901-1905, doi:https://doi.org/10.1099/ijsem.0.001878.
82. Satomi, M.; Oikawa, H.; Yano, Y. Shewanella marinintestina sp. nov., Shewanella schlegeliana sp. nov. and Shewanella sairae sp. nov., novel eicosapentaenoic-acid-producing marine bacteria isolated from sea-animal intestines. *International Journal of Systematic and Evolutionary Microbiology* **2003**, *53*, 491-499, doi:https://doi.org/10.1099/ijs.0.02392-0.
83. Cerbino, G.N.; Traglia, G.M.; Ayala Nuñez, T.; Parmeciano Di Noto, G.; Ramírez, M.S.; Centrón, D.; Iriarte, A.; Quiroga, C. Comparative genome analysis of the genus Shewanella unravels the association of key genetic traits with known and potential pathogenic lineages. *Front Microbiol* **2023**, *14*, 1124225, doi:10.3389/fmicb.2023.1124225.
84. Brian-Jaisson, F.; Ortalo-Magné, A.; Guentas-Dombrowsky, L.; Armougom, F.; Blache, Y.; Molmeret, M. Identification of bacterial strains isolated from the Mediterranean Sea exhibiting different abilities of biofilm formation. *Microb Ecol* **2014**, *68*, 94-110, doi:10.1007/s00248-013-0342-9.
85. Guillonneau, R.; Baraquet, C.; Bazire, A.; Molmeret, M. Multispecies Biofilm Development of Marine Bacteria Implies Complex Relationships Through Competition and Synergy and Modification of Matrix Components. *Frontiers in Microbiology* **2018**, *9*, doi:10.3389/fmicb.2018.01960.
86. Caro-Quintero, A.; Auchtung, J.; Deng, J.; Brettar, I.; Höfle, M.; Tiedje, J.M.; Konstantinidis, K.T. Genome sequencing of five Shewanella baltica strains recovered from the oxic-anoxic interface of the Baltic Sea. *J Bacteriol* **2012**, *194*, 1236, doi:10.1128/jb.06468-11.
87. Deng, J.; Brettar, I.; Luo, C.; Auchtung, J.; Konstantinidis, K.T.; Rodrigues, J.L.; Höfle, M.; Tiedje, J.M. Stability, genotypic and phenotypic diversity of Shewanella baltica in the redox transition zone of the Baltic Sea. *Environ Microbiol* **2014**, *16*, 1854-1866, doi:10.1111/1462-2920.12344.

88. Brettar, I.; Christen, R.; Höfle, M.G. Shewanella denitrificans sp. nov., a vigorously denitrifying bacterium isolated from the oxic-anoxic interface of the Gotland Deep in the central Baltic Sea. *International Journal of Systematic and Evolutionary Microbiology* **2002**, *52*, 2211-2217, doi:https://doi.org/10.1099/00207713-52-6-2211.
89. Sung, H.-R.; Yoon, J.-H.; Ghim, S.-Y. Shewanella dokdonensis sp. nov., isolated from seawater. *International Journal of Systematic and Evolutionary Microbiology* **2012**, *62*, 1636-1643, doi:https://doi.org/10.1099/ijs.0.032995-0.
90. Reid, G.A.; Gordon, E.H.J. Phylogeny of marine and freshwater Shewanella: reclassification of Shewanella putrefaciens NCIMB 400 as Shewanella frigidimarina. *International Journal of Systematic and Evolutionary Microbiology* **1999**, *49*, 189-191, doi:https://doi.org/10.1099/00207713-49-1-189.
91. Reyes-Ramirez, F.; Dobbin, P.; Sawers, G.; Richardson David, J. Characterization of Transcriptional Regulation of Shewanella frigidimarina Fe(III)-Induced Flavocytochrome c Reveals a Novel Iron-Responsive Gene Regulation System. *Journal of Bacteriology* **2003**, *185*, 4564-4571, doi:10.1128/jb.185.15.4564-4571.2003.
92. Kim, K.M.; Choe, H.; Kim, B.K.; Nasir, A. Complete genome of a metabolically-diverse marine bacterium Shewanella japonica KCTC 22435(T). *Mar Genomics* **2017**, *35*, 39-42, doi:10.1016/j.margen.2017.05.004.
93. Biffinger, J.C.; Fitzgerald, L.A.; Ray, R.; Little, B.J.; Lizewski, S.E.; Petersen, E.R.; Ringeisen, B.R.; Sanders, W.C.; Sheehan, P.E.; Pietron, J.J.; et al. The utility of Shewanella japonica for microbial fuel cells. *Bioresource Technology* **2011**, *102*, 290-297, doi:https://doi.org/10.1016/j.biortech.2010.06.078.
94. Ivanova, E.P.; Sawabe, T.; Gorshkova, N.M.; Svetashev, V.I.; Mikhailov, V.V.; Nicolau, D.V.; Christen, R. Shewanella japonica sp. nov. *International Journal of Systematic and Evolutionary Microbiology* **2001**, *51*, 1027-1033, doi:https://doi.org/10.1099/00207713-51-3-1027.
95. Yun, B.-R.; Park, S.; Kim, M.-K.; Park, J.; Kim, S.B. Shewanella saliphila sp. nov., Shewanella ulleungensis sp. nov. and Shewanella litoralis sp. nov., isolated from coastal seawater. *International Journal of Systematic and Evolutionary Microbiology* **2018**, *68*, 2960-2966, doi:https://doi.org/10.1099/ijsem.0.002929.
96. Bozal, N.; Montes, M.J.; Tudela, E.; Jiménez, F.; Guinea, J. Shewanella frigidimarina and Shewanella livingstonensis sp. nov. isolated from Antarctic coastal areas. *International Journal of Systematic and Evolutionary Microbiology* **2002**, *52*, 195-205, doi:https://doi.org/10.1099/00207713-52-1-195.
97. Park, S.C.; Baik, K.S.; Kim, M.S.; Kim, D.; Seong, C.N. Shewanella marina sp. nov., isolated from seawater. *International Journal of Systematic and Evolutionary Microbiology* **2009**, *59*, 1888-1894, doi:https://doi.org/10.1099/ijs.0.005470-0.
98. Bae, S.S.; Jung, Y.-h.; Baek, K. Shewanella maritima sp. nov., a facultative anaerobic marine bacterium isolated from seawater, and emended description of Shewanella intestini. *International Journal of Systematic and Evolutionary Microbiology* **2020**, *70*, 1288-1293, doi:https://doi.org/10.1099/ijsem.0.003916.

99. Makemson, J.C.; Fulayfil, N.R.; Landry, W.; Van Ert, L.M.; Wimpee, C.F.; Widder, E.A.; Case, J.F. Shewanella woodyi sp. nov., an exclusively respiratory luminous bacterium isolated from the Alboran Sea. *Int J Syst Bacteriol* **1997**, *47*, 1034-1039, doi:10.1099/00207713-47-4-1034.
100. Park, H.Y.; Jeon, C.O. Shewanellaaestuarii sp. nov., a marine bacterium isolated from a tidal flat. *International Journal of Systematic and Evolutionary Microbiology* **2013**, *63*, 4683-4690, doi:https://doi.org/10.1099/ijs.0.055178-0.
101. Baaziz, H.; Lemaire, O.N.; Jourlin-Castelli, C.; Iobbi-Nivol, C.; Méjean, V.; Alatou, R.; Fons, M. Draft Genome Sequence of Shewanella algidipiscicola H1, a Highly Chromate-Resistant Strain Isolated from Mediterranean Marine Sediments. *Microbiol Resour Announc* **2018**, *7*, doi:10.1128/mra.00905-18.
102. Venkateswaran, K.; Dollhopf, M.E.; Aller, R.; Stackebrandt, E.; Nealson, K.H. Shewanella amazonensis sp. nov., a novel metal-reducing facultative anaerobe from Amazonian shelf muds. *Int J Syst Bacteriol* **1998**, *48 Pt 3*, 965-972, doi:10.1099/00207713-48-3-965.
103. Zhao, J.-S.; Manno, D.; Thiboutot, S.; Ampleman, G.; Hawari, J. Shewanella canadensis sp. nov. and Shewanella atlantica sp. nov., manganese dioxide- and hexahydro-1,3,5-trinitro-1,3,5-triazine-reducing, psychrophilic marine bacteria. *International Journal of Systematic and Evolutionary Microbiology* **2007**, *57*, 2155-2162, doi:https://doi.org/10.1099/ijs.0.64596-0.
104. Sucharita, K.; Sasikala, C.; Park, S.C.; Baik, K.S.; Seong, C.N.; Ramana, C.V. Shewanella chilikensis sp. nov., a moderately alkaliphilic gammaproteobacterium isolated from a lagoon. *International Journal of Systematic and Evolutionary Microbiology* **2009**, *59*, 3111-3115, doi:https://doi.org/10.1099/ijs.0.010918-0.
105. Yang, S.-H.; Lee, J.-H.; Ryu, J.-S.; Kato, C.; Kim, S.-J. Shewanella donghaensis sp. nov., a psychrophilic, piezosensitive bacterium producing high levels of polyunsaturated fatty acid, isolated from deep-sea sediments. *International Journal of Systematic and Evolutionary Microbiology* **2007**, *57*, 208-212, doi:https://doi.org/10.1099/ijs.0.64469-0.
106. Ivanova, E.P.; Sawabe, T.; Hayashi, K.; Gorshkova, N.M.; Zhukova, N.V.; Nedashkovskaya, O.I.; Mikhailov, V.V.; Nicolau, D.V.; Christen, R. Shewanella fidelis sp. nov., isolated from sediments and sea water. *International Journal of Systematic and Evolutionary Microbiology* **2003**, *53*, 577-582, doi:https://doi.org/10.1099/ijs.0.02198-0.
107. Verma, P.; Pandey, P.K.; Gupta, A.K.; Kim, H.J.; Baik, K.S.; Seong, C.N.; Patole, M.S.; Shouche, Y.S. Shewanella indica sp. nov., isolated from sediment of the Arabian Sea. *International Journal of Systematic and Evolutionary Microbiology* **2011**, *61*, 2058-2064, doi:https://doi.org/10.1099/ijs.0.026310-0.
108. Wang, M.-q.; Sun, L. Shewanella inventionis sp. nov., isolated from deep-sea sediment. *International Journal of Systematic and Evolutionary Microbiology* **2016**, *66*, 4947-4953, doi:https://doi.org/10.1099/ijsem.0.001450.
109. Lee, M.H.; Yoon, J.H. Shewanella litorisediminis sp. nov., a gammaproteobacterium isolated from a tidal flat sediment. *Antonie Van Leeuwenhoek* **2012**, *102*, 591-599, doi:10.1007/s10482-012-9754-7.
110. Liu, Y.; Shang, X.-X.; Yi, Z.-W.; Gu, L.; Zeng, R.-Y. Shewanella mangrovi sp. nov., an acetaldehyde-degrading bacterium isolated from mangrove sediment. *International Journal of Systematic and Evolutionary Microbiology* **2015**, *65*, 2630-2634, doi:https://doi.org/10.1099/ijs.0.000313.

111. Huang, J.; Sun, B.; Zhang, X. Electricity generation at high ionic strength in microbial fuel cell by a newly isolated Shewanella marisflavi EP1. *Appl Microbiol Biotechnol* **2010**, *85*, 1141-1149, doi:10.1007/s00253-009-2259-2.
112. Huang, J.; Ning, G.; Li, F.; Sheng, G.D. Biotransformation of 2,4-dinitrotoluene by obligate marine Shewanella marisflavi EP1 under anaerobic conditions. *Bioresour Technol* **2015**, *180*, 200-206, doi:10.1016/j.biortech.2014.12.108.
113. Venkateswaran, K.; Moser, D.P.; Dollhopf, M.E.; Lies, D.P.; Saffarini, D.A.; MacGregor, B.J.; Ringelberg, D.B.; White, D.C.; Nishijima, M.; Sano, H.; et al. Polyphasic taxonomy of the genus Shewanella and description of Shewanella oneidensis sp. nov. *International Journal of Systematic and Evolutionary Microbiology* **1999**, *49*, 705-724, doi:https://doi.org/10.1099/00207713-49-2-705.
114. Heidelberg, J.F.; Paulsen, I.T.; Nelson, K.E.; Gaidos, E.J.; Nelson, W.C.; Read, T.D.; Eisen, J.A.; Seshadri, R.; Ward, N.; Methe, B.; et al. Genome sequence of the dissimilatory metal ion–reducing bacterium Shewanella oneidensis. *Nature Biotechnology* **2002**, *20*, 1118-1123, doi:10.1038/nbt749.
115. Xiao, X.; Wang, P.; Zeng, X.; Bartlett, D.H.; Wang, F. Shewanella psychrophila sp. nov. and Shewanella piezotolerans sp. nov., isolated from west Pacific deep-sea sediment. *International Journal of Systematic and Evolutionary Microbiology* **2007**, *57*, 60-65, doi:https://doi.org/10.1099/ijs.0.64500-0.
116. Wang, F.; Wang, J.; Jian, H.; Zhang, B.; Li, S.; Wang, F.; Zeng, X.; Gao, L.; Bartlett, D.H.; Yu, J.; et al. Environmental adaptation: genomic analysis of the piezotolerant and psychrotolerant deep-sea iron reducing bacterium Shewanella piezotolerans WP3. *PLoS One* **2008**, *3*, e1937, doi:10.1371/journal.pone.0001937.
117. Xu, G.; Jian, H.; Xiao, X.; Wang, F. Complete genome sequence of Shewanella psychrophila WP2, a deep-sea bacterium isolated from west Pacific sediment. *Marine Genomics* **2017**, *35*, 19-21, doi:https://doi.org/10.1016/j.margen.2017.03.004.
118. Zhao, J.-S.; Manno, D.; Beaulieu, C.; Paquet, L.; Hawari, J. Shewanella sediminis sp. nov., a novel Na+-requiring and hexahydro-1,3,5-trinitro-1,3,5-triazine-degrading bacterium from marine sediment. *International Journal of Systematic and Evolutionary Microbiology* **2005**, *55*, 1511-1520, doi:https://doi.org/10.1099/ijs.0.63604-0.
119. Li, S.; Niu, Y.; Chen, H.; He, P. Complete genome sequence of an Arctic Ocean bacterium Shewanella sp. Arc9-LZ with capacity of synthesizing silver nanoparticles in darkness. *Marine Genomics* **2021**, *56*, 100808, doi:https://doi.org/10.1016/j.margen.2020.100808.
120. Bujak, K.; Decewicz, P.; Rosinska, J.M.; Radlinska, M. Genome Study of a Novel Virulent Phage vB_SspS_KASIA and Mu-like Prophages of Shewanella sp. M16 Provides Insights into the Genetic Diversity of the Shewanella Virome. *Int J Mol Sci* **2021**, *22*, doi:10.3390/ijms222011070.
121. Yang, Y.; Chen, J.; Qiu, D.; Zhou, J. Roles of UndA and MtrC of *Shewanella putrefaciens* W3-18-1 in iron reduction. *BMC Microbiology* **2013**, *13*, 267, doi:10.1186/1471-2180-13-267.
122. Qiu, D.; Wei, H.; Tu, Q.; Yang, Y.; Xie, M.; Chen, J.; Pinkerton, M.H.; Liang, Y.; He, Z.; Zhou, J. Combined Genomics and Experimental Analyses of Respiratory Characteristics of Shewanella putrefaciens W3-18-1. *Applied and Environmental Microbiology* **2013**, *79*, 5250-5257, doi:doi:10.1128/AEM.00619-13.

123. Bozal, N.; Montes, M.J.; Miñana-Galbis, D.; Manresa, A.; Mercadé, E. *Shewanella vesiculosa* sp. nov., a psychrotolerant bacterium isolated from an Antarctic coastal area. *International Journal of Systematic and Evolutionary Microbiology* **2009**, *59*, 336-340, doi:https://doi.org/10.1099/ijs.0.000737-0.
124. Nogi, Y.; Kato, C.; Horikoshi, K. Taxonomic studies of deep-sea barophilic *Shewanella* strains and description of *Shewanella violacea* sp. nov. *Arch Microbiol* **1998**, *170*, 331-338, doi:10.1007/s002030050650.
125. Chikuma, S.; Kasahara, R.; Kato, C.; Tamegai, H. Bacterial adaptation to high pressure: a respiratory system in the deep-sea bacterium *Shewanella violacea* DSS12. *FEMS Microbiology Letters* **2007**, *267*, 108-112, doi:10.1111/j.1574-6968.2006.00555.x.
126. Aono, E.; Baba, T.; Ara, T.; Nishi, T.; Nakamichi, T.; Inamoto, E.; Toyonaga, H.; Hasegawa, M.; Takai, Y.; Okumura, Y.; et al. Complete genome sequence and comparative analysis of *Shewanella violacea*, a psychrophilic and piezophilic bacterium from deep sea floor sediments. *Molecular BioSystems* **2010**, *6*, 1216-1226, doi:10.1039/C000396D.
127. Jensen, M.J.; Tebo, B.M.; Baumann, P.; Mandel, M.; Nealson, K.H. Characterization of *Alteromonas hanedai* (sp. nov.), a nonfermentative luminous species of marine origin. *Current Microbiology* **1980**, *3*, 311-315, doi:10.1007/BF02601812.
128. Lee, J.-H.; Roh, Y.; Kim, K.-W.; Hur, H.-G. Organic Acid-Dependent Iron Mineral Formation by a Newly Isolated Iron-Reducing Bacterium, *Shewanella* sp. HN-41. *Geomicrobiology Journal* **2007**, *24*, 31-41, doi:10.1080/01490450601134291.
129. Lee, J.H.; Kim, M.G.; Yoo, B.; Myung, N.V.; Maeng, J.; Lee, T.; Dohnalkova, A.C.; Fredrickson, J.K.; Sadowsky, M.J.; Hur, H.G. Biogenic formation of photoactive arsenic-sulfide nanotubes by *Shewanella* sp. strain HN-41. *Proc Natl Acad Sci U S A* **2007**, *104*, 20410-20415, doi:10.1073/pnas.0707595104.
130. Zhao, J.-S.; Manno, D.; Leggiadro, C.; apos; Neil, D.; Hawari, J. *Shewanella halifaxensis* sp. nov., a novel obligately respiratory and denitrifying psychrophile. *International Journal of Systematic and Evolutionary Microbiology* **2006**, *56*, 205-212, doi:https://doi.org/10.1099/ijs.0.63829-0.
131. Li, Z.; Song, F.; Chen, M. Complete Genome Sequence of *Shewanella* sp. Strain Lzh-2, an Algicidal Bacterial Strain Isolated from Lake Taihu, People´s Republic of China. *Microbiology Resource Announcements* **2021**, *10*, 10.1128/mra.00339-00321, doi:doi:10.1128/mra.00339-21.
132. Li, Z.; Lin, S.; Liu, X.; Tan, J.; Pan, J.; Yang, H. A freshwater bacterial strain, *Shewanella* sp. Lzh-2, isolated from Lake Taihu and its two algicidal active substances, hexahydropyrrolo[1,2-a]pyrazine-1,4-dione and 2, 3-indolinedione. *Applied Microbiology and Biotechnology* **2014**, *98*, 4737-4748, doi:10.1007/s00253-014-5602-1.
133. Boles, B.W.; Murdoch, R.W.; Ohlsson, I.; Mikucki, J.A. Draft Genome Sequence of *Shewanella* sp. Strain BF02_Schw, Isolated from Blood Falls, a Feature Where Subglacial Brine Discharges to the Surface of Taylor Glacier, Antarctica. *Microbiology Resource Announcements* **2021**, *10*, 10.1128/mra.01141-01120, doi:doi:10.1128/mra.01141-20.
134. Sravan Kumar, R.; Sasi Jyothsna, T.S.; Sasikala, C.; Seong, C.N.; Lim, C.H.; Park, S.C.; Ramana, C.V. *Shewanella fodinae* sp. nov., isolated from a coal mine and from a marine lagoon. *International Journal of Systematic and Evolutionary Microbiology* **2010**, *60*, 1649-1654, doi:https://doi.org/10.1099/ijs.0.017046-0.

135. Saltikov, C.W.; Cifuentes, A.; Venkateswaran, K.; Newman, D.K. The ars detoxification system is advantageous but not required for As(V) respiration by the genetically tractable *Shewanella species* strain ANA-3. *Appl Environ Microbiol* **2003**, *69*, 2800-2809, doi:10.1128/aem.69.5.2800-2809.2003.
136. Saltikov, C.W.; Wildman, R.A., Jr.; Newman, D.K. Expression dynamics of arsenic respiration and detoxification in *Shewanella* sp. strain ANA-3. *J Bacteriol* **2005**, *187*, 7390-7396, doi:10.1128/jb.187.21.7390-7396.2005.
137. Zargar, K.; Saltikov, C.W. Lysine-91 of the tetraheme c-type cytochrome CymA is essential for quinone interaction and arsenate respiration in *Shewanella* sp. strain ANA-3. *Arch Microbiol* **2009**, *191*, 797-806, doi:10.1007/s00203-009-0511-x.
138. Qasim, M.S.; Lampi, M.; Heinonen, M.-M.K.; Garrido-Zabala, B.; Bamford, D.H.; Käkelä, R.; Roine, E.; Sarin, L.P. Cold-Active *Shewanella glacialimarina* TZS-4T nov. Features a Temperature-Dependent Fatty Acid Profile and Putative Sialic Acid Metabolism. *Frontiers in Microbiology* **2021**, *12*, doi:10.3389/fmicb.2021.737641.
139. Cao, W.-R.; Li, X.; Sun, Y.-Y.; Jiang, M.-Y.; Xu, X.-D.; Li, Y.-J. *Shewanella nanhaiensis* sp. nov., a marine bacterium isolated from sediment of South China Sea, and emended descriptions of *Shewanella woodyi*, *Shewanella hanedai* and *Shewanella canadensis*. *International Journal of Systematic and Evolutionary Microbiology* **2021**, *71*, doi:https://doi.org/10.1099/ijsem.0.005152.
