# Supplementary material for: Novel Insights on Extracellular Electron Transfer Networks in the Desulfovibrionaceae Family: Unveiling the Potential Significance of Horizontal Gene Transfer
Source: Microorganisms. 2024 Aug 29;12(9):1796. doi: 10.3390/microorganisms12091796 (PMC11434368; doi:10.3390/microorganisms12091796)
Supplement: Supplementary file 1 [file microorganisms-12-01796-s001.zip › Tables.zip!/TableS1_Genomic_Features.pdf]

No	Strain	File_name	Genomic information									CheckM results		Phylogenetic information				Origen Source	
			GenBank assembly accession	Genome size (Mbp)	Level	Total number of chromosomes and plasmids	Number of scaffolds	Number of contigs	GC %	Nº sequences CDS-refseq	Nº sequences prot-refseq	Completeness	contamination	Phylum	Family	Gram	Position in Phylogenomic tree	Habitat of Isolation (or found if noted by *)	Type of habitat
32	Desulfocurvibacter africanus PCS	Desulfocurvibacter_africanus_PCS_GCF_000344315.1	GCA_000344315.1	3.929487	Contig	-	-	45	61.2	3554	3497	100.00	0.00	Proteobacteria	Desulfovibrionaceae	Neg.	15	Isolated: Sediment collected from Paleta Creek site in San Diego Bay (hydrocarbon impacted sediments)	Engineered/Impacted system
2	Desulfocurvibacter africanus subsp. africanus DSM 2603	Desulfocurvibacter_africanus_DSM2603_GCF_000422545.1	GCA_000422545.1	4.402525	Scaffold	-	41	44	61	4003	3911	100.00	0.00	Proteobacteria	Desulfovibrionaceae	Neg.	16	Well water from Benghazi, Libya	Freshwater sediments
4	Desulfocurvibacter africanus subsp. africanus str. Walvis Bay	Desulfocurvibacter_africanus_str_Walvis_Bay_GCF_000212675.2	GCA_000212675.2	4.200534	Complete	1	-	-	60.2	3787	3717	100.00	0.00	Proteobacteria	Desulfovibrionaceae	Neg.	17	Habitat: marine mud sample from Walvis Bay, S. W. Africa	Marine water/sediments
12	Desulfocurvus vexinensis DSM 17965	Desulfocurvus_vexinensis_DSM17965_GCF_000519125.1	GCA_000519125.1	3.627185	Scaffold	-	57	61	67.2	3385	3283	99.41	0.00	Proteobacteria	Desulfovibrionaceae	Neg.	12	Isolated: deep artesian well that collected water from a deep saline aquifer used for	Brackish water/sediments
13	Desulfohalovibrio alkalitolerans DSM 16529	Desulfohalovibrio_alkalitolerans_DSM16529_GCF_000422245.1	GCF_000422245.1	3.202328	Contig	-	-	32	64.7	2925	2903	100.00	0.00	Proteobacteria	Desulfovibrionaceae	Neg.	13	Isolated: mild steel coupons from a reactor connected to the return line of the Skanderborg district heating plant (Jutland, Denmark	Engineered/Impacted system
20	Desulfovibrio aminophilus DSM 12254	Desulfovibrio_aminophilus_DSM12254_GCF_000422565.1	GCA_000422565.1	3.419064	Scaffold	-	25	29	66	3270	3231	100.00	0.00	Proteobacteria	Desulfovibrionaceae	Neg.	6	Isolated: anaerobic sludge of a dairy wastewater treatment plant of Santa Fe de Bogota, Colombia	Engineered/Impacted system
23	Desulfovibrio carbinoliphilus subsp. oakridgensis FW-101-2B	Desulfovibrio_carbinoliphilus_FW-101-2B_GCF_000177215.2	GCA_000177215.2	4.221670	Chromosome	3	3	5	66.5	3772	3700	100.00	0.00	Proteobacteria	Desulfovibrionaceae	Neg.	21	Isolated from biostimulated, uranium-contaminated groundwater	Engineered/Impacted system
11	Desulfovibrio cuneatus DSM 11391	Desulfovibrio_cuneatus_DSM11391_GCF_000430005.1	GCA_000430005.1	3.359127	Scaffold	-	76	81	52.7	2774	2715	98.82	0.30	Proteobacteria	Desulfovibrionaceae	Neg.	25	isolated from the littoral sediment of the oligotrophic Lake Stechlin. Obtained from the oxic uppermost layer	Freshwater sediments
43	Desulfovibrio desulfuricans AY5	Desulfovibrio_desulfuricans_AY5_GCF_014843035.1	GCA_014843035.1	3.519154	Contig	-	-	40	57.2	2993	2930	99.41	0.00	Proteobacteria	Desulfovibrionaceae	Neg.	42	Isolated from a faecal sample of a child (11 years old) with Autism Spectrum Disorder	Animal/Human-associated
39	Desulfovibrio desulfuricans DFI.2.30	Desulfovibrio_desulfuricans_DFI.2.30_GCF_020555025.1	GCA_020555025.1	3.648677	Contig	-	-	229	57.1	3379	3195	100.00	0.59	Proteobacteria	Desulfovibrionaceae	Neg.	41	Unknown	Unknown
38	Desulfovibrio desulfuricans DFI.2.72	Desulfovibrio_desulfuricans_DFI.2.72_GCF_020561375.1	GCA_020561375.1	3.664920	Contig	-	-	298	57.1	3491	3229	100.00	1.48	Proteobacteria	Desulfovibrionaceae	Neg.	40	Unknown	Unknown
37	Desulfovibrio desulfuricans DFI.2.73	Desulfovibrio_desulfuricans_DFI.2.73_GCF_020561235.1	GCA_020561235.1	3.693782	Contig	-	-	415	56.9	3642	3294	100.00	0.74	Proteobacteria	Desulfovibrionaceae	Neg.	39	Unknown	Unknown
26	Desulfovibrio desulfuricans DSM 642	Desulfovibrio_desulfuricans_DSM_642_GCF_000420465.1	GCA_000420465.1	3.391683	Contig	-	-	20	57.4	2865	2826	99.41	0.00	Proteobacteria	Desulfovibrionaceae	Neg.	36	Isolated: tar and sand mix around corroded gas main	Engineered/Impacted system
42	Desulfovibrio desulfuricans DSM 7057 (strain G11)	Desulfovibrio_desulfuricans_DSM_7057_GCF_900119095.1	GCA_900119095.1	3.230378	Scaffold	-	142	144	57.3	2961	2731	99.41	0.59	Proteobacteria	Desulfovibrionaceae	Neg.	33	Desulfovibrio sp. strain G11 was isolated from a rumen fluid enrichment	Animal/Human-associated
36	Desulfovibrio desulfuricans Edelweiss	Desulfovibrio_desulfuricans_Edelweiss_GCF_021365555.1	GCA_021365555.1	3.401021	Complete	1	-	-	57.3	2846	2822	100.00	0.00	Proteobacteria	Desulfovibrionaceae	Neg.	38	was isolated from local anoxic creek water in College Station, Tx, USA (Fresh Water Stream/Anoxic Mud)	Freshwater sediments
41	Desulfovibrio desulfuricans IC1	Desulfovibrio_desulfuricans_IC1_GCF_004801255.1	GCA_004801255.1	3.251440	Complete	1	-	-	59.1	2704	2672	99.41	0.39	Proteobacteria	Desulfovibrionaceae	Neg.	34	was isolated with the sulfonate isethionate as the terminal electron acceptor from freshwater marsh mud	Freshwater sediments
35	Desulfovibrio desulfuricans L4	Desulfovibrio_desulfuricans_L4_GCF_017815575.1	GCA_017815575.1	3.554892	Complete	2	-	-	56.81	2966	2896	100.00	0.59	Proteobacteria	Desulfovibrionaceae	Neg.	35	near-bottom sediment slurry from the solid–liquid separation lagoon, of the large-	Engineered/Impacted system
34	Desulfovibrio desulfuricans MCC432	Desulfovibrio_desulfuricans_MCC432_GCF_018785025.1	GCA_018785025.1	3.473822	Scaffold	-	48	51	57.4	2973	2933	100.00	0.00	Proteobacteria	Desulfovibrionaceae	Neg.	37	Unknown	Unknown
40	Desulfovibrio desulfuricans NBRC 13699 (ATCC 7757=NCIB 8372)	Desulfovibrio_desulfuricans_NBRC_13699_GCF_006539305.1	GCA_006539305.1	3.343776	Contig	-	-	26	63.2	2878	2840	99.41	0.00	Proteobacteria	Desulfovibrionaceae	Neg.	30	Unknown	Unknown
29	Desulfovibrio inopinatus DSM 10711	Desulfovibrio_inopinatus_DSM10711_GCF_000429305.1	GCA_000429305.1	5.767550	Scaffold	-	69	80	49.7	4932	4826	100.00	0.60	Proteobacteria	Desulfovibrionaceae	Neg.	18	Habitat: Anoxic marine and brackish sediments	Marine water/sediments
10	Desulfovibrio oxyclinae DSM 11498	Desulfovibrio_oxyclinae_DSM11498_GCF_000375485.1	GCA_000375485.1	3.324581	Contig	-	-	32	66.5	3091	3011	95.27	1.78	Proteobacteria	Desulfovibrionaceae	Neg.	4	Habitat: oxic zone of hypersaline cyanobacterial mats (transferred from Solar Lake, Sinai, Egypt)	Brackish water/sediments
9	Desulfovibrio piger ATCC 29098	Desulfovibrio_piger_ATCC_29098_GCF_000156375.1	GCA_000156375.1	2.867216	Scaffold	-	47	127	67	2598	2476	99.41	0.59	Proteobacteria	Desulfovibrionaceae	Neg.	32	Isolated: human specimens (faeces,peritoneal fuids and intra-abdominal collections)	Animal/Human-associated
19	Desulfovibrio sp. A2	Desulfovibrio_sp_A2_GCF_000226255.1	GCA_000226255.2	4.150685	Contig	-	-	84	67.2	3536	3364	99.41	0.30	Proteobacteria	Desulfovibrionaceae	Neg.	28	Isolated: settling pond of the Chelyabinsk Electrolytic Zinc Smelter in the Urals, Russia	Engineered/Impacted system
18	Desulfovibrio sp. X2	Desulfovibrio_sp_X2_GCF_000422205.1	GCA_000422205.1	3.912295	Contig	-	-	66	67.9	3476	3447	99.41	0.00	Proteobacteria	Desulfovibrionaceae	Neg.	14	Isolated: mid-Chesapeake Bay bottom sediments	Marine water/sediments
33	Desulfovibrio vulgaris DP4	Desulfovibrio_vulgaris_DP4_GCF_000015485.1	GCA_000015485.1	3.661391	Complete	2	-	-	63	3090	3065	99.41	0.00	Proteobacteria	Desulfovibrionaceae	Neg.	31	Isolated: soil at 1 m of depth (Shanxi, China)	Soil
1	Desulfovibrio vulgaris str. Hildenborough (DSM 644, ATCC 29579)	Desulfovibrio_vulgaris_str_Hildenborough_GCF_000195755.1	GCA_000195755.1	3.773159	Complete	2	-	-	63.1	3258	3223	99.41	0.00	Proteobacteria	Desulfovibrionaceae	Neg.	29	Isolation: clay soil near Hildenborough, UK	Soil
28	Fundidesulfovibrio putealis DSM 16056	Fundidesulfovibrio_putealis_DSM16056_GCF_000429325.1	GCA_000429325.1	4.705742	Scaffold	-	29	35	57.8	4233	4173	99.40	0.10	Proteobacteria	Desulfovibrionaceae	Neg.	19	Isolated: water-producing well in France	Freshwater sediments
44	Geobacter anodireducens PheS2	Geobacter_anodireducens_PheS2_GCF_014883105.1	GCA_014883105.1	3.71932	Contig	-	-	61	61.5	3546	3370	99.35	1.51	Proteobacteria	Geobacteraceae	Neg.	60	oil-polluted soil, Beijing, China	Engineered/Impacted system
45	Geobacter argillaceus ATCC BAA-1139	Geobacter_argillaceus_ATCC_BAA-1139_GCF_007830735.1	GCA_007830735.1	4.38531	Scaffold	-	71	72	58.2	4100	3957	100.00	0.00	Proteobacteria	Geobacteraceae	Neg.	48	subsurface kaolin strata in Georgia, USA	Soil
59	Geobacter benzoatilyticus Jerry-YX	Geobacter_sp_Jerry-YX_GCF_017338855.1	GCA_017338855.1	3.5837	Complete	1	-	-	57.6	3287	3220	99.09	0.00	Proteobacteria	Geobacteraceae	Neg.	59	petroleum-contaminated soil in Gansu, China	Engineered/Impacted system
46	Geobacter chapellei DSM 13688	Geobacter_chapellei_DSM13688_GCF_018531195.1	GCA_018531195.1	3.91622	Contig	-	-	60	50.2	3551	3482	99.35	0.00	Proteobacteria	Geobacteraceae	Neg.	46	deep aquifer sediments of the Atlantic Coastal Plain in South Carolina, USA	Brackish water/sediments
47	Geobacter daltonii FRC-32	Geobacter_daltonii_FRC-32_GCF_000022265.1	GCA_000022265.1	4.3045	Complete	1	-	-	53.5	3809	3761	99.35	0.00	Proteobacteria	Geobacteraceae	Neg.	52	Uranium-contaminated subsurface at US (sediments are exposed to mixed waste	Engineered/Impacted system
48	Geobacter grbiciae DSM 13689	Geobacter_grbiciae_DSM13689_GCF_018531165.1	GCA_018531165.1	4.15454	Contig	-	-	63	57.3	3825	3678	98.76	0.00	Proteobacteria	Geobacteraceae	Neg.	57	freshwater aquatic sediment taken from the	Freshwater sediments

49	Geobacter hydrogenophilus DSM 13691	Geobacter_hydrogenophilus_DSM13691_GCF_018501225.1	GCA_018501225.1	4.01553	Contig	-	-	50	58.4	3687	3568	98.10	0.00	Proteobacteria	Geobacteraceae	Neg.	58	A hydrocarbon-contaminated aquifer (SC, Hanahan, Defence Fuel Supply Center, USA)	Engineered/Impacted system
50	Geobacter luticola JCM 17780	Geobacter_luticola_JCM_17780_GCF_018476905.1	GCA_018476905.1	3.66168	Contig	-	-	40	59.7	3418	3355	99.03	0.32	Proteobacteria	Geobacteraceae	Neg.	50	mud of lotus field, Aichi, Japan	Freshwater sediments
51	Geobacter metallireducens GS-15	Geobacter_metallireducens_GS-15_GCF_000012925.1	GCA_000012925.1	4.01118	Complete	2	-	-	59.8	3630	3509	99.42	0.00	Proteobacteria	Geobacteraceae	Neg.	55	Potomac river downstream of Washington, DC, USA	Freshwater sediments
52	Geobacter metallireducens RCH3	Geobacter_metallireducens_RCH3_GCF_000243475.1	GCA_000243475.2	3.7788	Contig	-	-	89	59.8	3471	3373	99.39	0.00	Proteobacteria	Geobacteraceae	Neg.	56	Unknown	Unknown
53	Geobacter pickeringii G13	Geobacter_pickeringii_G13_GCF_000817955.1	GCA_000817955.1	3.6187	Complete	1	-	-	61.4	3334	3250	99.84	0.00	Proteobacteria	Geobacteraceae	Neg.	54	sedimentary kaolin strata, Tabernacle Church, Georgia, USA	Soil
54	Geobacter soli GSS01	Geobacter_soli_GSS01_GCF_000816575.1	GCA_000816575.1	3.65721	Scaffold	-	8	15	61.8	3359	3293	98.67	0.65	Proteobacteria	Geobacteraceae	Neg.	61	soil sample taken from the humic layer (3.5 m beneath the surface) of an underground	Soil
55	Geobacter sp. AOG1	Geobacter_sp_AOG1_GCF_019972315.1	GCA_019972315.1	3.677207	Scaffold	-	1	4	57.4	3295	3263	99.95	0.65	Proteobacteria	Geobacteraceae	Neg.	49	was isolated from paddy soils in Hokkaido using lepidocrocite as electron acceptor	Soil
56	Geobacter sp. AOG2	Geobacter_sp_AOG2_GCF_019972295.1	GCA_019972295.1	3.921258	Scaffold	-	1	5	57.2	3505	3470	99.35	0.65	Proteobacteria	Geobacteraceae	Neg.	43	was isolated from wetland supplemented with magnetite	Freshwater sediments
57	Geobacter sp. DSM 9736	Geobacter_sp_DSM9736_GCF_900187405.1	GCA_900187405.1	3.95778	Chromosome	1	1	2	56.1	3505	3472	99.35	0.65	Proteobacteria	Geobacteraceae	Neg.	53	paddy soil, Vercelli, Italy	Soil
58	Geobacter sp. FeAm09	Geobacter_sp_FeAm09_GCF_008330225.1	GCA_008330225.1	4.09907	Complete	1	-	-	61.1	3886	3703	97.74	1.29	Proteobacteria	Geobacteraceae	Neg.	44	Iron-rich upland soil (moderately acidic unsaturated tropical forest soil in Puerto Rico)	Soil
60	Geobacter sp. OR-1	Geobacter_sp_OR-1_GCF_000813145.1	GCA_000813145.1	4.55427	Contig	-	-	63	54.2	4193	4042	99.33	0.65	Proteobacteria	Geobacteraceae	Neg.	47	arsenic-polluted paddy soil in Japan	Engineered/Impacted system
61	Geobacter sp. SVR	Geobacter_sp_SVR_GCF_016865365.1	GCA_016865365.1	4.71736	Complete	1	-	-	57.9	4341	4222	99.98	2.58	Proteobacteria	Geobacteraceae	Neg.	45	antimony vein of Nakase mine	Soil
62	Geobacter sulfurreducens KN400	Geobacter_sulfurreducens_KN400_GCF_000210155.1	GCA_000210155.1	3.71427	Complete	1	-	-	61.3	3332	3292	99.35	0.00	Proteobacteria	Geobacteraceae	Neg.	65	biofilm formed on an anode poised at -200 mV SHE	Engineered/Impacted system
63	Geobacter sulfurreducens PCA	Geobacter_sulfurreducens_PCA_GCF_000007985.2	GCA_000007985.2	3.81413	Complete	1	-	-	60.9	3436	3367	99.35	0.00	Proteobacteria	Geobacteraceae	Neg.	64	surface sediments of a hydrocarbon-contaminated ditch in Norman, Oklahoma, USA	Engineered/Impacted system
64	Geobacter sulfurreducens YM18	Geobacter_sulfurreducens_YM18_GCF_003574175.1	GCA_003574175.1	3.72641	Complete	1	-	-	60.7	3332	3303	100.00	0.00	Proteobacteria	Geobacteraceae	Neg.	62	Yae river sediment, Miyazaki, Japan ; biofilm formed on an anode poised at 400mV (versus an Ag/AgCl electrode) in a bioelectrochemical	Freshwater sediments
65	Geobacter sulfurreducens YM35	Geobacter_sulfurreducens_YM35_GCF_018511305.1	GCA_018511305.1	3.74522	Complete	1	-	-	60.9	3369	3318	99.35	0.00	Proteobacteria	Geobacteraceae	Neg.	63	Yae river sediment, Miyazaki, Japan	Freshwater sediments
66	Geobacter uraniireducens Rf4	Geobacter_uraniireducens_Rf4_GCF_000016745.1	GCA_000016745.1	5.13636	Complete	1	-	-	54.2	4529	4355	97.66	0.00	Proteobacteria	Geobacteraceae	Neg.	51	in situ uranium bioremediation study site in Rifle, Colorado	Engineered/Impacted system
3	Halodesulfovibrio aestuarii DSM 17919	Halodesulfovibrio_aestuarii_DSM17919_GCF_000384815.1	GCA_000384815.1	3.545807	Contig	-	-	21	45.1	3139	3082	100.00	0.07	Proteobacteria	Desulfovibrionaceae	Neg.	26	Soil (Shoal mud in Germany)	Soil
24	Maridesulfovibrio bastinii DSM 16055	Maridesulfovibrio_bastinii_DSM16055_GCF_000429985.1	GCA_000429985.1	3.909169	Scaffold	-	39	42	44.6	3514	3459	100.00	0.00	Proteobacteria	Desulfovibrionaceae	Neg.	7	Isolated: oilfield production wáter collected from a pipeline of Emeraude Oilfield, Congo	Engineered/Impacted system
16	Maridesulfovibrio frigidus DSM 17176	Maridesulfovibrio_frigidus_DSM17176_GCF_000711735.1	GCA_000711735.1	4.185035	Scaffold	-	24	25	43.3	3716	3645	99.92	0.59	Proteobacteria	Desulfovibrionaceae	Neg.	8	Isolated: permanently cold sediment at the west coast	Marine water/sediments
17	Maridesulfovibrio hydrothermalis DSM 14728	Maridesulfovibrio_hydrothermalis_DSM14728_GCF_000331025.1	GCA_000331025.1	3.708362	Chromosome	2	2	3	47.5	3242	3205	100.00	0.00	Proteobacteria	Desulfovibrionaceae	Neg.	10	Isolated: deep-sea hydrothermal deep-sea hydrothermal chimney sample	Marine water/sediments
15	Maridesulfovibrio salexigens DSM 2638	Maridesulfovibrio_salexigens_DSM2638_GCF_000023445.1	GCA_000023445.1	4.289847	Complete	1	-	-	47	3824	3807	100.00	0.07	Proteobacteria	Desulfovibrionaceae	Neg.	9	Isolated: isolated from "sling mud"* from British Guiana about 1951. Found: Seawater, marine or estuarine muds, pickling brines.	Marine water/sediments
27	Maridesulfovibrio zosterae DSM 11974	Maridesulfovibrio_zosterae_DSM11974_GCF_000425265.1	GCA_000425265.1	4.092653	Scaffold	-	15	20	42.7	3611	3572	100.00	0.00	Proteobacteria	Desulfovibrionaceae	Neg.	11	Isolated: roots of the marine macrophyte Zostera rwarinn	Plant/Algae-associated
8	Megalodesulfovibrio gigas DSM 1382	Megalodesulfovibrio_gigas_DSM1382_GCF_000468495.1	GCA_000468495.1	3.796022	Chromosome	2	2	39	63.5	3358	3285	100.00	0.00	Proteobacteria	Desulfovibrionaceae	Neg.	24	Isolation: sample of water from the Etang de Berre, Marseilles, France	Brackish water/sediments
22	Oleidesulfovibrio alaskensis DSM 16109	Oleidesulfovibrio_alaskensis_DSM_16109_GCF_000482745.1	GCA_000482745.1	3.553960	Scaffold	-	26	31	64.1	3170	3116	100.00	0.00	Proteobacteria	Desulfovibrionaceae	Neg.	27	Isolated: production fluids of offshore oilfields in Alaska	Engineered/Impacted system
30	Paucidesulfovibrio longus DSM 6739	Paucidesulfovibrio_longus_DSM6739_GCF_000420485.1	GCA_000420485.1	3.701832	Scaffold	-	19	20	62.3	3407	3380	100.00	0.20	Proteobacteria	Desulfovibrionaceae	Neg.	5	Isolated: oil-producing well in the Paris Basin	Engineered/Impacted system
5	Pseudodesulfovibrio aespoeensis Aspo-2	Pseudodesulfovibrio_aespoeensis_Aspo-2_GCF_000176915.2	GCA_000176915.2	3.629109	Complete	1	-	-	61.5	3330	3260	99.41	0.00	Proteobacteria	Desulfovibrionaceae	Neg.	2	Isolation: deep subterranean granitic groundwater (600 m) of south-eastern Sweden	Freshwater sediments
31	Pseudodesulfovibrio mercurii ND132	Pseudodesulfovibrio_mercurii_ND132_GCF_000189295.2	GCA_000189295.2	3.858580	Complete	1	-	-	65.2	3481	3452	100.00	0.59	Proteobacteria	Desulfovibrionaceae	Neg.	3	Isolated from estuarine mid-Chesapeake Bay sediments	Brackish water/sediments
21	Pseudodesulfovibrio piezophilus C1TLV30	Pseudodesulfovibrio_piezophilus_C1TLV30_GCF_000341895.1	GCA_000341895.1	3.646098	Chromosome	1	1	2	49.6	3263	3231	100.00	0.00	Proteobacteria	Desulfovibrionaceae	Neg.	1	Isolated: wood falls at a depth of 1693 m in the Mediterranean Sea	Marine water/sediments
96	Shewanella aestuarii JCM 17801	Shewanella_aestuarii_JCM_17801_GCF_014647215.1	GCA_014647215.1	3.884	Contig	-	-	27	41.3	3329	3299	99.14	0.00	Proteobacteria	Shewanellaceae	Neg.	113	tidal flat of Suncheon bay in South Korea	Brackish water/sediments
68	Shewanella algae ATCC 49138	Shewanella_algae_ATCC49138_GCF_019670525.1	GCA_019670525.1	4.84969	Complete	1	-	-	52.5	4262	4130	99.73	1.08	Proteobacteria	Shewanellaceae	Neg.	69	Homo sapiens, Sanofi Diagnostics Pasteur, [illegible]	Animal/Human-associated
69	Shewanella algicola JCM 31091	Shewanella_algicola_JCM_31091_GCF_014648035.1	GCA_014648035.1	4.93824	Contig	-	-	175	42.4	4463	4258	99.63	1.31	Proteobacteria	Shewanellaceae	Neg.	114	marine brown alga (Sargassum thunbergii), coast of Jeju Island, South Korea	Plant/Algae-associated
97	Shewanella algidipiscicola H1	Shewanella_algidipiscicola_H1_GCF_900380485.1	GCA_900380485.1	4.13838	Contig	-	-	22	46.63	3609	3503	99.53	0.22	Proteobacteria	Shewanellaceae	Neg.	92	muddy sediment of the Stora harbor, Skikda, Mediterranean Algerian coast	Marine water/sediments

98	Shewanella amazonensis SB2B	Shewanella_amazonensis_SB2B_GCF_000015245.1	GCA_000015245.1	4.30614	Complete	1	-	-	51.7	3688	3656	100.00	0.54	Proteobacteria	Shewanellaceae	Neg.	75	shallow marine deposits (coastal shelf mud) of the Amazon River delta off of the coast of Brazil	Marine water/sediments
99	Shewanella atlantica HAW-EB5	Shewanella_atlantica_HAW-EB5_GCF_003966265.1	GCA_003966265.1	5.39213	scaffold	-	47	49	46.4	4530	4443	99.84	0.10	Proteobacteria	Shewanellaceae	Neg.	80	marine sediment sampled from the Atlantic Ocean, near Halifax harbour in Canada	Marine water/sediments
84	Shewanella baltica OS678	Shewanella_baltica_OS678_GCF_000178875.2	GCA_000178875.2	5.36877	Complete	2	-	-	46.29	4517	4431	99.84	0.00	Proteobacteria	Shewanellaceae	Neg.	95	Gotland Deep, Baltic Sea	Marine water/sediments
100	Shewanella canadensis HAW-EB2	Shewanella_canadensis_HAW-EB2_GCF_003966225.1	GCA_003966225.1	5.67692	Contig	-	-	60	46.4	4797	4735	99.58	0.04	Proteobacteria	Shewanellaceae	Neg.	81	sediment from Atlantic Ocean	Marine water/sediments
70	Shewanella carassii TUM17387	Shewanella_carassii_TUM17387_GCF_019670705.1	GCA_019670705.1	4.45456	Complete	1	-	-	52.5	3874	3820	99.73	0.54	Proteobacteria	Shewanellaceae	Neg.	70	biopsy specimen of intestinal mucosa from Homo sapiens from Tokyo Japan	Animal/Human-associated
101	Shewanella chilikensis JC5	Shewanella_chilikensis_JC5_GCF_002836945.1	GCA_002836945.1	4.39914	Scaffold	-	114	116	54.6	3927	3820	100.00	0.54	Proteobacteria	Shewanellaceae	Neg.	68	sediment from Chilika Lagoon, near Barkul, Orissa, India	Brackish water/sediments
85	Shewanella denitrificans OS217	Shewanella_denitrificans_OS217_GCF_000013765.1	GCA_000013765.1	4.54591	Complete	1	-	-	46.8	3868	3752	96.01	1.17	Proteobacteria	Shewanellaceae	Neg.	111	Gotland Deep, Baltic Sea, Sweden	Marine water/sediments
86	Shewanella dokdonensis DSM 23626	Shewanella_dokdonensis_DSM_23626_GCF_018394335.1	GCA_018394335.1	4.12741	Complete	1	-	-	50.2	3898	3086	95.61	0.54	Proteobacteria	Shewanellaceae	Neg.	73	seawater from Dokdo Island, South Korea	Marine water/sediments
102	Shewanella donghaensis LT17	Shewanella_donghaensis_LT17_GCF_007567505.1	GCA_007567505.1	4.91277	Complete	1	-	-	38.8	4055	4009	99.78	0.57	Proteobacteria	Shewanellaceae	Neg.	108	deep-sea sediment, Sea of Japan, East Sea, South Korea	Marine water/sediments
103	Shewanella fidelis ATCC BAA-318	Shewanella_fidelis_ATCC_BAA-318_GCF_000518605.1	GCA_000518605.1	4.79869	Scaffold	-	27	35	42.75	4104	4059	99.84	0.00	Proteobacteria	Shewanellaceae	Neg.	86	sediments of the South China Sea	Marine water/sediments
123	Shewanella fodinae KCTC 22506	Shewanella_fodinae_KCTC_22506_GCF_014651955.1	GCA_014651955.1	3.905718	Contig	-	-	50	53.6	3536	3464	99.46	0.54	Proteobacteria	Shewanellaceae	Neg.	72	Coal mine sludge sample collected from a depth of 204.35 m below the surface, mine of Godavarikhani, Karimnagar District, Andhra	Engineered/Impacted system
87	Shewanella frigidimarina NCIMB 400	Shewanella_frigidimarina_NCIMB_400_GCF_000014705.1	GCA_000014705.1	4.84526	Complete	1	-	-	41.58	4053	3980	99.84	0.37	Proteobacteria	Shewanellaceae	Neg.	122	seawater from North Sea near Aberdeen, United Kingdom	Marine water/sediments
71	Shewanella gelidii JCM 30804	Shewanella_gelidii_JCM_30804_GCF_014647855.1	GCA_014647855.1	3.90484	scaffold	-	38	43	47	3405	3343	98.27	0.00	Proteobacteria	Shewanellaceae	Neg.	93	red algae Gelidium amansii, coastal region of Rizhao, PR, China	Plant/Algae-associated
125	Shewanella glacialimarina TZS-4	Shewanella_glacialimarina_TZS-4_GCF_020511155.1	GCA_020511155.1	4.456447	Complete	1	-	-	41.12	3808	3756	98.54	0.79	Proteobacteria	Shewanellaceae	Neg.	112	Baltic Sea ice sample	Marine water/sediments
72	Shewanella glacialipiscicola T147	Shewanella_glacialipiscicola_T147_GCF_019655235.1	GCA_019655235.1	4.10469	Contig	-	-	417	44	3722	3473	99.60	0.63	Proteobacteria	Shewanellaceae	Neg.	97	tissue samples of plaice from Danish waters of the Baltic Sea	Animal/Human-associated
73	Shewanella hafniensis ATCC BAA-1207	Shewanella hafniensis ATCC BAA-1207 GCF 019655335.1	GCA_019655335.1	4.96534	Contig	-	-	300	47	4489	4173	99.84	0.12	Proteobacteria	Shewanellaceae	Neg.	94	marine fish (Cod), Baltic Sea	Animal/Human-associated
120	Shewanella halifaxensis HAW-EB4	Shewanella halifaxensis HAW-EB4 GCF 000019185.1	GCA_000019185.1	5.22692	Complete	1	-	-	44.59	4377	4311	99.84	0.00	Proteobacteria	Shewanellaceae	Neg.	89	sediment in a munitions-dumping area of the	Engineered/Impacted system
118	Shewanella hanedai JCM 20706	Shewanella_hanedai_JCM_20706_GCF_007197645.1	GCA_007197645.1	5.93903	Contig	-	-	86	46.4	4988	4910	99.35	0.71	Proteobacteria	Shewanellaceae	Neg.	79	sediment, Arctic Ocean	Marine water/sediments
104	Shewanella indica KJW27	Shewanella_indica_KJW27_GCF_002836975.1	GCA_002836975.1	4.40281	scaffold	-	55	56	51.2	3862	3795	99.46	0.00	Proteobacteria	Shewanellaceae	Neg.	67	sediment, Laccadive Sea, Indico Ocean	Marine water/sediments
78	Shewanella intestini MCCC 1A01895	Shewanella_intestini_MCCC_1A01895_GCF_018221465.1	GCA_018221465.1	4.00482	scaffold	-	69	71	41.4	3398	3315	98.37	0.00	Proteobacteria	Shewanellaceae	Neg.	110	intestine of adult abalone Haliotis divesicolor, Dadeng Island, Xiamen, coast of Fujian province, China	Animal/Human-associated
105	Shewanella inventionis CGMCC 1.15339	Shewanella inventionis CGMCC 1.15339 GCF 014641855.1	GCA_014641855.1	4.9615	scaffold	-	135	141	43	4374	4199	99.47	1.50	Proteobacteria	Shewanellaceae	Neg.	118	deep-sea sediment, Iheya Ridge in the	Marine water/sediments
88	Shewanella japonica KCTC 22435 (=ATCC: BAA-316)	Shewanella_japonica_KCTC_22435_GCF_002075795.1	GCA_002075795.1	4.97568	Complete	1	-	-	40.8	4141	4049	99.47	0.00	Proteobacteria	Shewanellaceae	Neg.	106	seawater, Gulf of Peter the Great, Russia	Marine water/sediments
74	Shewanella khirikhana TH2012	Shewanella_khirikhana_TH2012_GCF_003957745.1	GCA_003957745.1	4.859	Complete	2	-	-	54.81	4177	4103	100.00	0.62	Proteobacteria	Shewanellaceae	Neg.	74	hepatopancreas of EMS/AHPND-diseased shrimp (Penaeus (Litopenaeus) vannamei), Chanthaburi, Thailand	Animal/Human-associated
89						-		104		3981	3879	99.33	0.00	Proteobacteria	Shewanellaceae	Neg.	116		
	Shewanella litoralis JCM 32306	Shewanella litoralis JCM 32306 GCF 014648335.1	GCA 014648335.1	4.60151	scaffold		101		43.7									seawater, Ulleung island, South Korea	Marine water/sediments
106	Shewanella litorisediminis SMK1-12	Shewanella litorisediminis SMK1-12 GCF 016834455.1	GCA_016834455.1	4.23777	Complete	1	-	-	58	3655	3607	100.00	1.08	Proteobacteria	Shewanellaceae	Neg.	76	it was isolated from a tidal flat sediment on	Brackish water/sediments
90	Shewanella livingstonensis LMG 19866	Shewanella_livingstonensis_LMG_19866_GCF_003855395.1	GCA_003855395.1	4.83988	Complete	1	-	-	41	4109	3992	99.53	0.67	Proteobacteria	Shewanellaceae	Neg.	121	seawater, Livingstone Island, Antarctica	Marine water/sediments
107	Shewanella mangrovi YQH10	Shewanella_mangrovi_YQH10_GCF_000753795.1	GCA_000753795.1	4.21579	Contig	-	-	49	48.3	3725	3683	99.46	0.54	Proteobacteria	Shewanellaceae	Neg.	71	mangrove sediment, Zhangzhou, China	Brackish water/sediments
91	Shewanella marina JCM 15074	Shewanella_marina_JCM_15074_GCF_000614975.1	GCA_000614975.1	4.42465	Contig	-	-	75	40.8	4039	3579	99.16	1.08	Proteobacteria	Shewanellaceae	Neg.	66	Seawater near Gwangyang, South Korea	Marine water/sediments
108	Shewanella marisflavi EP1	Shewanella_marisflavi_EP1_GCF_002215585.1	GCA_002215585.1	4.32145	Complete	1	-	-	50	3689	3645	99.76	0.06	Proteobacteria	Shewanellaceae	Neg.	91	marine sediments in Xiamen, China	Marine water/sediments
92	Shewanella maritima D4-2	Shewanella_maritima_D4-2_GCF_004295345.1	GCA_004295345.1	4.71801	Complete	1	-	-	44.5	3912	3739	98.85	0.74	Proteobacteria	Shewanellaceae	Neg.	109	seawater surface, Dokdo Island, South Korea	Marine water/sediments
75	Shewanella morhuae ATCC BAA-1205	Shewanella_morhuae_ATCC_BAA-1205_GCF_900156405.1	GCA_900156405.1	4.19037	scaffold	-	32	42	44	3583	3465	99.29	0.04	Proteobacteria	Shewanellaceae	Neg.	98	marine fish (cod), Baltic Sea	Animal/Human-associated
126	Shewanella nanhaiensis NR704-98	Shewanella_nanhaiensis_NR704-98_GCF_019457885.1	GCA_019457885.1	5.683156	Scaffold	-	103	105	43.9	4857	4625	99.84	0.00	Proteobacteria	Shewanellaceae	Neg.	77	marine sediment of the northern South China Sea	Marine water/sediments
109	Shewanella oneidensis MR-1	Shewanella_oneidensis_MR-1_GCF_000146165.2	GCA_000146165.2	5.13142	Complete	2	-	-	46	4510	4202	99.84	0.04	Proteobacteria	Shewanellaceae	Neg.	104	sediment from Oneida lake in New York, USA	Freshwater sediments
76	Shewanella pealeana ATCC 700345	Shewanella_pealeana_ATCC_700345_GCF_000018285.1	GCA_000018285.1	5.17458	Complete	1	-	-	45	4290	4194	99.68	0.04	Proteobacteria	Shewanellaceae	Neg.	88	accesory nidamental gland (ANG) from a female squid (Loligo pealei), Marine Biological	Animal/Human-associated
110	Shewanella piezotolerans WP3	Shewanella_piezotolerans_WP3_GCF_000014885.1	GCA_000014885.1	5.39648	Complete	1	-	-	43.29	4534	4404	99.84	0.00	Proteobacteria	Shewanellaceae	Neg.	85	sediment, west Pacific Ocean	Marine water/sediments

77	Shewanella polaris SM1901	Shewanella_polaris_SM1901_GCF_006385555.1	GCA_006385555.1	4.64854	Complete	1	-	-	40.5	3956	3862	99.19	1.03	Proteobacteria	Shewanellaceae	Neg.	119	brown algae, Kings Bay, Svalbard, Arctic Ocean	Plant/Algae-associated
111	Shewanella psychrophila WP2	Shewanella_psychrophila_WP2_GCF_002005305.1	GCA_002005305.1	6.35341	Complete	1	-	-	44.3	5468	5238	99.47	1.34	Proteobacteria	Shewanellaceae	Neg.	83	marine sediment, Pacific Ocean	Marine water/sediments
67	Shewanella putrefaciens ATCC 8071	Shewanella_putrefaciens_ATCC_8071_GCF_016406325.1	GCA_016406325.1	4.38633	Complete	1	-	-	43.8	3776	3713	99.65	0.04	Proteobacteria	Shewanellaceae	Neg.	100	Butter with surface taint, England	Food
80	Shewanella sairae JCM 11563	Shewanella_sairae_JCM_11563_GCF_019655015.1	GCA_019655015.1	5.55973	Contig	-	-	702	43	5503	4843	99.68	1.49	Proteobacteria	Shewanellaceae	Neg.	87	intestine of a Pacific saury, Pacific Ocean, off Japan	Animal/Human-associated
93	Shewanella saliphila JCM 32304	Shewanella_saliphila_JCM_32304_GCF_014648295.1	GCA_014648295.1	4.62135	scaffold	-	126	128	43.4	4040	3920	99.34	0.31	Proteobacteria	Shewanellaceae	Neg.	115	seawater, Ulleung island, South Korea	Marine water/sediments
81	Shewanella schlegeliana JCM 11561	Shewanella_schlegeliana_JCM_11561_GCF_016765635.1	GCA_016765635.1	4.8729	scaffold	-	29	31	45	4143	3977	99.76	0.00	Proteobacteria	Shewanellaceae	Neg.	90	Black porgy's intestine, Japan	Animal/Human-associated
112	Shewanella sediminis HAW-EB3	Shewanella_sediminis_HAW-EB3_GCF_000018025.1	GCA_000018025.1	5.51767	Complete	1	-	-	46.08	4588	4538	99.84	0.00	Proteobacteria	Shewanellaceae	Neg.	82	Marine sediment of Emerald Basin in 215 meter deep, Canada	Marine water/sediments
124	Shewanella sp. ANA-3	Shewanella_sp_ANA-3_GCF_000203935.1	GCA_000203935.1	5.25115	Complete	2	-	-	47.93	4479	4373	99.84	0.04	Proteobacteria	Shewanellaceae	Neg.	102	isolated from a wooden pier within a brackish estuarine environment at Woods Hole	Brackish water/sediments
113	Shewanella sp. Arc9-LZ	Shewanella_sp_Arc9-LZ_GCF_010092445.1	GCA_010092445.1	4.91103	Complete	1	-	-	41.61	4085	4041	99.84	1.47	Proteobacteria	Shewanellaceae	Neg.	123	marine sediment, Arctic Ocean	Marine water/sediments
122	Shewanella sp. BF02_Schw	Shewanella_sp_BF02_Schw_GCF_014596695.2	GCA_014596695.2	5.30424	Contig	-	-	29	41.43	4650	4420	99.84	2.65	Proteobacteria	Shewanellaceae	Neg.	124	Subglacial Brine, Blood Falls, Taylor Glacier,	Brackish water/sediments
119	Shewanella sp. HN-41	Shewanella_sp_HN-41_GCF_000217915.1	GCA_000217915.2	4.41403	Contig	-	-	47	46.53	3859	3653	96.06	0.29	Proteobacteria	Shewanellaceae	Neg.	101	rock fragments of black oil shale from tidal flats in Haenam, Korea	Brackish water/sediments
121	Shewanella sp. LZH-2	Shewanella_sp_LZH-2_GCF_016859145.1	GCA_016859145.1	4.63453	Complete	1	-	-	46.31	3942	3900	99.84	0.15	Proteobacteria	Shewanellaceae	Neg.	105	Surface water samples collected from Meiliang Bay of Lake Taihu in China	Freshwater sediments
114	Shewanella sp. M16	Shewanella_sp_M16_GCF_018224605.1	GCA_018224605.1	5.28432	Contig	-	-	180	45	4721	4531	99.84	0.48	Proteobacteria	Shewanellaceae	Neg.	96	arsenic-containing biofilms (bottom sediments and microbial mats of the Gertruda Adit (gold	Engineered/Impacted system
82	Shewanella sp. MR-4	Shewanella_sp_MR-4_GCF_000014685.1	GCA_000014685.1	4.70629	Complete	1	-	-	47.89	3963	3928	99.84	0.00	Proteobacteria	Shewanellaceae	Neg.	103	seawater, Black Sea	Marine water/sediments
83	Shewanella sp. TC10	Shewanella_sp_TC10_GCF_009663145.1	GCA_009663145.1	4.99057	Contig	-	-	129	40.47	4303	4095	99.59	0.97	Proteobacteria	Shewanellaceae	Neg.	107	Marine biofilm from an immersion site in Toulon bay, France	Marine water/sediments
115	Shewanella sp. W3-18-1	Shewanella_sp_W3-18-1_GCF_000015185.1	GCA_000015185.1	4.70838	Complete	1	-	-	44.63	4116	4021	99.94	0.00	Proteobacteria	Shewanellaceae	Neg.	99	Marine sediments from Pacific Ocean	Marine water/sediments
94	Shewanella ulleungensis JCM 32305	Shewanella_ulleungensis_JCM_32305_GCF_014648315.1	GCA_014648315.1	4.63752	Contig	-	-	44	42.1	3979	3892	99.76	7.72	Proteobacteria	Shewanellaceae	Neg.	117	surface seawater, Ulleung Island, South Korea	Marine water/sediments
116	Shewanella vesiculosa LMG 24424	Shewanella_vesiculosa_LMG_24424_GCF_003797885.1	GCA_003797885.1	4.72264	scaffold	-	45	46	42	3987	3929	98.90	0.88	Proteobacteria	Shewanellaceae	Neg.	120	coastal marine sediments, Shetland Islands, Antarctica	Marine water/sediments
117	Shewanella violacea DSS12	Shewanella_violacea_DSS12_GCF_000091325.1	GCA_000091325.1	4.9621	Complete	1	-	-	44.72	4013	3892	99.42	1.57	Proteobacteria	Shewanellaceae	Neg.	84	mud, Ryukyu Trench, Philippine Sea	Marine water/sediments
95	Shewanella woodyi ATCC 51908	Shewanella_woodyi_ATCC_51908_GCF_000019525.1	GCA_000019525.1	5.9354	Complete	1	-	-	39	4938	4859	99.84	0.00	Proteobacteria	Shewanellaceae	Neg.	78	Detritus in seawater (also squid ink); Alboran Sea	Marine water/sediments
25	Solidesulfovibrio alcoholivorans DSM 5433	Solidesulfovibrio_alcoholivorans_DSM5433_GCF_000702665.1	GCA_000702665.1	5.128906	Contig	-	-	65	64.5	4687	4393	100.00	2.98	Proteobacteria	Desulfovibrionaceae	Neg.	23	Isolated: anaerobic industrial fermenter fed with waste water from the alcohol industry	Engineered/Impacted system
14	Solidesulfovibrio fructosivorans JJ	Solidesulfovibrio_fructosivorans_JJ_GCF_000179555.1	GCA_000179555.1	4.673595	Contig	-	-	75	64.13	4261	4167	100.00	0.00	Proteobacteria	Desulfovibrionaceae	Neg.	22	isolated: estuarine sediment	Brackish water/sediments
7	Solidesulfovibrio magneticus RS-1	Solidesulfovibrio_magneticus_RS-1_GCF_000010665.1	GCA_000010665.1	5.31562	Complete	3	-	-	65.8	4672	4548	100.00	0.00	Proteobacteria	Desulfovibrionaceae	Neg.	20	Isolation: freshwater sediments from the Kameno River, Wakayama Prefecture, Western Japan	Freshwater sediments
