# Supplementary material for: Novel Insights on Extracellular Electron Transfer Networks in the Desulfovibrionaceae Family: Unveiling the Potential Significance of Horizontal Gene Transfer
Source: Microorganisms. 2024 Aug 29;12(9):1796. doi: 10.3390/microorganisms12091796 (PMC11434368; doi:10.3390/microorganisms12091796)
Supplement: Supplementary file 1 [file microorganisms-12-01796-s001.zip › Tables.zip!/TableS3_Growth_conditions.pdf]

No	Strain	pH Optimum	Temp. Optimum (°C)	Classification	Optimal Salinity (%)	Salinity tolerance - Category	Trophic level	Oxygen tolerance	Oxidation	Electron donors for growth	Electron Acceptor for growth (other than SO4)	Fe(III) reduction?	g (doubling time)
1	Desulfovibrio vulgaris str. Hildenborough (DSM 644, ATCC 29579)	7	30	Mesophile	nd	nd	Chemolithotroph	Anaerobe	I	lactate, pyruvate, formate	sulfate	Y	8.38
2	Desulfocurvibacter africanus subsp. africanus DSM 2603	7	37	Mesophile	nd	Low	nd	Anaerobe	I	Lactate, pyruvate, ethanol, formate and hydrogen	sulfite and thiosulfate	nd	4
3	Halodesulfovibrio aestuarii DSM 17919	7.5	30	Mesophile	2	High	nd	Facultative anaerobe	I	acetate, benzoate, aspartate, glucose, lactose, galactose, formate, fumarate, succinate, starch, malate, lactate, pyruvate, sulfite	sulfate	nd	6.33
4	Desulfocurvibacter africanus subsp. africanus str. Walvis Bay	7	37	Mesophile	nd	High	nd	Facultative anaerobe	I	lactate, malate, pyruvate, and ethyl alcohol	sulfate	nd	4
5	Pseudodesulfovibrio aespoeensis Aspo-2	7.5	25 - 30	Mesophile	0.7	Medium	Lithoheterotroph	Anaerobe	I	formate, succinate, methanol, fumarate, malate, 2-propanol, isobutanol, alanine, butyrate, phenol, benzoate, palmitate, ethanol and acetate	sulfate, thiosulfate and sulfur.	N	5.87
7	Solidesulfovibrio magneticus RS-1	7	30	Mesophile	0.5	Low	Chemoorganotroph	Facultative anaerobe	I	lactate, pyruvate, malate, oxalo-acetate and glycerol	sulfate, thiosulfate and fumarate	nd	9.3
8	Megalodesulfovibrio gigas DSM 1382	nd	30 - 35	Mesophile	nd	Medium	nd	Facultative anaerobe	I	Lactate and pyruvate	sulfate	nd	6.7
9	Desulfovibrio piger ATCC 29098	nd	37	Mesophile	nd	Low	nd	Facultative anaerobe	I	pyruvate, ethanol and hydrogen	sulfate	nd	11
10	Desulfovibrio oxyclinae DSM 11498	7	35	Mesophile	5 - 10	High	Chemolithotroph	Anaerobe	I	hydrogen, lactate, sulfide, and sulfite	oxygen, sulfate, sulfite, thiosulfate, sulfur, and fumarate	nd	13
11	Desulfovibrio cuneatus DSM 11391	7.2	28	Psychrotolerant	nd	Low	nd	Anaerobe	I	H_2, formate, pyruvate, lactate, malate, fumarate, sulfide and sulfite	Sulfate, sulfite, thiosulfate, and elemental sulfur	nd	18
12	Desulfocurvus vexinensis DSM 17965	6.9	37	Mesophile	0.2	Medium	Chemoorganotroph	Anaerobe	I	Lactate, formate and pyruvate	Sulfate, sulfite and thiosulfate	nd	3.3
13	Desulfohalovibrio alkalitolerans DSM 16529	9.0 – 9.4	43	Mesophile	0.13	Low	nd	Anaerobe	I	lactate, pyruvate, formate and hydrogen / acetate	sulphate, sulphite and thiosulphate	N	4.6
14	Solidesulfovibrio fructosivorans JJ	6.5 - 7	35	Mesophile	0	High	nd	Facultative anaerobe	I	formate, lactate, pyruvate, glycerol, fumarate, malate and fructose	Elemental sulfur, sulfate, sulfite and thiosulfate	nd	11.5
15	Maridesulfovibrio salexigens DSM 2638	6.8	30	Mesophile	nd	High	nd	Anaerobe	I	Lactate, pyruvate, formate, malate	Sulfate	nd	nd
16	Maridesulfovibrio frigidus DSM 17176	7.1	20 - 23	Psychrotolerant	2 - 3	High	nd	Anaerobe	I	Lactate, formate, hydrogen, ethanol, fumarate and alanine	Sulfate and sulfite	Y	nd
17	Maridesulfovibrio hydrothermalis DSM 14728	7.8	35	Mesophile	2.5	High	Hydrogenotrophic	Facultative anaerobe	I	H_2/CO_2, lactate, formate, ethanol, choline and glycerol	Sulfate, thiosulfate and sulfite	nd	11.34
18	Desulfovibrio sp. X2	nd	nd	Mesophile	nd	High	nd	Anaerobe	nd	nd	nd	nd	nd
19	Desulfovibrio sp. A2	7.3	28	Mesophile	nd	nd	nd	Anaerobe	I	lactate, malate, fumarate, succinate,citrate, formate, ethanol, and alanine	sulfate	nd	2.55
20	Desulfovibrio aminophilus DSM 12254	7.5	35	Mesophile	0.05 - 0.75	Medium	Chemoorganotroph	Facultative anaerobe	I	Formate, alanine, aspartate, leucine, isoleucine, valine, and methionine, H_2/CO_2 and ethanol	Sulfate, sulfite, and thiosulfate	nd	nd
21	Pseudodesulfovibrio piezophilus C1TLV30	7.3	30	Mesophile	2.5	High	nd	Facultative anaerobe	I	lactate, fumarate, formate, malate, pyruvate and ethanol	sulfate, thiosulfate and sulfite	nd	10
22	Oleidesulfovibrio alaskensis DSM 16109	7	37	Mesophile	2.5	High	nd	Facultative anaerobe	I	lactate, pyruvate and succinate	sulphate, sulphite and thiosulphate	nd	5.2
23	Desulfovibrio carbinoliphilus subsp. oakridgensis FW-101-2B	nd	37	Mesophile	nd	nd	nd	Anaerobe	nd	nd	sulfate	nd	nd
24	Maridesulfovibrio bastinii DSM 16055	5.8 – 6.2	35 - 40	Mesophile	4	High	nd	Facultative anaerobe	I	Pyruvate, Ethanol, Butanol, Glycerol	Sulfate, sulfite, thiosulfate and elemental sulfur	nd	15

25	Solidesulfovibrio alcoholivorans DSM 5433	7	35 - 37	Mesophile	0.75	Medium	nd	Facultative anaerobe	I	hydrogen, formate, lactate, pyruvate, fumarate, malate, succinate, DHA, glycerol, 1,2-propanediol, 1,3-propanediol, 1,4-butanediol, Pentanol-I, butanol-I , propanol-I and etanol	Sulfur, sulfate, sulfite and thiosulfate	nd	nd
26	Desulfovibrio desulfuricans DSM 642	7	37	Mesophile	nd	nd	nd	Anaerobe	I	lactate	sulfate	Y	nd
27	Maridesulfovibrio zosterae DSM 11974	6.8 - 7.3	32.5 - 34.5	Mesophile	1.17	High	Lithotroph / Diazotroph	Anaerobe	I	pyruvate, lactate, H_2 + acetate + CO_2, Formate + acetate, fumarate, malate, alanine, choline, ethanol	Sulfate, thiosulfate, sulfite and elemental sulfur	nd	6.93
28	Fundidesulfovibrio putealis DSM 16056	7	30	Mesophile	0.1	Low	nd	Facultative anaerobe	I	H_2, lactate, pyruvate, malate, fumarate, ethanol, butanol and glycine	sulfate, sulfite and thiosulfate	nd	6.02
29	Desulfovibrio inopinatus DSM 10711	6.9 - 7.2	30	Mesophile	1	Medium	Chemoheterotroph	Facultative anaerobe	I	$H_{2,}$ formate, Ethanol, lactate, pyruvate, malate, fumarate, hydroxyhydroquinone, fructose, and ribose	sulfate, thiosulfate, or sulfite	nd	20
30	Paucidesulfovibrio longus DSM 6739	7.4	35	Mesophile	1 - 2	High	nd	Facultative anaerobe	I	H_2, lactate, pyruvate, and formate	sulfate, sulfite, thiosulfate, and elemental sulfur	nd	3
31	Pseudodesulfovibrio mercurii ND132	7.8	32	Mesophile	2	Medium	nd	Anaerobe	I	Lactate, pyruvate, fumaate	sulfate and sulfite	N	6.92
32	Desulfocurvibacter africanus PCS	7	37	Mesophile	nd	nd	nd	Anaerobe	I	lactate, pyruvate, benzoate, succinate, galactose, trehalose, sucrose, glucose, fumarate	Sulfate, thiosulfate, elemental sulfur, iron(III), cromium (VI)	Y	22.21
33	Desulfovibrio vulgaris DP4	nd	nd	Mesophile	nd	nd	nd	Anaerobe	I	lactate	sulfate	nd	9
34	Desulfovibrio desulfuricans MCC432	nd	nd	nd	nd	nd	nd	Anaerobe	nd	nd	nd	nd	nd
35	Desulfovibrio desulfuricans L4	nd	nd	nd	nd	nd	nd	Anaerobe	nd	nd	nd	nd	nd
36	Desulfovibrio desulfuricans Edelweiss	nd	nd	nd	nd	Low	nd	Anaerobe	nd	nd	nd	nd	nd
37	Desulfovibrio desulfuricans DFI.2.73	nd	nd	nd	nd	nd	nd	Anaerobe	nd	nd	nd	nd	nd
38	Desulfovibrio desulfuricans DFI.2.72	nd	nd	nd	nd	nd	nd	Anaerobe	nd	nd	nd	nd	nd
39	Desulfovibrio desulfuricans DFI.2.30	nd	nd	nd	nd	nd	nd	Anaerobe	nd	nd	nd	nd	nd
40	Desulfovibrio desulfuricans NBRC 13699 (ATCC 7757=NCIB 8372)	nd	nd	nd	nd	nd	nd	Anaerobe	nd	nd	nd	nd	nd
41	Desulfovibrio desulfuricans IC1	nd	28	Mesophile	nd	Low	nd	Facultative anaerobe	I	ethanol, H2, formate (+ acetate), lactate, malate, pyruvate	sulfate, fumarate, nitrate, sulfate, sulfite and thiosulfate	nd	8.5 - 9.5
42	Desulfovibrio desulfuricans DSM 7057 (strain G11)	nd	37	Mesophile	nd	Low	nd	Anaerobe	I	lactate, ethanol, formate, and H2	sulfate	nd	nd
43	Desulfovibrio desulfuricans AY5	nd	nd	Mesophile	nd	Low	nd	Anaerobe	I	lactate, pyruvate, fumarate, ethanol, glycerol, and choline	sulfate	nd	nd
44	Geobacter anodireducens PheS2	nd	nd	Mesophile	nd	nd	nd	Anaerobe	nd	nd	nd	nd	nd
45	Geobacter argillaceus ATCC BAA-1139	6.2 - 6.8	30	Mesophile	nd	nd	nd	Anaerobe	C	ethanol. butanol, glycerol, acetate, lactate, butyrate, pyruvate, valerate	PCFO, ferric citrate, Fe(III)-NTA, Fe(III) pyrophosphate, MnOOH, elemental sulfur, nitrate, U(VI)	Y	nd
46	Geobacter chapellei DSM 13688	nd	25	Mesophile	nd	Medium	Chemoorganotroph	Facultative anaerobe	C	acetate, formate, ethanol, lactate	Fe(III), fumarate, Mn(IV), U(VI), AQDS	Y	nd
47	Geobacter daltonii FRC-32	6.7 - 7.3	30	Mesophile	0	Low	Chemolithotroph	Anaerobe	C	acetate, formate, butanol and butyrate, benzoate and toluene	U(VI), Fe(III), Fe(III)-oxyhydroxide, Ferric citrate, elemental sulfur, malate and fumarate.	Y	nd
48	Geobacter grbiciae DSM 13689	nd	30	Mesophile	nd	Low	Chemoorganotroph	Facultative anaerobe	C	acetate, H2, formate, propionate, ethanol, benzoate, toluene, pyruvate, butyrate	fumarate, Fe(III), AQDS	Y	nd
49	Geobacter hydrogenophilus DSM 13691	6.5	35	Mesophile	0	Low	Chemoorganotroph	Facultative anaerobe	C	H2, formate, acetate, propionate, ethanol, benzoate, butyrate, pyruvate, succinate	Fe(III), fumarate, U(VI), S(0), AQDS	Y	nd
50	Geobacter luticola JCM 17780	6.5 - 7.5	30 - 37	Mesophile	0	Low	nd	Facultative anaerobe	C	acetate, lactate, pyruvate, succinate	Fe(III)-NTA, ferric citrate, amorphous iron (III) hydroxide, nitrate	Y	nd
51	Geobacter metallireducens GS-15	6.7 - 7	30 - 35	Mesophile	nd	Low	nd	Facultative anaerobe	C	H2, acetate, benzaldehyde, benzoate, benzylalcohol, butanol, butyrate, p-cresol, ethanol, p-hydroxybenzaldehyde, p-hydroxybenzoate, p-hydroxybenzylalcohol, isobutyrate, isovalerate, phenol, propionate, propanol, pyruvate, toluene and valerate	Fe(III) oxide, Mn(IV) oxide, nitrate	Y	nd

52	Geobacter metallireducens RCH3	nd	nd	Mesophile	nd	nd	nd	Anaerobe	nd	nd	nd	nd	nd
53	Geobacter pickeringii G13	6.6 - 7.2	30	Mesophile	nd	nd	nd	Anaerobe	C	lactate, acetate, ethanol, butanol, glycerol, butyrate, pyruvate, valerate, methanol, succinate	ferric-citrate, PCFO, Fe(III)-NTA, Fe(III)-pyrophosphate, MnOOH, S(0), AQDS, fumarate, malate, U(VI)	Y	nd
54	Geobacter soli GSS01	7	30	Mesophile	nd	Medium	nd	Facultative anaerobe	C	acetate, ethanol, glucose, butyrate, pyruvate, benzoate, benzaldehyde, m-cresol and phenol	insoluble Fe(III) oxides, ferrihydrite, Fe(III) citrate, Mn (IV), sulfur and 2, 6-anthraquinone-disulphonate (AQDS)	Y	nd
55	Geobacter sp. AOG1	nd	nd	nd	nd	nd	nd	Anaerobe	C	acetate	Fe(III)-NTA	Y	nd
56	Geobacter sp. AOG2	nd	nd	nd	nd	Low	nd	Anaerobe	C	acetate	Fe(III)-NTA	Y	nd
57	Geobacter sp. DSM 9736	nd	30	Mesophile	nd	nd	nd	Anaerobe	nd	nd	nd	nd	nd
58	Geobacter sp. FeAm09	5	37	Mesophile	nd	nd	Autotrophic	Anaerobe	C	acetate, fumarate, H2, ethanol	Fe(III)-nitrilotriacetic acid, soluble Fe(III), synthetic ferrihydrite	Y	nd
59	Geobacter benzoatilyticus Jerry-YX	7	30	Mesophile	nd	Low	nd	Facultative anaerobe	C	ethanol, acetate, benzoate	fumarate, ferric citrate, ferrihydrite	Y	nd
60	Geobacter sp. OR-1	nd	nd	Mesophile	nd	nd	nd	Anaerobe	C	acetate, formate, lactate	arsenate, fumarate, MnO2, Fe(III) citrate, soluble Fe (III), ferrihydrite, nitrate, Fe(III)-NTA, nitrite, selenate, malate, thiosulfate	Y	nd
61	Geobacter sp. SVR	nd	nd	Mesophile	nd	nd	nd	Anaerobe	C	acetate, lactate, pyruvate, formate	antimony [Sb(V)], Fe(III)-citrate, AQDS, and nitrate	Y	nd
62	Geobacter sulfurreducens KN400	nd	nd	Mesophile	nd	nd	Chemoorganotroph	Facultative anaerobe	C	acetate	fumarate	Y	nd
63	Geobacter sulfurreducens PCA	nd	30 - 35	Mesophile	nd	Medium	Chemoorganotroph	Facultative anaerobe	C	acetate, H2	fumarate, S(0), Fe(III) oxides; ferric PP1, ferric oxyhydroxide, ferric citrate, Co(III)-EDTA, or malate	Y	nd
64	Geobacter sulfurreducens YM18	nd	30	Mesophile	nd	Low	nd	Anaerobe	C	acetate	fumarate, Fe (III) oxide, graphite anode electrodes	Y	nd
65	Geobacter sulfurreducens YM35	nd	30	Mesophile	nd	Low	nd	Anaerobe	C	acetate	fumarate	nd	nd
66	Geobacter uraniireducens Rf4	6.5 - 7	32	Mesophile	nd	nd	nd	Anaerobe	C	acetate, lactate, pyruvate and ethanol	Fe(III), U(VI), Mn(IV), anthraquinone-2,6-disulfonate, malate and fumarate. poorly crystalline Fe(III) oxide, ferruginous smectite SWa-1, Fe(III) nitrilotriacetate, Fe(III) pyrophosphate, birnessite (MnOOH), AQDS, malate and fumarate.	Y	nd
67	Shewanella putrefaciens ATCC 8071	nd	nd	Mesophile	nd	High	nd	Facultative anaerobe	nd	nd	TMAO, ferric ions, nitrite, thiosulfate, sulfite	Y	nd
68	Shewanella algae ATCC 49138	nd	nd	Mesophile	nd	Low	nd	Aerobe	nd	nd	nd	nd	nd
69	Shewanella algicola JCM 31091	7.5	20	Psychrotolerant	nd	High	nd	Aerobe	I	cellobiose, D-gluconate, D-mannitol, D-tagatose, D-xylose, L-alanylglycine, L-ornithine, L-	nitrate	nd	nd
70	Shewanella carassii TUM17387	nd	35	Mesophile	nd	Low	nd	Facultative anaerobe	nd	nd	nitrate	nd	nd
71	Shewanella gelidii JCM 30804	7	24 - 28	Mesophile	2 - 3	High	nd	Facultative anaerobe	nd	nd	nitrate	nd	nd
72	Shewanella glacialipiscicola T147	nd	25	Mesophile	nd	Medium	Chemoheterotroph	Aerobe	I	lactate, gluconate, N-acetylglucosamine and malate	nitrate, TMAO	nd	nd

73	Shewanella hafniensis ATCC BAA-1207	7.0	25	Mesophile	nd	High	Chemoheterotroph	Aerobe	I	D-Glucose, gluconate, lactate, maltose, N-acetylglucosamine, malate and citrate	nitrate, TMAO	nd	nd
74	Shewanella khirikhana TH2012	7 - 7.5	30	Mesophile	1.5 - 2	High	nd	Facultative anaerobe	nd	nd	nitrate	nd	nd
75	Shewanella morhuae ATCC BAA-1205	7	25	Mesophile	nd	Medium	Chemoheterotroph	Aerobe	I	gluconate, N-acetylglucosamine and malate	nitrate, TMAO	nd	nd
76	Shewanella pealeana ATCC 700345	7	25 - 30	Mesophile	2.9	High	nd	Facultative anaerobe	C	Glucose, galactose, lactate, acetate, pyruvate, citrate, succinate, glutamate, Casamino acids, yeast extract and peptone (aerobically). Lactate	elemental sulfur, nitrate, fumarate, Fe, Mn, TMAO, thiosulfate, O2	Y	nd
77	Shewanella polaris SM1901	7.5	15 - 20	Psychrotolerant	1.5	High	nd	Facultative anaerobe	I	d-glucose, d-mannitol, maltose and malic acid	nitrate	nd	nd
78	Shewanella intestini MCCC 1A01895	7 - 8	nd	Mesophile	5	High	nd	Anaerobe	I	amygdalin, arabinose, mannitol, melibiose, myo-inositol, rhamnose, sorbitol, sucrose	nitrate	nd	nd
80	Shewanella sairae JCM 11563	7 - 8	20 - 25	Psychrotolerant	2 - 3	High	nd	Aerobe	I	D-glucose, pyruvate, propionate, D-glucosamine, N-acetylglucosamine, D-ribose, valerate, L-alanine, L-arginine, L-asparagine, L-glutamine, L-glutamate, L-isoleucine, L-serine and Lthreonine.	O2, nitrate, TMAO	nd	nd
81	Shewanella schlegeliana JCM 11561	7 - 8	20 - 25	Psychrotolerant	2 - 3	High	Chemoheterotroph	Facultative anaerobe	I	D-glucose, pyruvate, propionate, D-glucosamine, Nacetylglucosamine, D-ribose, valerate, L-alanine, L-asparagine, L-glutamine, L-glutamate, L-isoleucine, L-serine and Lthreonine.	TMAO, nitrate	nd	nd
82	Shewanella sp. MR-4	nd	nd	Mesophile	nd	High	nd	Facultative anaerobe	I	lactate, maltose	nd	nd	nd
83	Shewanella sp. TC10	nd	nd	Mesophile	nd	High	nd	Facultative anaerobe	nd	nd	nitrate	nd	nd
84	Shewanella baltica OS678	nd	nd	Mesophile	nd	High	Heterotroph	Facultative anaerobe	nd	nd	nd	nd	nd
85	Shewanella denitrificans OS217	nd	20 - 25	Mesophile	nd	High	Chemoheterotroph	Facultative anaerobe	I	glucose, N-acetylglucosamine and maltose	nitrate, nitrite, sulphite	nd	nd
86	Shewanella dokdonensis DSM 23626	7	25 - 30	Mesophile	1	High	nd	Facultative anaerobe	I	lactate	nitrate, DMSO, TMAO, sodium thiosulfate, sodium nitrate	nd	nd
87	Shewanella frigidimarina NCIMB 400	nd	nd	Mesophile	nd	High	nd	Facultative anaerobe	I	lactate	nitrate, nitrite, trimethylamine N-oxide, Fe(III) and	Y	nd
88	Shewanella japonica KCTC 22435 (=ATCC: BAA-316)	7.5	20 - 25	Mesophile	nd	Medium	Heterotroph	Facultative anaerobe	C	galactose, fructose, maltose, N-acetylglucosamine, succinate, xylose, glucose, acetate, D-glucosamine, pyruvate and cellobiose	nitrate	nd	nd
89	Shewanella litoralis JCM 32306	7	25	Mesophile	2.5	High	nd	Aerobe	nd	nd	nitrate	nd	nd
90	Shewanella livingstonensis LMG 19866	7 - 8	15	Psychrophile	nd	High	Chemoorganotroph	Facultative anaerobe	I	D-glucose, maltose, cellobiose, sucrose, D-mannose, D-mannitol, D-galactose, D-xylose, N-	nitrate, TMAO, ferric compounds	Y	nd
91	Shewanella marina JCM 15074	7	30	Mesophile	3	High	nd	Facultative anaerobe	I	DL-lactic acid, L-arabinose, D-glucose, L-rhamnose, citric acid, malonic acid, propionic acid, L-alanine, Lalanyl glycine and a-hydroxybutyric acid.	nd	nd	nd
92	Shewanella maritima D4-2	7	29 - 32	Mesophile	2	High	nd	Facultative anaerobe	nd	nd	nitrate	nd	nd
93	Shewanella saliphila JCM 32304	7	25	Mesophile	4.5	High	nd	Facultative anaerobe	nd	nd	nitrate	nd	nd
94	Shewanella ulleungensis JCM 32305	7	25	Mesophile	2.5	High	nd	Aerobe	I	maltose	nitrate	nd	nd
95	Shewanella woodyi ATCC 51908	nd	25	Mesophile	3	High	Prototroph	Facultative anaerobe	C	D-galactose, cellobiose, D-glucuronic acid, acetate, a-ketoglutarate, propionate, succinate, L-alanine, L-threonine, L-leucine, L-serine, and putrescine.	O2, nitrate, nitrite, fumarate,	nd	nd
96	Shewanella aestuarii JCM 17801	7	25 - 30	Mesophile	0 - 2	High	nd	Facultative anaerobe	I	lactate, N-Acetyl-D-glucosamine, D-glucose, DL-lactic acid, DL-malic acid, maltose and succinic acid	nitrate, trimethylamine N-oxide	N	nd
97	Shewanella algidipiscicola H1	nd	nd	Mesophile	1.5	High	nd	Aerobe	nd	nd	nd	nd	nd
98	Shewanella amazonensis SB2B	7 - 8	37	Mesophile	1	Medium	nd	Facultative anaerobe	C	acetate, succinate, fumarate and citrate	iron and manganese oxides, thiosulfate, elemental sulfur, TMAO, nitrate	Y	nd
99	Shewanella atlantica HAW-EB5	nd	10	Psychrophile	1.5 - 3	High	nd	Aerobe	C	Acetate, malate, valerate, fructose, peptone and yeast extract	nitrate, TMAO, MnO2, nitrite, thiosulfate, RDX	N	nd

100	Shewanella canadensis HAW-EB2	nd	10	Psychrophile	1.5 - 3	Medium	nd	Aerobe	C	N-Acetyl-d-glucosamine, acetate, b-hydroxybutyrate, DL-lactate, propionate, succinate, malate, succinate, valerate, peptone and yeast extract	TMAO, MnO2, nitrate, nitrite, RDX	N	nd
101	Shewanella chilikensis JC5	8	28 - 30	Mesophile	nd	High	Organoheterotroph	Facultative anaerobe	I	fumarate, malate, pyruvate, succinate, glycogen, N-acetylgalactosamine, N-acetylglucosamine	nd	nd	nd
102	Shewanella donghaensis LT17	7 - 7.5	17	Psychrophile	2.5	High	Heterotroph	Facultative anaerobe	nd	nd	nitrate	nd	nd
103	Shewanella fidelis ATCC BAA-318	7.5	20 - 25	Mesophile	nd	High	Heterotroph	Facultative anaerobe	I	D-Glucose	nitrate	N	nd
104	Shewanella indica KJW27	7.5	37	Mesophile	2	High	nd	Facultative anaerobe	I	lactate, Tween 40, Tween 80, N-acetyl-D-galactosamine, N-acetyl-D-glucosamine, α-D-glucose, pyruvic acid methyl ester, succinic acid monomethyl ester, acetic acid, cis-aconitic acid, citric acid, formic acid, α-hydroxybutyric acid, β-hydroxybutyric acid, α-ketoglutaric acid, D,L-lactic acid, propionic acid, succinic acid, bromosuccinic	nitrate, sulfur, TMAO, DMSO, nitrite, thiosulfate	N	nd
105	Shewanella inventionis CGMCC 1.15339	7	28	Mesophile	2	High	nd	Aerobe	I	Mannitol and malate	nitrate	nd	nd
106	Shewanella litorisediminis SMK1-12	7 - 8	30 - 37	Mesophile	2	High	nd	Facultative anaerobe	C	D-Glucose, D-cellobiose, maltose, acetate, L-malate, pyruvate, succinate and salicin	nitrate	nd	nd
107	Shewanella mangrovi YQH10	6	28	Mesophile	3	High	nd	Facultative anaerobe	I	D-glucose, L-arabinose and malic acid	nitrate	nd	nd
108	Shewanella marisflavi EP1	nd	nd	Mesophile	nd	High	nd	Facultative anaerobe	I	L-lactate	Fe(III) oxide, fumarate, ferric citrate, nitrate, 2,4-DNT	Y	nd
109	Shewanella oneidensis MR-1	7 - 8	25 - 35	Mesophile	0 - 1.7	Medium	nd	Facultative anaerobe	I	Lactate, succinate and fumarate	O2, oxidized metals (including Mn(III) and (IV), Fe(III), Cr(VI), U(VI)), fumarate, nitrate, trimethylamine N-oxide, dimethyl sulfoxide, sulfite. thiosulfate. and elemental sulfur	Y	nd
110	Shewanella piezotolerans WP3	7	15 - 20	Psychrotolerant	3 - 4	High	nd	Facultative anaerobe	I	a-cyclodextrin, dextrin, Tweens 40 and 80, N-acetyl-D-glucosamine, gentiobiose, a-D-glucose, maltose, acetic acid, DL-lactic acid, propionic acid, L-alanine, L-alanyl glycine, L-asparagine, L-glutamic acid, glycyl L-aspartic acid, glycyl L-glutamic acid, L-leucine, L-serine, L-threonine, inosine, uridine and thymidine	nitrate, fumarate, TMAO, DMSO and insoluble Fe(III)	Y	2
111	Shewanella psychrophila WP2	7	10 - 15	Psychrophile	3 - 4	High	nd	Facultative anaerobe	I	Tweens 40 and 80, N-acetyl-Dglucosamine, myo-inositol, maltose, sucrose, D-trehalose, cis-aconitic acid, a-ketovaleric acid, bromosuccinic acid, succinamic acid, L-alaninamide, D-alanine, L-glutamic acid, glycyl L-glutamic acid, L-histidine, hydroxy-L-proline, Lleucine, D-serine L-serine, L-threonine, DL-carnitine, thymidine and DL-a-glycerol phosphate.	nitrate, TMAO, DMSO	N	2.5
112	Shewanella sediminis HAW-EB3	nd	10	Psychrophile	2	High	nd	Facultative anaerobe	C	N-Acetyl-D-glucosamine,<br>Tween 40, Tween 80, acetate, succinate, butyrate, valerate,<br>pyruvate, serine, proline, peptone and yeast extract. Tween 20, malate, propionate and glutamic acid are weak carbon sources.	TMAO, MnO2, nitrate, nitrite,<br>thiosulfate and RDX	N	nd
113	Shewanella sp. Arc9-LZ	nd	15	Psychrotolerant	nd	High	nd	Facultative anaerobe	nd	nd	nd	nd	nd
114	Shewanella sp. M16	nd	20 - 30	Mesophile	nd	nd	nd	Facultative anaerobe	nd	nd	nd	nd	nd
115	Shewanella sp. W3-18-1	nd	nd	Mesophile	nd	High	nd	Facultative anaerobe	I	lactate	Ferric citrate, α-FeO(OH), β-FeO(OH), and Fe2O3, nitrate, nitrite	Y	nd
116	Shewanella vesiculosa LMG 24424	7.5	15 - 20	Psychrotolerant	2	High	Chemoorganotroph	Facultative anaerobe	I	lactate	sodium nitrate, trimethylamine N-oxide, nitrate, thiosulfate, H2	nd	nd
117	Shewanella violacea DSS12	nd	8	Psychrophile	3	High	Chemoorganotroph	Facultative anaerobe	I	glucose	nitrate	nd	nd
118	Shewanella hanedai JCM 20706	nd	nd	Psychrophile	nd	High	nd	Facultative anaerobe	nd	nd	nitrate	nd	nd

119	Shewanella sp. HN-41	nd	nd	Mesophile	nd	Medium	nd	Anaerobe	I	lactate, pyruvate, formate	As(V), S2O3-2, Fe(III)-citrate, poorly crystalline Fe(III)-oxyhydroxide	Y	nd
120	Shewanella halifaxensis HAW-EB4	nd	10	Psychrophile	2	High	nd	Facultative anaerobe	C	glucose, lactate, acetate	MnO2, nitrate, nitrite, TMAO, thiosulfate and RDX	N	nd
121	Shewanella sp. LZH-2	nd	nd	Mesophile	nd	Low	nd	Facultative anaerobe	nd	nd	nd	nd	nd
122	Shewanella sp. BF02_Schw	nd	nd	Psychrophile	nd	Medium	nd	Aerobe	nd	nd	nd	nd	nd
123	Shewanella fodinae KCTC 22506	6	30	Mesophile	4 - 6	High	Chemoorganoheterotroph	Facultative anaerobe	C	acetate, butyrate, lactate, pyruvate, valerate, benzoate, butanol, ethanol, formate, fumerate, sorbitol and succinate	thiosulfate	nd	nd
124	Shewanella sp. ANA-3	nd	nd	Mesophile	nd	Medium	nd	Facultative anaerobe	I	lactate, fumarate, nitrate, and trimethyl-N-amine oxide (TMAO), pyruvate	As(V), As (III) oxygen, soluble ferric iron, oxides of i	Y	5.3
125	Shewanella glacialimarina TZS-4	7	15	Psychrophile	0.5 - 2	High	Psychrotrophic	Facultative anaerobe	I	glucose and maltose	nd	nd	nd
126	Shewanella nanhaiensis NR704-98	7	20 - 25	Mesophile	2	High	nd	Facultative anaerobe	nd	nd	nitrate	nd	nd
