# Supplementary material for: Novel Insights on Extracellular Electron Transfer Networks in the Desulfovibrionaceae Family: Unveiling the Potential Significance of Horizontal Gene Transfer
Source: Microorganisms. 2024 Aug 29;12(9):1796. doi: 10.3390/microorganisms12091796 (PMC11434368; doi:10.3390/microorganisms12091796)
Supplement: Supplementary file 1 [file microorganisms-12-01796-s001.zip › Tables.zip!/TableS4_mobilome.pdf]

		Prophages						Integron_Finder Results										CRISPRFinder Results			ISFinder			ersity of ICEs_by_Macsyfir	
No	Strain	Intact phage	Incomplete phages2	Questionable phage	Intact+incomplete	Total (intact+incomplete+q)	Has_prophage	Total Integrons	Complete Integrons	Complete and In0 Integrons	Total integrons per genome	Complete integrons per genome	Complete and In0 per genome	Total integrons per Mbp	Complete integrons per Mbp	Complete and In0 per Mbp	Has_Integrons	Sum_Evidence_2_4	CAS	Has_CRISPR	Number of Insertion Sequences	Average Length Sequence	Has_IS	Type of ICEs	Has_ICEs
32	Desulfocurvibacter africanus PCS	0	0	1	0	1	1	0	0	0	0	0	0	0	0	0	0	0	3	0	0	-	0	0	0
2	Desulfocurvibacter africanus subsp. africanus DSM 2603	2	3	1	5	6	1	0	0	0	0	0	0	0	0	0	0	0	2	0	0	-	0	0	0
4	Desulfocurvibacter africanus subsp. africanus str. Walvis Bay	2	0	1	2	3	1	0	0	0	0	0	0	0	0	0	0	0	0	0	0	-	0	0	0
12	Desulfocurvus vexinensis DSM 17965	1	1	0	2	2	1	0	0	0	0	0	0	0	0	0	0	2	5	1	0	-	0	0	0
13	Desulfohalovibrio alkalitolerans DSM 16529	1	0	1	1	2	1	0	0	0	0	0	0	0	0	0	0	4	5	1	0	-	0	0	0
20	Desulfovibrio aminophilus DSM 12254	0	2	0	2	2	1	1	0	0	1	0	0	0	0	0	0	0	2	0	0	-	0	2	1
23	Desulfovibrio carbinoliphilus subsp. oakridgensis FW-101-2B	0	3	0	3	3	1	0	0	0	0	0	0	0	0	0	0	1	0	1	0	-	0	1	1
11	Desulfovibrio cuneatus DSM 11391	0	4	0	4	4	1	0	0	0	0	0	0	0	0	0	0	0	4	0	0	-	0	3	1
43	Desulfovibrio desulfuricans AY5	0	1	1	1	2	1	0	0	0	0	0	0	0	0	0	0	1	3	1	0	-	0	2	1
39	Desulfovibrio desulfuricans DFI.2.30	1	5	0	6	6	1	0	0	0	0	0	0	0	0	0	0	1	5	1	2	1033	1	1	1
38	Desulfovibrio desulfuricans DFI.2.72	1	5	0	6	6	1	0	0	0	0	0	0	0	0	0	0	1	5	1	2	1033	1	1	1
37	Desulfovibrio desulfuricans DFI.2.73	1	5	0	6	6	1	0	0	0	0	0	0	0	0	0	0	1	5	1	2	1033	1	1	1
26	Desulfovibrio desulfuricans DSM 642	0	2	0	2	2	1	0	0	0	0	0	0	0	0	0	0	0	3	0	0	-	0	1	1
42	Desulfovibrio desulfuricans DSM 7057 (strain G11)	1	3	0	4	4	1	0	0	0	0	0	0	0	0	0	0	1	11	1	0	-	0	4	1
36	Desulfovibrio desulfuricans Edelweiss	0	2	0	2	2	1	0	0	0	0	0	0	0	0	0	0	1	1	1	0	-	0	1	1
41	Desulfovibrio desulfuricans IC1	0	1	0	1	1	1	0	0	0	0	0	0	0	0	0	0	1	1	1	0	-	0	0	0
35	Desulfovibrio desulfuricans L4	0	1	0	1	1	1	0	0	0	0	0	0	0	0	0	0	1	1	1	1	1778	1	4	1
34	Desulfovibrio desulfuricans MCC432	1	0	0	1	1	1	0	0	0	0	0	0	0	0	0	0	3	2	1	1	708	1	1	1
40	Desulfovibrio desulfuricans NBRC 13699 (ATCC 7757=NCIB 8372)	0	2	0	2	2	1	0	0	0	0	0	0	0	0	0	0	0	2	0	1	1357	1	0	0
29	Desulfovibrio inopinatus DSM 10711	0	3	0	3	3	1	0	0	0	0	0	0	0	0	0	0	2	3	1	0	-	0	0	0
10	Desulfovibrio oxyclinae DSM 11498	0	3	0	3	3	1	0	0	0	0	0	0	0	0	0	0	2	1	1	0	-	0	0	0
9	Desulfovibrio piger ATCC 29098	2	1	1	3	4	1	0	0	0	0	0	0	0	0	0	0	2	1	1	0	-	0	0	0
19	Desulfovibrio sp. A2	1	2	1	3	4	1	0	0	0	0	0	0	0	0	0	0	1	5	1	1	1357	1	0	0
18	Desulfovibrio sp. X2	0	0	1	0	1	1	0	0	0	0	0	0	0	0	0	0	1	7	1	1	1482	1	0	0

33	Desulfovibrio vulgaris DP4	0	4	0	4	4	1	0	0	0	0	0	0	0	0	0	0	1	1	1	3	1213	1	0	0
1	Desulfovibrio vulgaris str. Hildenborough (DSM 644, ATCC 29579)	3	1	1	4	5	1	0	0	0	0	0	0	0	0	0	0	1	1	1	10	1265	1	0	0
28	Fundidesulfovibrio putealis DSM 16056	1	1	0	2	2	1	0	0	0	0	0	0	0	0	0	0	0	1	0	1	1239	1	6	1
44	Geobacter anodireducens PheS2	0	2	0	2	2	1	1	1	1	1	1	1	0	0	0	1	1	5	1	12	1276	1	0	0
45	Geobacter argillaceus ATCC BAA-1139	0	2	0	2	2	1	1	0	1	1	0	1	0	0	0	0	1	5	1	1	899	1	1	1
59	Geobacter benzoatilyticus Jerry-YX	0	1	1	1	2	1	1	1	1	1	1	1	0	0	0	1	1	1	1	4	1976	1	0	0
46	Geobacter chapellei DSM 13688	0	1	0	1	1	1	0	0	0	0	0	0	0	0	0	0	1	7	1	3	973	1	1	1
47	Geobacter daltonii FRC-32	0	8	0	8	8	1	1	0	1	1	0	1	0	0	0	0	1	1	1	0	-	0	0	0
48	Geobacter grbiciae DSM 13689	0	3	0	3	3	1	1	1	1	1	1	1	0	0	0	1	1	3	1	7	1253	1	3	1
49	Geobacter hydrogenophilus DSM 13691	0	1	0	1	1	1	2	1	2	2	1	2	0	0	0	1	0	0	0	2	806	1	0	0
50	Geobacter luticola JCM 17780	0	7	0	7	7	1	1	0	1	1	0	1	0	0	0	0	1	3	1	1	813	1	0	0
51	Geobacter metallireducens GS-15	0	8	0	8	8	1	2	1	2	2	1	2	0	0	0	1	1	1	1	72	1369	1	1	1
52	Geobacter metallireducens RCH3	0	6	0	6	6	1	2	2	2	2	2	2	1	1	1	1	1	10	1	12	1205	1	1	1
53	Geobacter pickeringii G13	0	3	0	3	3	1	0	0	0	0	0	0	0	0	0	0	2	2	1	10	1098	1	0	0
54	Geobacter soli GSS01	0	1	0	1	1	1	2	0	1	2	0	1	1	0	0	0	1	1	1	4	1926	1	0	0
55	Geobacter sp. AOG1	0	3	0	3	3	1	0	0	0	0	0	0	0	0	0	0	1	1	1	0	-	0	1	1
56	Geobacter sp. AOG2	0	3	0	3	3	1	0	0	0	0	0	0	0	0	0	0	0	0	0	0	-	0	1	1
57	Geobacter sp. DSM 9736	0	2	0	2	2	1	0	0	0	0	0	0	0	0	0	0	1	1	1	0	-	0	0	0
58	Geobacter sp. FeAm09	0	5	0	5	5	1	0	0	0	0	0	0	0	0	0	0	1	1	1	0	-	0	2	1
60	Geobacter sp. OR-1	0	3	0	3	3	1	0	0	0	0	0	0	0	0	0	0	2	4	1	1	952	1	0	0
61	Geobacter sp. SVR	0	3	0	3	3	1	0	0	0	0	0	0	0	0	0	0	1	1	1	7	1015	1	1	1
62	Geobacter sulfurreducens KN400	0	0	1	0	1	1	2	0	2	2	0	2	1	0	1	0	1	1	1	8	1350	1	0	0
63	Geobacter sulfurreducens PCA	0	0	1	0	1	1	1	1	1	1	1	1	0	0	0	1	2	1	1	25	1371	1	0	0
64	Geobacter sulfurreducens YM18	0	2	0	2	2	1	0	0	0	0	0	0	0	0	0	0	0	0	0	3	885	1	2	1
65	Geobacter sulfurreducens YM35	0	1	1	1	2	1	1	0	1	1	0	1	0	0	0	0	1	1	1	4	1507	1	1	1
66	Geobacter uraniireducens Rf4	0	2	0	2	2	1	1	0	1	1	0	1	0	0	0	0	1	1	1	58	1508	1	0	0
3	Halodesulfovibrio aestuarii DSM 17919	1	3	0	4	4	1	0	0	0	0	0	0	0	0	0	0	0	0	0	0	-	0	2	1
24	Maridesulfovibrio bastinii DSM 16055	0	3	0	3	3	1	0	0	0	0	0	0	0	0	0	0	5	6	1	0	-	0	0	0
16	Maridesulfovibrio frigidus DSM 17176	1	0	0	1	1	1	0	0	0	0	0	0	0	0	0	0	0	0	0	0	-	0	6	1
17	Maridesulfovibrio hydrothermalis DSM 14728	1	0	1	1	2	1	0	0	0	0	0	0	0	0	0	0	2	2	1	0	-	0	2	1

15	Maridesulfovibrio salexigens DSM 2638	0	1	0	1	1	1	0	0	0	0	0	0	0	0	0	0	0	0	0	0	-	0	0	0
27	Maridesulfovibrio zosterae DSM 11974	0	2	0	2	2	1	0	0	0	0	0	0	0	0	0	0	1	0	1	0	-	0	1	1
8	Megalodesulfovibrio gigas DSM 1382	0	3	0	3	3	1	0	0	0	0	0	0	0	0	0	0	1	1	1	0	-	0	1	1
22	Oleidesulfovibrio alaskensis DSM 16109	1	1	1	2	3	1	0	0	0	0	0	0	0	0	0	0	0	0	0	1	1195	1	0	0
30	Paucidesulfovibrio longus DSM 6739	0	0	1	0	1	1	0	0	0	0	0	0	0	0	0	0	2	2	1	0	-	0	0	0
5	Pseudodesulfovibrio aespoeensis Aspo-2	2	0	1	2	3	1	0	0	0	0	0	0	0	0	0	0	0	0	0	3	722	1	1	1
31	Pseudodesulfovibrio mercurii ND132	0	2	0	2	2	1	0	0	0	0	0	0	0	0	0	0	1	1	1	0	-	0	0	0
21	Pseudodesulfovibrio piezophilus C1TLV30	0	1	0	1	1	1	0	0	0	0	0	0	0	0	0	0	0	0	0	0	-	0	0	0
96	Shewanella aestuarii JCM 17801	0	3	0	3	3	1	3	1	1	3	1	1	1	0	0	1	0	0	0	1	1084	1	0	0
68	Shewanella algae ATCC 49138	1	2	0	3	3	1	3	2	2	3	2	2	1	0	0	1	1	1	1	31	1248	1	0	0
69	Shewanella algicola JCM 31091	1	2	1	3	4	1	5	1	2	5	1	2	1	0	0	1	1	15	1	20	1272	1	1	1
97	Shewanella algidipiscicola H1	0	2	0	2	2	1	3	1	1	3	1	1	1	0	0	1	1	2	1	2	792	1	0	0
98	Shewanella amazonensis SB2B	0	1	0	1	1	1	2	2	2	2	2	2	0	0	0	1	0	0	0	1	1230	1	1	1
99	Shewanella atlantica HAW-EB5	0	1	0	1	1	1	1	1	1	1	1	1	0	0	0	1	0	2	0	5	1145	1	1	1
84	Shewanella baltica OS678	3	9	1	12	13	1	4	0	1	4	0	1	1	0	0	0	1	1	1	24	1427	1	1	1
100	Shewanella canadensis HAW-EB2	1	1	0	2	2	1	1	1	1	1	1	1	0	0	0	1	1	1	1	6	1251	1	0	0
70	Shewanella carassii TUM17387	1	0	0	1	1	1	2	1	1	2	1	1	0	0	0	1	1	1	1	1	1086	1	0	0
101	Shewanella chilikensis JC5	1	0	0	1	1	1	5	1	1	5	1	1	1	0	0	1	1	11	1	7	1159	1	0	0
85	Shewanella denitrificans OS217	1	1	1	2	3	1	1	1	1	1	1	1	0	0	0	1	0	0	0	39	1191	1	0	0
86	Shewanella dokdonensis DSM 23626	3	1	0	4	4	1	0	0	0	0	0	0	0	0	0	0	0	0	0	3	1047	1	0	0
102	Shewanella donghaensis LT17	0	1	0	1	1	1	2	2	2	2	2	2	0	0	0	1	0	0	0	3	1234	1	0	0
103	Shewanella fidelis ATCC BAA-318	0	4	0	4	4	1	1	1	1	1	1	1	0	0	0	1	0	1	0	0	-	0	1	1
123	Shewanella fodinae KCTC 22506	1	2	0	3	3	1	2	1	1	2	1	1	1	0	0	1	0	1	0	5	1050	1	0	0
87	Shewanella frigidimarina NCIMB 400	0	1	1	1	2	1	0	0	0	0	0	0	0	0	0	0	0	1	0	27	1715	1	1	1

71	Shewanella gelidii JCM 30804	0	2	0	2	2	1	10	1	1	10	1	1	3	0	0	1	0	3	0	2	772	1	0	0
125	Shewanella glacialimarina TZS-4	2	1	0	3	3	1	1	1	1	1	1	1	0	0	0	1	0	0	0	2	1314	1	0	0
72	Shewanella glacialipiscicola T147	0	1	1	1	2	1	6	0	0	6	0	0	1	0	0	0	0	18	0	25	1181	1	1	1
73	Shewanella hafniensis ATCC BAA-1207	2	1	0	3	3	1	1	0	0	1	0	0	0	0	0	0	2	14	1	18	1323	1	1	1
120	Shewanella halifaxensis HAW-EB4	0	2	0	2	2	1	1	1	1	1	1	1	0	0	0	1	0	0	0	2	1465	1	0	0
118	Shewanella hanedai JCM 20706	0	2	0	2	2	1	3	1	1	3	1	1	1	0	0	1	0	6	0	3	1244	1	0	0
104	Shewanella indica KJW27	0	1	0	1	1	1	2	0	0	2	0	0	0	0	0	0	1	5	1	4	1084	1	1	1
78	Shewanella intestini MCCC 1A01895	0	1	0	1	1	1	4	1	1	4	1	1	1	0	0	1	0	1	0	3	975	1	1	1
105	Shewanella inventionis CGMCC 1.15339	0	1	0	1	1	1	4	1	1	4	1	1	1	0	0	1	1	5	1	18	1356	1	0	0
88	Shewanella japonica KCTC 22435 (=ATCC: BAA-316)	0	2	0	2	2	1	2	1	1	2	1	1	0	0	0	1	0	0	0	2	1188	1	1	1
74	Shewanella khirikhana TH2012	1	0	0	1	1	1	3	2	2	3	2	2	1	0	0	1	0	0	0	2	1155	1	1	1
89	Shewanella litoralis JCM 32306	0	3	0	3	3	1	1	0	0	1	0	0	0	0	0	0	0	7	0	9	1073	1	0	0
106	Shewanella litorisediminis SMK1-12	0	1	0	1	1	1	2	2	2	2	2	2	0	0	0	1	0	0	0	4	1013	1	1	1
90	Shewanella livingstonensis LMG 19866	0	0	2	0	2	1	1	1	1	1	1	1	0	0	0	1	0	0	0	19	1217	1	0	0
107	Shewanella mangrovi YQH10	0	1	0	1	1	1	3	1	1	3	1	1	1	0	0	1	0	3	0	1	1448	1	0	0
91	Shewanella marina JCM 15074	0	1	0	1	1	1	2	1	1	2	1	1	0	0	0	1	0	3	0	0	-	0	0	0
108	Shewanella marisflavi EP1	0	0	0	0	0	0	1	0	0	1	0	0	0	0	0	0	0	0	0	0	-	0	1	1
92	Shewanella maritima D4-2	0	2	0	2	2	1	2	1	1	2	1	1	0	0	0	1	0	0	0	0	-	0	0	0
75	Shewanella morhuae ATCC BAA-1205	1	0	0	1	1	1	0	0	0	0	0	0	0	0	0	0	0	3	0	9	1112	1	0	0
126	Shewanella nanhaiensis NR704-98	0	2	0	2	2	1	0	0	0	0	0	0	0	0	0	0	0	3	0	2	1222	1	0	0
109	Shewanella oneidensis MR-1	7	6	2	13	15	1	2	1	1	2	1	1	0	0	0	1	0	0	0	52	1315	1	0	0
76	Shewanella pealeana ATCC 700345	0	1	0	1	1	1	1	0	0	1	0	0	0	0	0	0	0	0	0	10	1377	1	0	0
110	Shewanella piezotolerans WP3	1	0	0	1	1	1	2	1	1	2	1	1	0	0	0	1	0	0	0	20	1322	1	0	0
77	Shewanella polaris SM1901	0	1	1	1	2	1	1	0	1	1	0	1	0	0	0	0	0	0	0	4	1198	1	1	1
111	Shewanella psychrophila WP2	1	4	1	5	6	1	2	0	2	2	0	2	0	0	0	0	0	0	0	22	1254	1	0	0
67	Shewanella putrefaciens ATCC 8071	0	2	0	2	2	1	3	0	1	3	0	1	1	0	0	0	2	1	1	0	-	0	0	0
80	Shewanella sairae JCM 11563	0	1	0	1	1	1	4	1	1	4	1	1	1	0	0	1	0	15	0	13	1046	1	7	1
93	Shewanella saliphila JCM 32304	0	2	0	2	2	1	2	1	1	2	1	1	0	0	0	1	0	7	0	8	988	1	0	0
81	Shewanella schlegeliana JCM 11561	0	1	0	1	1	1	1	0	1	1	0	1	0	0	0	0	0	2	0	3	1146	1	1	1

112	Shewanella sediminis HAW-EB3	0	1	0	1	1	1	0	0	0	0	0	0	0	0	0	0	0	0	0	5	1214	1	0	0
124	Shewanella sp. ANA-3	0	1	0	1	1	1	4	0	0	4	0	0	1	0	0	0	1	0	1	17	1891	1	2	1
113	Shewanella sp. Arc9-LZ	0	3	0	3	3	1	0	0	0	0	0	0	0	0	0	0	0	0	0	11	1223	1	0	0
122	Shewanella sp. BF02_Schw	1	5	0	6	6	1	1	0	0	1	0	0	0	0	0	0	3	1	1	16	1196	1	0	0
119	Shewanella sp. HN-41	0	3	0	3	3	1	9	1	1	9	1	1	2	0	0	1	0	4	0	2	1219	1	0	0
121	Shewanella sp. LZH-2	1	2	0	3	3	1	0	0	0	0	0	0	0	0	0	0	0	0	0	6	1387	1	0	0
114	Shewanella sp. M16	3	1	0	4	4	1	4	1	1	4	1	1	1	0	0	1	0	5	0	20	945	1	2	1
82	Shewanella sp. MR-4	0	1	0	1	1	1	3	0	0	3	0	0	1	0	0	0	0	0	0	7	1338	1	0	0
83	Shewanella sp. TC10	0	4	0	4	4	1	9	0	0	9	0	0	2	0	0	0	0	12	0	6	1123	1	0	0
115	Shewanella sp. W3-18-1	1	4	1	5	6	1	1	0	0	1	0	0	0	0	0	0	2	1	1	36	1794	1	1	1
94	Shewanella ulleungensis JCM 32305	0	1	0	1	1	1	5	1	1	5	1	1	1	0	0	1	0	1	0	2	1657	1	0	0
116	Shewanella vesiculosa LMG 24424	0	1	1	1	2	1	5	1	2	5	1	2	1	0	0	1	0	1	0	8	1367	1	0	0
117	Shewanella violacea DSS12	0	1	0	1	1	1	0	0	0	0	0	0	0	0	0	0	0	0	0	11	1285	1	0	0
95	Shewanella woodyi ATCC 51908	0	0	0	0	0	0	3	2	2	3	2	2	1	0	0	1	0	0	0	5	1417	1	1	1
25	Solidesulfovibrio alcoholivorans DSM 5433	0	1	0	1	1	1	0	0	0	0	0	0	0	0	0	0	1	4	1	4	1566	1	1	1
14	Solidesulfovibrio fructosivorans JJ	1	2	0	3	3	1	0	0	0	0	0	0	0	0	0	0	2	5	1	0	-	0	0	0
7	Solidesulfovibrio magneticus RS-1	0	2	0	2	2	1	0	0	0	0	0	0	0	0	0	0	2	1	1	1	2262	1	1	1
