# Supplementary material for: Novel Insights on Extracellular Electron Transfer Networks in the Desulfovibrionaceae Family: Unveiling the Potential Significance of Horizontal Gene Transfer
Source: Microorganisms. 2024 Aug 29;12(9):1796. doi: 10.3390/microorganisms12091796 (PMC11434368; doi:10.3390/microorganisms12091796)
Supplement: Supplementary file 1 [file microorganisms-12-01796-s001.zip › Tables.zip!/TableS5_prevalence_mobilome.pdf]

No	Strain	Has_Integrons	Has_IS	Has_ICEs	Has_prophage	Has_CRISPR
32	Desulfocurvibacter africanus PCS	0	0	0	1	0
2	Desulfocurvibacter africanus subsp. africanus DSM 2603	0	0	0	1	0
4	Desulfocurvibacter africanus subsp. africanus str. Walvis Bay	0	0	0	1	0
12	Desulfocurvus vexinensis DSM 17965	0	0	0	1	1
13	Desulfohalovibrio alkalitolerans DSM 16529	0	0	0	1	1
20	Desulfovibrio aminophilus DSM 12254	0	0	1	1	0
23	Desulfovibrio carbinoliphilus subsp. oakridgensis FW-101-2B	0	0	1	1	1
11	Desulfovibrio cuneatus DSM 11391	0	0	1	1	0
43	Desulfovibrio desulfuricans AY5	0	0	1	1	1
39	Desulfovibrio desulfuricans DFI.2.30	0	1	1	1	1
38	Desulfovibrio desulfuricans DFI.2.72	0	1	1	1	1
37	Desulfovibrio desulfuricans DFI.2.73	0	1	1	1	1
26	Desulfovibrio desulfuricans DSM 642	0	0	1	1	0
42	Desulfovibrio desulfuricans DSM 7057 (strain G11)	0	0	1	1	1
36	Desulfovibrio desulfuricans Edelweiss	0	0	1	1	1
41	Desulfovibrio desulfuricans IC1	0	0	0	1	1
35	Desulfovibrio desulfuricans L4	0	1	1	1	1
34	Desulfovibrio desulfuricans MCC432	0	1	1	1	1
40	Desulfovibrio desulfuricans NBRC 13699 (ATCC 7757=NCIB 8372)	0	1	0	1	0
29	Desulfovibrio inopinatus DSM 10711	0	0	0	1	1
10	Desulfovibrio oxyclinae DSM 11498	0	0	0	1	1
9	Desulfovibrio piger ATCC 29098	0	0	0	1	1
19	Desulfovibrio sp. A2	0	1	0	1	1
18	Desulfovibrio sp. X2	0	1	0	1	1
33	Desulfovibrio vulgaris DP4	0	1	0	1	1
1	Desulfovibrio vulgaris str. Hildenborough (DSM 644, ATCC 29579)	0	1	0	1	1
28	Fundidesulfovibrio putealis DSM 16056	0	1	1	1	0
44	Geobacter anodireducens PheS2	1	1	0	1	1

45	Geobacter argillaceus ATCC BAA-1139	0	1	1	1	1
59	Geobacter benzoatilyticus Jerry-YX	1	1	0	1	1
46	Geobacter chapellei DSM 13688	0	1	1	1	1
47	Geobacter daltonii FRC-32	0	0	0	1	1
48	Geobacter grbiciae DSM 13689	1	1	1	1	1
49	Geobacter hydrogenophilus DSM 13691	1	1	0	1	0
50	Geobacter luticola JCM 17780	0	1	0	1	1
51	Geobacter metallireducens GS-15	1	1	1	1	1
52	Geobacter metallireducens RCH3	1	1	1	1	1
53	Geobacter pickeringii G13	0	1	0	1	1
54	Geobacter soli GSS01	0	1	0	1	1
55	Geobacter sp. AOG1	0	0	1	1	1
56	Geobacter sp. AOG2	0	0	1	1	0
57	Geobacter sp. DSM 9736	0	0	0	1	1
58	Geobacter sp. FeAm09	0	0	1	1	1
60	Geobacter sp. OR-1	0	1	0	1	1
61	Geobacter sp. SVR	0	1	1	1	1
62	Geobacter sulfurreducens KN400	0	1	0	1	1
63	Geobacter sulfurreducens PCA	1	1	0	1	1
64	Geobacter sulfurreducens YM18	0	1	1	1	0
65	Geobacter sulfurreducens YM35	0	1	1	1	1
66	Geobacter uraniireducens Rf4	0	1	0	1	1
3	Halodesulfovibrio aestuarii DSM 17919	0	0	1	1	0
24	Maridesulfovibrio bastinii DSM 16055	0	0	0	1	1
16	Maridesulfovibrio frigidus DSM 17176	0	0	1	1	0
17	Maridesulfovibrio hydrothermalis DSM 14728	0	0	1	1	1
15	Maridesulfovibrio salexigens DSM 2638	0	0	0	1	0
27	Maridesulfovibrio zosterae DSM 11974	0	0	1	1	1
8	Megalodesulfovibrio gigas DSM 1382	0	0	1	1	1
22	Oleidesulfovibrio alaskensis DSM 16109	0	1	0	1	0
30	Paucidesulfovibrio longus DSM 6739	0	0	0	1	1
5	Pseudodesulfovibrio aespoeensis Aspo-2	0	1	1	1	0
31	Pseudodesulfovibrio mercurii ND132	0	0	0	1	1
21	Pseudodesulfovibrio piezophilus C1TLV30	0	0	0	1	0
96	Shewanella aestuarii JCM 17801	1	1	0	1	0

68	Shewanella algae ATCC 49138	1	1	0	1	1
69	Shewanella algicola JCM 31091	1	1	1	1	1
97	Shewanella algidipiscicola H1	1	1	0	1	1
98	Shewanella amazonensis SB2B	1	1	1	1	0
99	Shewanella atlantica HAW-EB5	1	1	1	1	0
84	Shewanella baltica OS678	0	1	1	1	1
100	Shewanella canadensis HAW-EB2	1	1	0	1	1
70	Shewanella carassii TUM17387	1	1	0	1	1
101	Shewanella chilikensis JC5	1	1	0	1	1
85	Shewanella denitrificans OS217	1	1	0	1	0
86	Shewanella dokdonensis DSM 23626	0	1	0	1	0
102	Shewanella donghaensis LT17	1	1	0	1	0
103	Shewanella fidelis ATCC BAA-318	1	0	1	1	0
123	Shewanella fodinae KCTC 22506	1	1	0	1	0
87	Shewanella frigidimarina NCIMB 400	0	1	1	1	0
71	Shewanella gelidii JCM 30804	1	1	0	1	0
125	Shewanella glacialimarina TZS-4	1	1	0	1	0
72	Shewanella glacialipiscicola T147	0	1	1	1	0
73	Shewanella hafniensis ATCC BAA-1207	0	1	1	1	1
120	Shewanella halifaxensis HAW-EB4	1	1	0	1	0
118	Shewanella hanedai JCM 20706	1	1	0	1	0
104	Shewanella indica KJW27	0	1	1	1	1
78	Shewanella intestini MCCC 1A01895	1	1	1	1	0
105	Shewanella inventionis CGMCC 1.15339	1	1	0	1	1
88	Shewanella japonica KCTC 22435 (=ATCC: BAA-316)	1	1	1	1	0
74	Shewanella khirikhana TH2012	1	1	1	1	0
89	Shewanella litoralis JCM 32306	0	1	0	1	0
106	Shewanella litorisediminis SMK1-12	1	1	1	1	0
90	Shewanella livingstonensis LMG 19866	1	1	0	1	0
107	Shewanella mangrovi YQH10	1	1	0	1	0
91	Shewanella marina JCM 15074	1	0	0	1	0
108	Shewanella marisflavi EP1	0	0	1	0	0
92	Shewanella maritima D4-2	1	0	0	1	0
75	Shewanella morhuae ATCC BAA-1205	0	1	0	1	0

126	Shewanella nanhaiensis NR704-98	0	1	0	1	0
109	Shewanella oneidensis MR-1	1	1	0	1	0
76	Shewanella pealeana ATCC 700345	0	1	0	1	0
110	Shewanella piezotolerans WP3	1	1	0	1	0
77	Shewanella polaris SM1901	0	1	1	1	0
111	Shewanella psychrophila WP2	0	1	0	1	0
67	Shewanella putrefaciens ATCC 8071	0	0	0	1	1
80	Shewanella sairae JCM 11563	1	1	1	1	0
93	Shewanella saliphila JCM 32304	1	1	0	1	0
81	Shewanella schlegeliana JCM 11561	0	1	1	1	0
112	Shewanella sediminis HAW-EB3	0	1	0	1	0
124	Shewanella sp. ANA-3	0	1	1	1	1
113	Shewanella sp. Arc9-LZ	0	1	0	1	0
122	Shewanella sp. BF02_Schw	0	1	0	1	1
119	Shewanella sp. HN-41	1	1	0	1	0
121	Shewanella sp. LZH-2	0	1	0	1	0
114	Shewanella sp. M16	1	1	1	1	0
82	Shewanella sp. MR-4	0	1	0	1	0
83	Shewanella sp. TC10	0	1	0	1	0
115	Shewanella sp. W3-18-1	0	1	1	1	1
94	Shewanella ulleungensis JCM 32305	1	1	0	1	0
116	Shewanella vesiculosa LMG 24424	1	1	0	1	0
117	Shewanella violacea DSS12	0	1	0	1	0
95	Shewanella woodyi ATCC 51908	1	1	1	0	0
25	Solidesulfovibrio alcoholivorans DSM 5433	0	1	1	1	1
14	Solidesulfovibrio fructosivorans JJ	0	0	0	1	1
7	Solidesulfovibrio magneticus RS-1	0	1	1	1	1
