# Supplementary material for: Novel Insights on Extracellular Electron Transfer Networks in the Desulfovibrionaceae Family: Unveiling the Potential Significance of Horizontal Gene Transfer
Source: Microorganisms. 2024 Aug 29;12(9):1796. doi: 10.3390/microorganisms12091796 (PMC11434368; doi:10.3390/microorganisms12091796)
Supplement: Supplementary file 1 [file microorganisms-12-01796-s001.zip › Tables.zip!/TableS6_C-cyst_MEET.pdf]

No	Strain	Proteins with motif							Nº of cytochromes per Genome (Mb)				Nº of cytochromes per cell volume (um3)				Nº cytochromes with subcellular localization - Psortb						Nº cytochromes with subcellular localization per genome size (Mbp)						Nº cytochromes with subcellular localization per cell volume (um3)						Number of copies													
		CXXCH_count	CXXXCH_count	Both motifs	Total2	Mono-heme	Bi-heme	Multi-heme (>=3)	Mono-heme/Mbp	Bi-heme/Mbp	Multi-heme/Mbp	Tot cytochromes /Mbp	Mono-heme/um3	Bi-heme/um3	Multi-heme/um3	Tot cytochromes /um3	Extracellular	OuterMembrane	Periplasmic	CytoplasmicMembrane	Cytoplasmic	Unknown	Extracellular2	OuterMembrane3	Periplasmic4	CytoplasmicMembrane5	Cytoplasmic6	Unknown7	Extracellular22	OuterMembrane33	Periplasmic44	CytoplasmicMembrane55	Cytoplasmic66	Unknown77	PpcA	OmpJ	CbcT	CbcA	CbcB	CbcC/OmcQ	Cbc_	CymA	RibB	RibD	RibH	RibA	RibE	Rib_
32	Desulfocurvibacter africanus PCS	56	12	5	63	40	9	14	10	2	4	16	75	17	26	119	0	0	14	7	27	15	0	0	4	2	7	4	0	0	26	13	51	28	4	1	9	0	0	2	11	1	2	1	1	0	0	4
2	Desulfocurvibacter africanus subsp. africanus DSM 2603	59	12	5	66	43	9	14	10	2	3	15	29	6	10	45	0	0	15	10	29	12	0	0	3	2	7	3	0	0	10	7	20	8	4	2	11	0	0	2	13	1	2	1	1	0	0	4
4	Desulfocurvibacter africanus subsp. africanus str. Walvis Bay	57	12	5	64	40	10	14	10	2	3	15	27	7	10	43	0	0	14	8	26	16	0	0	3	2	6	4	0	0	10	5	18	11	4	2	9	0	0	2	11	1	2	1	1	0	0	4
12	Desulfocurvus vexinensis DSM 17965	46	12	6	52	30	9	13	8	2	4	14	38	11	17	66	0	0	11	10	18	13	0	0	3	3	5	4	0	0	14	13	23	17	3	1	9	0	1	1	11	1	2	1	1	0	0	4
13	Desulfohalovibrio alkalitolerans DSM 16529	44	11	3	52	38	4	10	12	1	3	16	69	7	18	95	0	1	8	10	24	9	0	0	2	3	7	3	0	2	15	18	44	16	3	1	9	0	0	0	9	1	1	1	1	0	0	3
20	Desulfovibrio aminophilus DSM 12254	40	13	4	49	30	5	14	9	1	4	14	273	45	127	445	0	0	10	10	19	10	0	0	3	3	6	3	0	0	91	91	173	91	2	2	12	0	1	1	14	1	2	1	1	0	0	4
23	Desulfovibrio carbinoliphilus subsp. oakridgensis FW-101-2B	48	13	3	58	44	4	10	10	1	2	14	nd	nd	nd	nd	0	0	7	15	20	16	0	0	2	4	5	4	nd	nd	nd	nd	nd	nd	3	8	9	0	0	0	9	1	2	1	1	0	0	4
11	Desulfovibrio cuneatus DSM 11391	45	10	2	53	40	3	10	12	1	3	16	95	7	24	126	0	0	14	9	18	12	0	0	4	3	5	4	0	0	33	21	43	28	3	3	11	0	0	0	11	2	1	1	1	0	0	3
43	Desulfovibrio desulfuricans AY5	44	8	0	52	34	6	12	10	2	3	15	55	10	19	84	0	0	9	10	21	12	0	0	3	3	6	3	0	0	15	16	34	19	3	8	9	1	0	0	10	2	2	1	1	0	0	4
39	Desulfovibrio desulfuricans DFI.2.30	44	9	0	53	37	5	11	10	1	3	15	nd	nd	nd	nd	0	0	8	9	23	13	0	0	2	2	6	4	nd	nd	nd	nd	nd	nd	3	9	11	1	0	0	12	1	2	1	1	0	0	4
38	Desulfovibrio desulfuricans DFI.2.72	45	9	0	54	38	5	11	10	1	3	15	nd	nd	nd	nd	0	0	9	11	21	13	0	0	2	3	6	4	nd	nd	nd	nd	nd	nd	3	9	11	1	0	0	12	1	2	1	1	0	0	4
37	Desulfovibrio desulfuricans DFI.2.73	44	10	0	54	38	5	11	10	1	3	15	nd	nd	nd	nd	0	0	8	9	23	14	0	0	2	2	6	4	nd	nd	nd	nd	nd	nd	3	9	11	1	0	0	12	1	2	1	1	0	0	4
26	Desulfovibrio desulfuricans DSM 642	44	7	0	51	35	5	11	10	1	3	15	714	102	224	1041	0	0	8	9	22	12	0	0	2	3	6	4	0	0	163	184	449	245	3	8	8	0	0	0	8	3	2	1	1	0	0	4
42	Desulfovibrio desulfuricans DSM 7057 (strain G11)	37	7	1	43	28	6	9	9	2	3	13	nd	nd	nd	nd	0	0	8	4	25	6	0	0	2	1	8	2	nd	nd	nd	nd	nd	nd	3	6	5	0	0	1	6	2	2	1	1	0	0	4
36	Desulfovibrio desulfuricans Edelweiss	46	10	0	56	39	4	13	11	1	4	16	nd	nd	nd	nd	0	0	10	10	26	10	0	0	3	3	8	3	nd	nd	nd	nd	nd	nd	3	8	9	1	0	1	11	2	2	1	1	0	0	4
41	Desulfovibrio desulfuricans IC1	41	4	0	45	31	4	10	10	1	3	14	47	6	15	68	0	0	7	9	18	11	0	0	2	3	6	3	0	0	11	14	27	17	3	10	6	1	0	0	7	2	2	1	1	0	0	4
35	Desulfovibrio desulfuricans L4	47	6	0	53	36	5	12	10	1	3	15	nd	nd	nd	nd	0	0	9	10	21	13	0	0	3	3	6	4	nd	nd	nd	nd	nd	nd	3	8	8	1	0	0	9	2	2	1	1	0	0	4
34	Desulfovibrio desulfuricans MCC432	44	8	0	52	35	5	12	10	1	3	15	nd	nd	nd	nd	0	0	8	9	22	13	0	0	2	3	6	4	nd	nd	nd	nd	nd	nd	3	9	10	1	0	0	11	2	2	1	1	0	0	4
40	Desulfovibrio desulfuricans NBRC 13699 (ATCC 7757=NCIB 8372)	54	12	2	64	46	4	14	14	1	4	19	nd	nd	nd	nd	0	0	13	14	23	14	0	0	4	4	7	4	nd	nd	nd	nd	nd	nd	4	4	9	0	1	1	11	1	2	1	1	0	0	4
29	Desulfovibrio inopinatus DSM 10711	43	18	2	59	45	2	12	8	0	2	10	5	0	1	6	0	0	8	7	32	12	0	0	1	1	6	2	0	0	1	1	3	1	3	5	12	0	0	1	13	1	2	1	1	0	0	4

10	Desulfovibrio oxyclinae DSM 11498	42	11	4	49	34	2	13	10	1	4	15	69	4	26	100	0	0	6	10	25	8	0	0	2	3	8	2	0	0	12	20	51	16	4	1	8	0	0	2	10	1	2	1	1	0	0	4
9	Desulfovibrio piger ATCC 29098	32	9	1	40	28	5	7	10	2	2	14	7	1	2	10	0	0	6	5	24	5	0	0	2	2	8	2	0	0	2	1	6	1	2	7	5	0	0	1	6	1	2	1	1	0	0	4
19	Desulfovibrio sp. A2	59	13	2	70	50	5	15	12	1	4	17	177	18	53	247	0	1	16	17	22	14	0	0	4	4	5	3	0	4	57	60	78	49	3	3	14	0	1	0	15	3	2	1	1	0	0	4
18	Desulfovibrio sp. X2	45	18	3	60	44	3	13	11	1	3	15	nd	nd	nd	nd	0	1	10	9	28	12	0	0	3	2	7	3	nd	nd	nd	nd	nd	nd	5	1	9	0	0	1	10	1	2	1	1	0	0	4
33	Desulfovibrio vulgaris DP4	53	12	2	63	45	4	14	12	1	4	17	14	1	4	20	0	0	13	13	23	14	0	0	4	4	6	4	0	0	4	4	7	4	4	4	9	0	1	1	11	1	2	1	1	0	0	4
1	Desulfovibrio vulgaris str. Hildenborough (DSM 644, ATCC 29579)	55	12	2	65	47	4	14	12	1	4	17	23	2	7	32	0	0	13	13	23	16	0	0	3	3	6	4	0	0	6	6	11	8	4	4	9	0	1	1	11	1	2	1	1	0	0	4
28	Fundidesulfovibrio putealis DSM 16056	49	13	2	60	44	2	14	9	0	3	13	36	2	11	49	0	2	9	12	21	16	0	0	2	3	4	3	0	2	7	10	17	13	3	6	13	0	0	0	13	1	1	1	1	0	0	3
44	Geobacter anodireducens PheS2	113	13	8	118	53	6	59	14	2	16	32	nd	nd	nd	nd	8	0	29	14	22	45	2	0	8	4	6	12							5	2	4	6	1	3	14	1	1	1	1	0	0	3
45	Geobacter argillaceus ATCC BAA-1139	107	20	12	115	59	5	51	13	1	12	26	139	12	120	271	11	0	17	15	30	42	3	0	4	3	7	10	26	0	40	35	71	99	3	3	5	5	1	4	15	0	1	1	1	0	0	3
59	Geobacter benzoatilyticus Jerry-YX	108	21	13	116	51	9	56	14	3	16	32	nd	nd	nd	nd	10	1	20	14	27	44	3	0	6	4	8	12							5	1	7	5	1	3	16	2	1	1	1	0	0	3
46	Geobacter chapellei DSM 13688	106	24	13	117	61	8	48	16	2	12	30	144	19	113	276	9	0	15	16	26	51	2	0	4	4	7	13	21	0	35	38	61	120	4	2	6	4	1	4	15	0	1	1	1	0	0	3
47	Geobacter daltonii FRC-32	127	29	17	139	66	8	65	15	2	15	32	420	51	414	885	6	1	21	13	35	63	1	0	5	3	8	15	38	6	134	83	223	401	6	1	9	6	1	7	23	0	1	1	1	0	0	3
48	Geobacter grbiciae DSM 13689	117	18	11	124	59	9	56	14	2	13	30	139	21	132	292	7	0	21	15	32	49	2	0	5	4	8	12	17	0	50	35	75	116	5	1	4	4	1	3	12	1	1	1	1	0	0	3
49	Geobacter hydrogenophilus DSM 13691	110	15	10	115	54	12	49	13	3	12	29	127	28	116	271	10	0	19	18	25	43	2	0	5	4	6	11	24	0	45	42	59	101	4	1	8	4	1	3	16	2	1	1	1	0	0	3
50	Geobacter luticola JCM 17780	117	21	13	125	56	15	54	15	4	15	34	636	170	614	1420	13	1	26	16	21	48	4	0	7	4	6	13	148	11	295	182	239	545	5	2	7	6	1	4	18	0	1	1	1	0	0	3
51	Geobacter metallireducens GS-15	125	18	12	131	62	11	58	15	3	14	33	105	19	98	222	10	0	21	19	31	50	2	0	5	5	8	12	17	0	36	32	53	85	5	1	5	4	1	3	13	1	1	1	1	0	0	3
52	Geobacter metallireducens RCH3	116	18	12	122	54	10	58	14	3	15	32	nd	nd	nd	nd	8	0	21	18	27	48	2	0	6	5	7	13	nd	nd	nd	nd	nd	nd	5	1	5	4	1	3	13	1	1	1	1	0	0	3
53	Geobacter pickeringii G13	108	15	10	113	50	6	57	14	2	16	31	118	14	134	267	12	0	18	11	24	48	3	0	5	3	7	13	28	0	42	26	57	113	4	2	7	4	1	3	15	2	1	1	1	0	0	3
54	Geobacter soli GSS01	115	13	8	120	53	8	59	14	2	16	33	200	30	223	453	8	0	29	14	21	48	2	0	8	4	6	13	30	0	109	53	79	181	5	2	4	6	1	3	14	1	1	1	1	0	0	3
55	Geobacter sp. AOG1	105	14	8	111	51	9	51	14	2	14	30	nd	nd	nd	nd	8	0	22	14	21	46	2	0	6	4	6	13	nd	nd	nd	nd	nd	nd	5	2	7	6	1	4	18	0	1	1	1	0	0	3
56	Geobacter sp. AOG2	107	21	10	118	61	7	50	16	2	13	30	nd	nd	nd	nd	7	1	16	20	26	48	2	0	4	5	7	12	nd	nd	nd	nd	nd	nd	6	2	10	8	3	5	26	0	4	1	1	1	0	7
57	Geobacter sp. DSM 9736	128	24	18	134	57	12	65	14	3	16	34	nd	nd	nd	nd	12	0	26	13	28	55	3	0	7	3	7	14	nd	nd	nd	nd	nd	nd	6	1	9	7	1	4	21	0	1	1	1	0	0	3
58	Geobacter sp. FeAm09	135	21	10	146	66	11	69	16	3	17	36	nd	nd	nd	nd	13	0	33	16	26	58	3	0	8	4	6	14	nd	nd	nd	nd	nd	nd	6	4	9	9	3	6	27	0	1	1	1	0	0	3
60	Geobacter sp. OR-1	132	25	20	137	58	7	72	13	2	16	30	131	16	163	310	26	1	20	13	24	53	6	0	4	3	5	12	59	2	45	29	54	120	5	1	9	4	1	8	22	0	1	1	1	0	0	3
61	Geobacter sp. SVR	94	13	6	101	55	11	35	12	2	7	21	nd	nd	nd	nd	6	0	18	15	24	38	1	0	4	3	5	8	nd	nd	nd	nd	nd	nd	4	2	9	4	1	3	17	1	2	1	1	0	0	4
62	Geobacter sulfurreducens KN400	129	19	12	136	57	8	71	15	2	19	37	nd	nd	nd	nd	11	0	32	18	25	50	3	0	9	5	7	13	nd	nd	nd	nd	nd	nd	5	2	6	9	1	3	19	1	1	1	1	0	0	3
63	Geobacter sulfurreducens PCA	130	18	12	136	57	8	71	15	2	19	36	116	16	145	277	12	0	29	18	23	54	3	0	8	5	6	14	24	0	59	37	47	110	5	2	7	9	1	3	20	1	1	1	1	0	0	3
64	Geobacter sulfurreducens YM18	125	16	11	130	56	8	66	15	2	18	35	nd	nd	nd	nd	10	0	29	20	26	45	3	0	8	5	7	12	nd	nd	nd	nd	nd	nd	5	2	6	9	1	3	19	1	1	1	1	0	0	3
65	Geobacter sulfurreducens YM35	126	17	12	131	55	8	68	15	2	18	35	nd	nd	nd	nd	9	0	33	17	24	48	2	0	9	5	6	13	nd	nd	nd	nd	nd	nd	5	2	7	9	1	3	20	1	1	1	1	0	0	3

66	Geobacter uraniireducens Rf4	146	30	19	157	67	5	85	13	1	17	31	176	13	224	413	17	0	29	12	29	70	3	0	6	2	6	14	45	0	76	32	76	184	4	3	11	10	1	9	31	1	1	1	1	0	0	3
3	Halodesulfovibrio aestuarii DSM 17919	44	7	1	50	33	2	15	9	1	4	14	36	2	16	55	0	0	10	9	21	10	0	0	3	3	6	3	0	0	11	10	23	11	5	5	9	0	1	0	10	1	2	1	1	0	0	4
24	Maridesulfovibrio bastinii DSM 16055	46	10	2	54	34	6	14	9	2	4	14	69	12	29	110	0	0	9	11	26	8	0	0	2	3	7	2	0	0	18	22	53	16	4	1	6	0	1	2	9	1	2	1	1	0	0	4
16	Maridesulfovibrio frigidus DSM 17176	42	8	1	49	33	2	14	8	0	3	12	23	1	10	34	1	0	9	6	21	12	0	0	2	1	5	3	1	0	6	4	15	8	4	1	10	0	0	1	11	1	2	1	1	0	0	4
17	Maridesulfovibrio hydrothermalis DSM 14728	35	6	1	40	25	2	13	7	1	4	11	38	3	20	60	0	1	8	7	19	5	0	0	2	2	5	1	0	2	12	11	29	8	4	1	9	0	1	2	12	1	2	1	1	0	0	4
15	Maridesulfovibrio salexigens DSM 2638	47	10	1	56	38	2	16	9	0	4	13	18	1	7	26	0	0	11	9	26	10	0	0	3	2	6	2	0	0	5	4	12	5	5	2	10	0	1	2	13	1	2	1	1	0	0	4
27	Maridesulfovibrio zosterae DSM 11974	39	9	1	47	34	2	11	8	0	3	11	58	3	19	80	0	0	9	8	23	7	0	0	2	2	6	2	0	0	15	14	39	12	4	1	9	0	1	2	12	0	2	1	1	0	0	4
8	Megalodesulfovibrio gigas DSM 1382	49	13	5	57	40	6	11	11	2	3	15	9	1	3	13	0	0	11	9	24	13	0	0	3	2	6	3	0	0	3	2	6	3	4	2	8	0	1	1	10	1	2	1	1	0	0	4
22	Oleidesulfovibrio alaskensis DSM 16109	45	13	2	56	40	5	11	11	1	3	16	24	3	6	33	0	0	8	12	24	12	0	0	2	3	7	3	0	0	5	7	14	7	3	2	11	0	0	1	12	1	2	1	1	0	0	4
30	Paucidesulfovibrio longus DSM 6739	59	12	2	69	49	5	15	13	1	4	19	41	4	13	58	0	0	14	16	26	13	0	0	4	4	7	4	0	0	12	13	22	11	3	2	9	0	1	2	12	1	2	1	1	0	0	4
5	Pseudodesulfovibrio aespoeensis Aspo-2	39	12	2	49	32	3	14	9	1	4	14	78	7	34	119	0	0	7	6	19	17	0	0	2	2	5	5	0	0	17	15	46	41	3	1	10	0	0	1	11	1	2	1	1	0	0	4
31	Pseudodesulfovibrio mercurii ND132	56	6	2	60	37	6	17	10	2	4	16	52	8	24	85	0	0	12	13	26	9	0	0	3	3	7	2	0	0	17	18	37	13	4	1	17	0	0	3	20	1	2	1	1	0	0	4
21	Pseudodesulfovibrio piezophilus C1TLV30	57	15	2	70	52	2	16	14	1	4	19	88	3	27	119	0	1	9	13	31	16	0	0	2	4	9	4	0	2	15	22	53	27	3	1	11	0	0	3	14	2	2	1	1	0	0	4
96	Shewanella aestuarii JCM 17801	52	5	0	57	31	9	17	8	2	4	15	33	10	18	60	1	0	14	9	16	17	0	0	4	2	4	4	1	0	15	10	17	18	0	0	1	1	0	0	2	3	2	1	1	1	1	6
68	Shewanella algae ATCC 49138	69	7	0	76	40	10	26	8	2	5	16	nd	nd	nd	nd	0	0	20	9	20	27	0	0	4	2	4	6	nd	nd	nd	nd	nd	nd	0	0	8	3	0	0	11	3	2	1	1	1	1	6
69	Shewanella algicola JCM 31091	66	14	2	78	43	12	23	9	2	5	16	56	16	30	102	2	0	23	6	21	26	0	0	5	1	4	5	3	0	30	8	28	34	0	0	6	4	0	0	10	2	2	1	1	1	1	6
97	Shewanella algidipiscicola H1	65	5	0	70	34	10	26	8	2	6	17	nd	nd	nd	nd	0	0	21	9	18	22	0	0	5	2	4	5	nd	nd	nd	nd	nd	nd	0	0	10	5	0	0	15	3	2	1	1	1	1	6
98	Shewanella amazonensis SB2B	59	6	0	65	36	11	18	8	3	4	15	61	19	30	109	2	0	14	9	18	22	0	0	3	2	4	5	3	0	24	15	30	37	0	0	4	3	0	0	7	3	2	1	1	1	1	6
99	Shewanella atlantica HAW-EB5	111	12	1	122	50	11	61	9	2	11	23	60	13	73	147	1	0	40	14	27	40	0	0	7	3	5	7	1	0	48	17	32	48	1	0	14	9	0	0	23	3	2	1	1	1	1	6
84	Shewanella baltica OS678	76	10	0	86	54	10	22	10	2	4	16	nd	nd	nd	nd	0	0	22	14	29	21	0	0	4	3	5	4	nd	nd	nd	nd	nd	nd	0	0	9	4	0	0	13	3	2	1	1	1	1	6
100	Shewanella canadensis HAW-EB2	113	13	0	126	52	12	62	9	2	11	22	54	12	64	131	2	0	38	15	28	43	0	0	7	3	5	8	2	0	40	16	29	45	0	0	16	9	0	0	25	4	2	1	1	1	1	6

70	Shewanella carassii TUM17387	69	8	0	77	39	10	28	9	2	6	17	nd	nd	nd	nd	0	0	21	10	19	27	0	0	5	2	4	6	nd	nd	nd	nd	nd	nd	0	0	10	3	0	0	13	3	2	1	1	1	1	6
101	Shewanella chilikensis JC5	63	9	0	72	34	11	27	8	3	6	16	44	14	35	93	0	0	18	8	14	32	0	0	4	2	3	7	0	0	23	10	18	41	0	0	9	4	0	0	13	3	2	1	1	1	1	6
85	Shewanella denitrificans OS217	38	11	0	49	39	8	2	9	2	0	11	47	10	2	59	1	0	9	8	16	15	0	0	2	2	4	3	1	0	11	10	19	18	0	0	0	0	0	0	0	1	1	2	1	1	1	6
86	Shewanella dokdonensis DSM 23626	49	10	1	58	36	6	16	9	1	4	14	108	18	48	174	1	0	14	8	18	17	0	0	3	2	4	4	3	0	42	24	54	51	0	0	9	2	0	1	12	1	1	1	1	0	0	3
102	Shewanella donghaensis LT17	72	4	0	76	35	14	27	7	3	5	15	84	34	65	183	2	0	22	12	14	26	0	0	4	2	3	5	5	0	53	29	34	63	0	0	7	2	0	0	9	3	2	1	1	1	1	6
103	Shewanella fidelis ATCC BAA-318	82	9	2	89	41	12	36	9	3	8	19	71	21	62	154	1	0	23	15	20	30	0	0	5	3	4	6	2	0	40	26	35	52	0	0	12	5	0	0	17	4	2	1	1	1	1	6
123	Shewanella fodinae KCTC 22506	62	13	1	74	46	3	25	12	1	6	19	42	3	23	67	1	0	19	10	24	20	0	0	5	3	6	5	1	0	17	9	22	18	0	0	13	3	0	1	17	1	2	1	1	1	1	6
87	Shewanella frigidimarina NCIMB 400	66	13	0	79	44	12	23	9	2	5	16	nd	nd	nd	nd	1	0	20	16	21	21	0	0	4	3	4	4	nd	nd	nd	nd	nd	nd	0	0	5	4	0	0	9	3	2	1	1	1	1	6
71	Shewanella gelidii JCM 30804	57	7	0	64	34	9	21	9	2	5	16	38	10	24	72	0	0	17	7	18	22	0	0	4	2	5	6	0	0	19	8	20	25	0	0	5	3	0	0	8	2	2	1	1	1	1	6
125	Shewanella glacialimarina TZS-4	54	8	0	62	36	12	14	8	3	3	14	92	31	36	158	1	0	16	9	18	18	0	0	4	2	4	4	3	0	41	23	46	46	0	0	1	1	0	0	2	3	2	1	1	1	1	6
72	Shewanella glacialipiscicola T147	67	8	0	75	44	12	19	11	3	5	18	141	39	61	241	1	0	21	11	26	16	0	0	5	3	6	4	3	0	68	35	84	51	0	0	8	3	0	0	11	3	2	1	1	1	1	6
73	Shewanella hafniensis ATCC BAA-1207	67	8	0	75	46	10	19	9	2	4	15	148	32	61	241	0	0	20	11	24	20	0	0	4	2	5	4	0	0	64	35	77	64	0	0	6	3	0	0	9	3	2	2	1	1	1	7
120	Shewanella halifaxensis HAW-EB4	80	13	2	91	43	13	35	8	2	7	17	61	18	50	129	2	0	28	13	25	23	0	0	5	2	5	4	3	0	40	18	35	33	0	0	15	4	0	0	19	3	2	1	1	1	1	6
118	Shewanella hanedai JCM 20706	78	13	0	91	53	17	21	9	3	4	15	nd	nd	nd	nd	1	0	23	14	23	30	0	0	4	2	4	5	nd	nd	nd	nd	nd	nd	0	0	10	4	0	0	14	3	2	1	1	1	1	6
104	Shewanella indica KJW27	64	8	0	72	34	11	27	8	2	6	16	69	22	55	145	0	0	19	9	16	28	0	0	4	2	4	6	0	0	38	18	32	57	0	0	8	4	0	0	12	3	2	1	1	1	1	6
78	Shewanella intestini MCCC 1A01895	58	6	0	64	27	7	30	7	2	7	16	33	9	37	79	1	0	20	13	11	19	0	0	5	3	3	5	1	0	25	16	14	24	0	0	13	5	0	0	18	3	2	1	1	1	1	6
105	Shewanella inventionis CGMCC 1.15339	53	12	0	65	39	10	16	8	2	3	13	47	12	19	78	0	0	14	9	19	23	0	0	3	2	4	5	0	0	17	11	23	28	0	0	1	1	0	0	2	2	2	1	1	1	1	6
88	Shewanella japonica KCTC 22435 (=ATCC: BAA-316)	79	7	0	86	38	19	29	8	4	6	17	66	33	50	149	1	1	24	11	14	35	0	0	5	2	3	7	2	2	42	19	24	61	0	0	9	3	0	0	12	3	2	1	1	1	1	6
74	Shewanella khirikhana TH2012	59	8	0	67	38	10	19	8	2	4	14	nd	nd	nd	nd	0	1	15	9	20	22	0	0	3	2	4	5	nd	nd	nd	nd	nd	nd	0	0	4	2	0	0	6	4	2	1	1	1	1	6
89	Shewanella litoralis JCM 32306	49	13	0	62	35	9	18	8	2	4	13	22	6	12	40	1	0	16	7	17	21	0	0	3	2	4	5	1	0	10	4	11	13	0	0	5	3	0	0	8	2	2	1	1	1	1	6
106	Shewanella litorisediminis SMK1-12	55	5	0	60	32	9	19	8	2	4	14	55	16	33	104	0	0	16	8	16	20	0	0	4	2	4	5	0	0	28	14	28	35	0	0	4	3	0	0	7	3	2	1	1	1	1	6
90	Shewanella livingstonensis LMG 19866	60	17	1	76	45	11	20	9	2	4	16	179	44	80	303	1	0	16	12	25	22	0	0	3	2	5	5	4	0	64	48	100	88	0	0	4	4	0	0	8	2	2	1	1	1	1	6
107	Shewanella mangrovi YQH10	57	12	0	69	39	5	25	9	1	6	16	nd	nd	nd	nd	2	0	18	8	20	21	0	0	4	2	5	5	nd	nd	nd	nd	nd	nd	0	0	9	3	0	0	12	1	2	1	1	1	1	6
91	Shewanella marina JCM 15074	70	11	1	80	49	10	21	11	2	5	18	181	37	78	296	1	0	22	11	24	22	0	0	5	2	5	5	4	0	81	41	89	81	0	0	11	4	0	0	15	4	2	1	1	1	1	6
108	Shewanella marisflavi EP1	74	8	0	82	41	12	29	9	3	7	19	38	11	27	76	3	0	24	11	23	21	1	0	6	3	5	5	3	0	22	10	21	19	0	0	6	4	0	0	10	3	2	1	1	1	1	6
92	Shewanella maritima D4-2	59	6	0	65	29	14	22	6	3	5	14	78	37	59	174	2	0	20	12	12	19	0	0	4	3	3	4	5	0	53	32	32	51	0	0	10	2	0	1	13	2	2	1	1	0	1	5
75	Shewanella morhuae ATCC BAA-1205	65	8	1	72	41	12	19	10	3	5	17	132	39	61	232	2	0	17	11	26	16	0	0	4	3	6	4	6	0	55	35	84	51	0	0	6	2	0	0	8	3	2	1	1	1	1	6
126	Shewanella nanhaiensis NR704-98	80	10	0	90	54	14	22	10	2	4	16	59	15	24	99	2	0	21	13	25	29	0	0	4	2	4	5	2	0	23	14	27	32	0	0	9	2	0	0	11	4	2	1	1	1	1	6

109	Shewanella oneidensis MR-1	70	10	0	80	46	12	22	9	2	4	16	77	20	37	135	0	0	22	7	26	25	0	0	4	1	5	5	0	0	37	12	44	42	0	0	7	4	0	0	11	2	2	1	1	1	1	6
76	Shewanella pealeana ATCC 700345	78	11	2	87	39	11	37	8	2	7	17	79	22	75	177	1	0	30	12	21	23	0	0	6	2	4	4	2	0	61	24	43	47	0	0	12	5	0	0	17	3	2	1	1	1	1	6
110	Shewanella piezotolerans WP3	77	12	1	88	39	17	32	7	3	6	16	34	15	28	76	3	0	33	12	16	24	1	0	6	2	3	4	3	0	28	10	14	21	0	0	10	4	0	0	14	3	2	1	1	1	1	6
77	Shewanella polaris SM1901	61	12	1	72	37	12	23	8	3	5	15	22	7	14	44	2	0	19	9	17	25	0	0	4	2	4	5	1	0	12	5	10	15	0	0	5	2	0	1	8	2	2	1	1	1	1	6
111	Shewanella psychrophila WP2	74	11	0	85	52	15	18	8	2	3	13	45	13	16	73	2	1	24	14	19	25	0	0	4	2	3	4	2	1	21	12	16	22	0	0	3	2	0	0	5	2	2	2	1	1	1	7
67	Shewanella putrefaciens ATCC 8071	59	6	0	65	36	10	19	8	2	4	15	nd	nd	nd	nd	1	0	21	8	21	14	0	0	5	2	5	3	nd	nd	nd	nd	nd	nd	0	0	6	2	0	0	8	2	2	1	1	1	1	6
80	Shewanella sairae JCM 11563	78	7	2	83	39	11	33	7	2	6	15	47	13	40	100	1	0	26	12	18	26	0	0	5	2	3	5	1	0	31	14	22	31	0	0	8	3	0	0	11	4	2	1	1	1	1	6
93	Shewanella saliphila JCM 32304	54	16	0	70	37	11	22	8	2	5	15	29	9	17	55	3	0	17	5	20	25	1	0	4	1	4	5	2	0	13	4	16	20	0	0	6	2	0	0	8	2	2	1	1	1	1	6
81	Shewanella schlegeliana JCM 11561	83	11	1	93	40	11	42	8	2	9	19	48	13	51	112	2	0	29	12	17	33	0	0	6	2	3	7	2	0	35	14	20	40	0	0	11	5	0	0	16	3	2	1	1	1	1	6
112	Shewanella sediminis HAW-EB3	114	13	1	126	51	12	63	9	2	11	23	66	16	82	163	2	0	45	14	28	37	0	0	8	3	5	7	3	0	58	18	36	48	1	0	19	12	0	0	31	5	2	2	1	1	1	7
124	Shewanella sp. ANA-3	68	12	0	80	48	13	19	9	2	4	15	nd	nd	nd	nd	0	0	21	8	24	27	0	0	4	2	5	5	nd	nd	nd	nd	nd	nd	0	0	9	3	0	0	12	3	2	1	1	1	1	6
113	Shewanella sp. Arc9-LZ	62	14	0	76	46	12	18	9	2	4	15	nd	nd	nd	nd	0	0	15	15	25	21	0	0	3	3	5	4	nd	nd	nd	nd	nd	nd	0	0	4	3	0	0	7	3	2	1	1	1	1	6
122	Shewanella sp. BF02_Schw	68	16	0	84	54	11	19	10	2	4	16	nd	nd	nd	nd	1	0	15	18	28	22	0	0	3	3	5	4	nd	nd	nd	nd	nd	nd	0	0	4	3	0	0	7	3	2	1	1	1	1	6
119	Shewanella sp. HN-41	58	6	0	64	35	12	17	8	3	4	14	nd	nd	nd	nd	0	0	16	7	20	21	0	0	4	2	5	5	nd	nd	nd	nd	nd	nd	0	0	6	3	0	0	9	2	2	0	1	1	1	5
121	Shewanella sp. LZH-2	76	8	0	84	46	13	25	10	3	5	18	nd	nd	nd	nd	2	0	26	9	26	21	0	0	6	2	6	5	nd	nd	nd	nd	nd	nd	0	0	10	4	0	0	14	3	2	1	1	1	1	6
114	Shewanella sp. M16	74	9	0	83	48	11	24	9	2	5	16	nd	nd	nd	nd	0	0	28	10	22	23	0	0	5	2	4	4	nd	nd	nd	nd	nd	nd	0	0	9	4	0	0	13	2	2	1	1	1	1	6
82	Shewanella sp. MR-4	67	8	0	75	42	14	19	9	3	4	16	nd	nd	nd	nd	1	0	18	10	20	26	0	0	4	2	4	6	nd	nd	nd	nd	nd	nd	0	0	9	3	0	0	12	3	2	1	1	1	1	6
83	Shewanella sp. TC10	83	7	0	90	36	17	37	7	3	7	18	nd	nd	nd	nd	3	0	21	11	17	38	1	0	4	2	3	8	nd	nd	nd	nd	nd	nd	0	0	11	5	0	0	16	3	2	1	1	1	1	6
115	Shewanella sp. W3-18-1	60	9	0	69	41	10	18	9	2	4	15	nd	nd	nd	nd	0	0	20	8	22	19	0	0	4	2	5	4	nd	nd	nd	nd	nd	nd	0	0	7	2	0	0	9	2	2	1	1	1	1	6
94	Shewanella ulleungensis JCM 32305	54	13	0	67	38	11	18	8	2	4	14	24	7	12	43	0	0	14	9	20	24	0	0	3	2	4	5	0	0	9	6	13	15	0	0	4	2	0	0	6	2	2	1	1	1	1	6
116	Shewanella vesiculosa LMG 24424	56	15	0	71	44	9	18	9	2	4	15	100	20	41	161	0	0	15	14	23	19	0	0	3	3	5	4	0	0	34	32	52	43	0	0	2	2	0	0	4	3	2	1	1	1	1	6

117	Shewanella violacea DSS12	36	9	0	45	32	10	3	6	2	1	9	17	5	2	24	0	0	5	10	18	12	0	0	1	2	4	2	0	0	3	5	9	6	0	0	1	0	0	0	1	2	2	2	1	1	1	7
95	Shewanella woodyi ATCC 51908	82	10	0	92	54	16	22	9	3	4	16	83	24	34	141	2	0	20	13	25	32	0	0	3	2	4	5	3	0	31	20	38	49	0	0	7	3	0	0	10	4	2	1	1	1	1	6
25	Solidesulfovibrio alcoholivorans DSM 5433	57	11	2	66	49	3	14	10	1	3	13	32	2	9	44	0	0	12	11	28	15	0	0	2	2	5	3	0	0	8	7	19	10	3	9	11	0	2	0	13	2	2	1	1	0	0	4
14	Solidesulfovibrio fructosivorans JJ	54	9	1	62	49	4	9	10	1	2	13	58	5	11	73	0	0	10	13	26	13	0	0	2	3	6	3	0	0	12	15	31	15	3	10	10	0	0	0	10	0	2	1	1	0	0	4
7	Solidesulfovibrio magneticus RS-1	58	17	3	72	53	5	14	10	1	3	14	17	2	4	23	0	0	13	16	28	15	0	0	2	3	5	3	0	0	4	5	9	5	3	5	15	0	1	0	16	1	2	1	1	0	0	4
